# Prevalence of sexually transmitted infections and bacterial vaginosis among women in sub-Saharan Africa: An individual participant data meta-analysis of 18 HIV prevention studies

**DOI:** 10.1371/journal.pmed.1002511

**Published:** 2018-02-27

**Authors:** Elizabeth A. Torrone, Charles S. Morrison, Pai-Lien Chen, Cynthia Kwok, Suzanna C. Francis, Richard J. Hayes, Katharine J. Looker, Sheena McCormack, Nuala McGrath, Janneke H. H. M. van de Wijgert, Deborah Watson-Jones, Nicola Low, Sami L. Gottlieb, on behalf of the STIMA Working Group

**Affiliations:** 1 Division of STD Prevention, Centers for Disease Control and Prevention, Atlanta, Georgia, United States of America; 2 Global Health, Population and Nutrition, FHI 360, Durham, North Carolina, United States of America; 3 Tropical Epidemiology Group, London School of Hygiene & Tropical Medicine, London, United Kingdom; 4 Population Health Sciences, Bristol Medical School, University of Bristol, Bristol, United Kingdom; 5 MRC Clinical Trials Unit, University College London, London, United Kingdom; 6 Academic Unit of Primary Care and Population Sciences, University of Southampton, Southampton, United Kingdom; 7 Department of Social Statistics and Demography, University of Southampton, Southampton, United Kingdom; 8 Africa Health Research Institute, School of Nursing and Public Health, University of KwaZulu-Natal, Durban, South Africa; 9 Research Department of Epidemiology & Public Health, University College London, London, United Kingdom; 10 Institute of Infection and Global Health, University of Liverpool, Liverpool, United Kingdom; 11 Clinical Research Department, London School of Hygiene & Tropical Medicine, London, United Kingdom; 12 Mwanza Intervention Trials Unit, National Institute for Medical Research, Mwanza, Tanzania; 13 Institute of Social and Preventive Medicine, University of Bern, Bern, Switzerland; 14 Department of Reproductive Health and Research, World Health Organization, Geneva, Switzerland; Umeå Centre for Global Health Research, Umeå University, SWEDEN

## Abstract

**Background:**

Estimates of sexually transmitted infection (STI) prevalence are essential for efforts to prevent and control STIs. Few large STI prevalence studies exist, especially for low- and middle-income countries (LMICs). Our primary objective was to estimate the prevalence of chlamydia, gonorrhea, trichomoniasis, syphilis, herpes simplex virus type 2 (HSV-2), and bacterial vaginosis (BV) among women in sub-Saharan Africa by age, region, and population type.

**Methods and findings:**

We analyzed individual-level data from 18 HIV prevention studies (cohort studies and randomized controlled trials; conducted during 1993–2011), representing >37,000 women, that tested participants for ≥1 selected STIs or BV at baseline. We used a 2-stage meta-analysis to combine data. After calculating the proportion of participants with each infection and standard error by study, we used a random-effects model to obtain a summary mean prevalence of each infection and 95% confidence interval (CI) across ages, regions, and population types. Despite substantial study heterogeneity for some STIs/populations, several patterns emerged. Across the three primary region/population groups (South Africa community-based, Southern/Eastern Africa community-based, and Eastern Africa higher-risk), prevalence was higher among 15–24-year-old than 25–49-year-old women for all STIs except HSV-2. In general, higher-risk populations had greater prevalence of gonorrhea and syphilis than clinic/community-based populations. For chlamydia, prevalence among 15–24-year-olds was 10.3% (95% CI: 7.4%, 14.1%; *I*^2^ = 75.7%) among women specifically recruited from higher-risk settings for HIV in Eastern Africa and was 15.1% (95% CI: 12.7%, 17.8%; *I*^2^ = 82.3%) in South African clinic/community-based populations. Among clinic/community-based populations, prevalence was generally greater in South Africa than in Southern/Eastern Africa for most STIs; for gonorrhea, prevalence among 15–24-year-olds was 4.6% (95% CI: 3.3%, 6.4%; *I*^2^ = 82.8%) in South Africa and was 1.7% (95% CI: 1.2%, 2.6%; *I*^2^ = 55.2%) in Southern/Eastern Africa. Across the three primary region/population groups, HSV-2 and BV prevalence was high among 25–49-year-olds (ranging from 70% to 83% and 33% to 44%, respectively). The main study limitation is that the data are not from random samples of the target populations.

**Conclusions:**

Combining data from 18 HIV prevention studies, our findings highlight important features of STI/BV epidemiology among sub-Saharan African women. This methodology can be used where routine STI surveillance is limited and offers a new approach to obtaining critical information on STI and BV prevalence in LMICs.

## Abstract 2

In a meta-analysis of observational studies and randomized trials, Elizabeth A. Torrone and colleagues provide estimates of STI and BV prevalence among populations of sub-Saharan African women.

## Author summary

**Why was this study done?:**

- Sexually transmitted infections (STIs) and bacterial vaginosis (BV) can result in a range of sexual, reproductive, and maternal-child health consequences.
- Few population-based studies of STI/BV prevalence exist for low- and middle-income countries.
- Many HIV prevention studies gather baseline data on STIs, and, although not the primary objective of the studies, these data may be useful for estimating STI/BV prevalence.

**What did the researchers do and find?:**

- We used individual-level data from 18 HIV prevention studies conducted in sub-Saharan Africa, which tested over 37,000 HIV-negative women for 1 or more STIs or BV at baseline.
- Using a 2-stage meta-analysis, we estimated the prevalence of 5 STIs (chlamydia, gonorrhea, trichomoniasis, syphilis, and herpes simplex virus type 2 [HSV-2]) and BV across studies by age, region, and population type.
- STI prevalence was highest among young women aged 15–24 years for all STIs except HSV-2, regardless of region or population type.
- Estimated STI/BV prevalence often varied by region. For example, chlamydia and gonorrhea prevalence was higher among South African women enrolled from clinic/community settings than among similar populations elsewhere in Southern/Eastern Africa.
- Some STIs, such as syphilis, appeared to be primarily concentrated among higher-risk populations.

**What do these findings mean?:**

- Findings from this study strengthen our understanding of STI/BV epidemiology among sub-Saharan African women.
- By identifying differences in STI/BV prevalence by age, region, and population type, this analysis addresses the first pillar of the World Health Organization Global Health Sector Strategy on Sexually Transmitted Infections 2016–2021: to increase information for focused public health action.
- Existing data from research studies provide an opportunity to obtain critical information on STI/BV prevalence and can be used where routine STI surveillance and stand-alone STI studies are limited.

## Introduction

Sexually transmitted infections (STIs) and bacterial vaginosis (BV) are widespread globally. These conditions have important sexual, reproductive, and maternal-child health consequences, including genital symptoms, pregnancy complications, infertility, enhanced HIV transmission, and psychosocial effects. The World Health Organization (WHO) estimated that, in 2012, there were 357 million new episodes of 4 curable STIs (chlamydia, gonorrhea, syphilis, and trichomoniasis) [1] and 417 million people had infection with herpes simplex virus type 2 (HSV-2) [2]. Global estimates of BV occurrence are limited [3].

The WHO Global Health Sector Strategy on Sexually Transmitted Infections 2016–2021 (Global Strategy), provides goals, targets, and priority actions for stemming the STI epidemic [4]. As data on STI burden are critical for efforts to prevent, control, and manage STIs, the first strategic direction of the Global Strategy is to increase information, including STI prevalence estimates, for focused public health action. Estimating the global burden of STIs is hampered by the limited availability of STI diagnostic testing and surveillance and a paucity of high-quality published studies of STI prevalence in low- and middle-income countries (LMICs), for both general and key populations. Given these limitations, a WHO Consultation on Methods for Improved Global STI Estimates highlighted the importance of exploring potential data available through clinical research studies and trial networks to improve estimates [5], because many studies gather baseline data on STIs, even when estimating STI prevalence is not a primary study objective.

In one collection of such studies, investigators used combined data from 18 prospective HIV prevention studies in sub-Saharan Africa in an individual participant data (IPD) meta-analysis to examine the association between hormonal contraceptive (HC) use and HIV acquisition [6]. Although the main objective of these studies was not to estimate STI/BV prevalence, all of them captured information on at least some STIs or BV. The 18 studies in the HC-HIV IPD dataset include more than 37,000 women in 9 countries in sub-Saharan Africa [7–33] and present a unique opportunity to explore prevalence of selected sexually transmitted and reproductive tract infections.

In the current study, we used IPD meta-analysis to estimate the prevalence of selected STIs and BV, and the prevalence of co-infections, among women in sub-Saharan Africa participating in these 18 studies. We selected STIs included in the majority of the studies (chlamydia, gonorrhea, trichomoniasis, syphilis, and HSV-2), and present STI/BV prevalence by age, region, and population type.

## Methods

We followed a protocol and a prespecified analysis plan (S3 Text). We report our findings in accordance with the Preferred Reporting Items for Systematic Reviews and Meta-Analyses (PRISMA) (S1 Text) and provide a checklist of items specific to IPD meta-analyses (S2 Text). PRISMA checklists for previous IPD analyses of these data are also available [6,34]. The US Centers for Disease Control and Prevention, WHO, and the Protection of Human Subjects Committee of FHI 360 determined that the research was exempt from ethical review. All included studies had relevant country-specific institutional ethical review and regulatory board approvals, and all participants within each study provided written informed consent for study participation. All principal investigators agreed to share their data for the present meta-analysis.

### Study eligibility and inclusion criteria

The collection of studies included in the current analysis started as the Vaginal Practices Research Partnership, in which investigators combined data from 10 cohort studies and HIV prevention trials in sub-Saharan Africa in an IPD meta-analysis to examine associations of women’s intravaginal practices and acquisition of HIV [34]. Data from 8 additional studies were then added for the evaluation of the association between HC and HIV acquisition [6]. The final 18 studies in the HC-HIV IPD meta-analysis included HIV-negative women recruited from general populations and groups of women identified to be at higher-risk of HIV infection, such as women engaging in transactional sex. Women were not randomly sampled. Initial inclusion and exclusion criteria for the IPD meta-analysis of HC and HIV acquisition are listed in S1 Table. In general, studies had to include women between the ages of 15 and 49 years, measure HIV prospectively at multiple time points, and measure HC use.

To minimize the risk of measurement bias, additional inclusion criteria for the current IPD meta-analysis to estimate STI/BV prevalence were applied separately for chlamydia, gonorrhea, trichomoniasis, syphilis, HSV-2, and BV. To be included, the study had to have (1) measured the STI/BV at baseline as part of its protocol, (2) provided information about both the diagnostic test and the test result, and (3) tested at least 80% of all participants in the study for the STI/BV or tested at least 80% of a randomly selected sample of enrolled participants. Studies were excluded from prevalence estimation for a specific STI/BV if (1) the study design required the STI/BV diagnosis in question for study inclusion (e.g., participants required to be HSV-2 antibody positive) or (2) >10% of those tested for the STI/BV had indeterminate test results.

### Data collection, data management, and data items

The investigators of each eligible study gave us permission to use their data in the STI meta-analysis. We included variables at both study and individual level. Study-level variables included country, study design, population types, study aims, recruitment period, and diagnostic procedures. Individual-level variables included sociodemographic variables (age, education, marital status, employment status, number of live births) and sexual behavior variables (condom use at last sex, age at first sex, coital frequency, number of lifetime partners, new partners, concurrent partners, and contraceptive use). The study team worked closely with the data managers and investigators of the individual studies to clarify issues about variable definition and missing, incomplete, or implausible data.

### Definition of prevalent STI or BV

Prevalent STI was defined, based on the baseline study visit, as a positive test result by prespecified tests for each pathogen: *Chlamydia trachomatis* (chlamydia)—nucleic acid amplification test (NAAT), enzyme immunoassay (EIA) test, or hybrid capture test; *Neisseria gonorrhoeae* (gonorrhea)—NAAT or culture; *Trichomonas vaginalis* (trichomoniasis)—NAAT, InPouch (BioMed Diagnostics, White City, OR) culture, or wet mount; HSV-2 infection—type-specific serologic test; *Treponema pallidum* (syphilis)—any rapid plasma reagin (RPR) and a treponemal test (*Treponema pallidum* hemagglutination assay [TPHA], *Treponema pallidum* particle agglutination assay [TPPA], or Determine Rapid Syphilis TP Assay [Abbott Laboratories, Tokyo, Japan]). A subset of studies recorded RPR titer, and for these studies, we defined high-titer, active syphilis as an RPR titer ≥ 1:8 and a positive treponemal test [35]. Prevalent BV diagnosis was based on Nugent score ≥ 7 or Ison-Hay grade ≥ grade III.

### Stratification of prevalence estimates

We initially selected 5 factors likely to influence STI/BV prevalence for stratification: participant age, region, population type, time period, and pregnancy status. After preliminary examination of the available data, we stratified prevalence estimates only by participant age, region, and study population type. We decided not to stratify studies by time period or pregnancy status given limited sample size.

We categorized age as 15–24 years or 25–49 years, according to individual participant age at the baseline visit. We defined 3 regions (based on geographical location of the study): South Africa, Eastern Africa (Kenya, Uganda, Tanzania, Rwanda), and Southern Africa (Zambia, Zimbabwe, Malawi, Botswana). Studies that were conducted in more than 1 region were split into sub-studies that included only a single region. We defined 3 study population types (based on enrollment and inclusion criteria for each study): clinic/community-based populations, higher-risk populations, and HIV-discordant couples. Clinics were family planning and reproductive health clinics and enrolled women regardless of symptom status. Community-based settings included enrollment at community meetings or house-to-house visits. Higher-risk populations included studies where participants were recruited from bars or other recreational facilities like guesthouses and hotels that were considered high-risk venues for acquiring HIV infection, or were recruited because they engaged in transactional sex. Studies of HIV-discordant couples included the HIV-negative partners of HIV-discordant partnerships. Studies that included participants from multiple population types were split into multiple sub-studies for analysis.

### Analysis

#### Descriptive analysis

Participant characteristics (sociodemographic characteristics, risk behaviors, etc.) were compared across the strata of participant age, region, and population type.

#### Prevalence estimation and meta-analysis

Using individual-level participant data, we used 2-stage meta-analysis to derive summary estimates of the mean prevalence of each infection according to the prespecified strata of age group, region, and study population type. In the first stage, we used the data from participants in each individual study to obtain the proportion infected and standard error on the logit scale. Some strata contained 1 or more studies with 0 infections, for which the logarithm is undefined. For all studies in these strata, we used the observed proportion and estimated the standard error using the exact binomial confidence limits. In the second stage, we used a random-effects model to pool the log odds of infection across studies, weighted by the variability of the estimate of the individual studies. The pooled log odds and 95% confidence interval (CI) were then transformed to obtain summary prevalence estimates on the natural scale. We generated forest plots in R and inspected them visually to compare prevalence estimates across strata, and combined strata if estimates were similar. We used the *I*^2^ statistic to describe the percentage of variability in prevalence estimates resulting from between-study heterogeneity rather than sampling error. We applied approximate categories of *I*^2^ values to determine low heterogeneity (*I*^2^ < 50%), mild or moderate heterogeneity (*I*^2^ from 50% to 75%), and high heterogeneity (*I*^2^ > 75%) and used these categories in the interpretation of summary estimates of prevalence [36].

#### Prevalence of co-infection

We investigated the prevalence of co-infection among women tested for each combination of infections (e.g., tested for both HSV-2 and gonorrhea). For each infection, we estimated the prevalence of co-infection with an additional infection (e.g., among women with HSV-2, the prevalence of gonorrhea) among women tested for both infections. Co-infection prevalence was estimated for each stratum using the methods described above.

## Results

All 18 potentially eligible studies included testing for at least 1 STI and/or BV testing at baseline. Test technologies varied across studies (S2 Table). Five studies included multiple geographic regions and/or population types and were split into separate sub-studies for analysis, resulting in a total of 26 studies/sub-studies (hereafter referred to as “studies”) (Table 1). There were 10 studies in South Africa that were conducted among clinic/community-based populations (*N =* 9) and HIV-discordant couples (*N =* 1). Nine studies were conducted in Southern Africa (*N =* 5) or Eastern Africa (*N =* 4) among clinic/community-based populations (*N =* 7) and HIV-discordant couples (*N =* 2). Seven studies were conducted in Eastern Africa among higher-risk populations. Sample sizes of included studies ranged from 138 to 5,654.

**Table 1:** Summary characteristics of included studies.

Study ID [References]	Country	Region	Study population	Population type category	Study design; objective	Study period	Ages included, years	Median age, years	*N* included
S1 [7,8]	Kenya	Eastern Africa	Women who engage in transactional sex	Higher-risk	Cohort; hormonal contraception and HIV	1993–2002	16–48	26	1,219
S2 [9,10]	South Africa	South Africa	Women not screened for cervical cancer	Clinic/ community-based	RCT; cervical cancer screening interventions	2000–2002	35–49	40	4,158
S3a [11,12]	Uganda	Eastern Africa	Women attending reproductive health clinics	Clinic/ community-based	Cohort; hormonal contraception and HIV	1999–2002	18–35	24	2,194
S3b [11,12]	Zimbabwe	Southern Africa	Women attending reproductive health clinics	Clinic/ community-based	Cohort; hormonal contraception and HIV	1999–2002	18–35	26	2,231
S4 [13]	Kenya	Eastern Africa	Women who engage in transactional sex	Higher-risk	RCT; presumptive antibiotic treatment	1998–2002	18–49	27	399
S5 [14]	Tanzania	Eastern Africa	Women working in bars	Higher-risk	Cohort; microbicide feasibility study	2002–2003	15–49	28	930
S6 [15,16]	Tanzania	Eastern Africa	Women working in bars and other recreational facilities	Higher-risk	RCT; HSV suppression to reduce HIV transmission	2003–2006	16–35	28	769
S7a [17,18]	Zimbabwe	Southern Africa	Sexually active women	Clinic/ community-based	RCT; diaphragm/condoms to reduce HIV acquisition	2003–2005	18–49	28	1,648
S7b [17,18]	South Africa	South Africa	Sexually active women	Clinic/ community-based	RCT; diaphragm/condoms to reduce HIV acquisition	2003–2005	18–49	27	2,426
S8 [19]	South Africa	South Africa	Women attending reproductive health clinics	Clinic/ community-based	Cohort; hormonal contraception and HIV	1999–2001	18–41	27	545
S9 [20]	South Africa	South Africa	Women attending clinics	Clinic/ community-based	Cohort; microbicide feasibility study	2002–2004	18–35	23	690
S10 [21]	South Africa	South Africa	Women attending clinics	Clinic/ community-based	Cohort; microbicide feasibility study	2003–2004	15–49	27	256
S11 [22,23]	Malawi, Zimbabwe	Southern Africa	Women attending clinics	Clinic/ community-based	Cohort; microbicide feasibility study	2001–2002	18–49	26	1,423
S12 [24,25]	South Africa	South Africa	Sexually active women	Clinic/ community-based	RCT; microbicide to reduce HIV acquisition	2004–2006	16–49	28	5,615
S13 [26]	Uganda	Eastern Africa	Women who engage in transactional sex	Higher-risk	Cohort; microbicide feasibility study	2008–2009	15–49	26	418
S14 [27]	Tanzania	Eastern Africa	Women working in food and recreational facilities	Higher-risk	Cohort; microbicide feasibility study	2008–2009	18–44	27	873
S15a [28,29]	Kenya, Uganda, Rwanda, Tanzania	Eastern Africa	Sexually active women whose partners had HIV and HSV-2	HIV-discordant couples	RCT; HSV suppression to reduce HIV transmission	2004–2009	18–49	29	913
S15b [28,29]	Botswana, Zambia	Southern Africa	Sexually active women whose partners had HIV and HSV-2	HIV-discordant couples	RCT; HSV suppression to reduce HIV transmission	2004–2009	18–49	31	214
S15c [28,29]	South Africa	South Africa	Sexually active women whose partners had HIV and HSV-2	HIV-discordant couples	RCT; HSV suppression to reduce HIV transmission	2004–2009	18–49	32	138
S16a [30,31]	Tanzania	Eastern Africa	Women working in bars and other recreational facilities	Higher-risk	RCT; microbicide to reduce HIV acquisition	2005–2008	16–49	29	1,018
S16b [30,31]	Uganda	Eastern Africa	Women in HIV-discordant partnerships	HIV-discordant couples	RCT; microbicide to reduce HIV acquisition	2005–2008	16–49	31	774
S16c [30,31]	Zambia	Southern Africa	Sexually active women	Clinic/ community-based	RCT; microbicide to reduce HIV acquisition	2005–2008	18–49	27	1,150
S16d [30,31]	South Africa	South Africa	Sexually active women	Clinic/ community-based	RCT; microbicide to reduce HIV acquisition	2005–2008	17–49	26	5,654
S17 [32]	South Africa	South Africa	Sexually active women	Clinic/ community-based	RCT; microbicide to reduce HIV acquisition	2007–2009	18–40	22	444
S18a [33]	Kenya, Tanzania	Eastern Africa	Sexually active women	Clinic/ community-based	RCT; microbicide to reduce HIV acquisition	2009–2011	18–35	26	380
S18b [33]	South Africa	South Africa	Sexually active women	Clinic/ community-based	RCT; microbicide to reduce HIV acquisition	2009–2011	18–35	22	639

HSV, herpes simplex virus; HSV-2, herpes simplex virus type 2; RCT, randomized controlled trial.

Prevalence estimates from studies of HIV-discordant couples were similar to those from studies of clinic/community-based populations, so we combined these population types (subsequently referred to as “clinic/community-based studies”). In addition, prevalence estimates from clinic/community-based studies in Southern and Eastern Africa were similar, and these groups were combined (subsequently referred to as “Southern/Eastern Africa”). Our main results are presented by age group (15–24 and 25–49 years) for South African clinic/community-based populations, Southern/Eastern African clinic/community-based populations, and Eastern African higher-risk populations. Participant characteristics and STI/BV prevalence estimates according to the initial stratification variables for age, region, and population type are shown in S3 Table and S1–S8, S11–S18 Figs.

### Participant characteristics

Among 15–24-year-old participants, sociodemographics varied across the 3 region/population types (Table 2). For example, among participants recruited from clinic/community settings, participants in studies from South Africa were more likely to be unmarried (92.1%) compared with participants in studies in Southern/Eastern Africa (14.3%). As expected, participants in Eastern Africa higher-risk population studies were more likely to be sex workers and to report 2 or more recent sex partners compared with participants in clinic/community-based studies. Among 25–49-year-old participants, there were similar differences in sociodemographics and sexual behaviors across the 3 region/population types (Table 3).

**Table 2:** Baseline characteristics by region/population type among participants aged 15–24 years.

Characteristic	South African clinic/community (*N =* 7,009)	Southern/Eastern African clinic/community (*N =* 4,121)	Eastern African higher-risk (*N =* 1,942)	Total (*N =* 13,072)
***Sociodemographics***				
**Age group**				
15–19	1,956 (27.9)	736 (17.9)	419 (21.6)	3,111 (23.8)
20–24	5,053 (72.1)	3,385 (82.1)	1,523 (78.4)	9,961 (76.2)
** Total**	7,009 (100)	4,121 (100)	1,942 (100)	13,072 (100)
Missing	0	0	0	0
**Education**				
No school	41 (0.8)	122 (3.0)	137 (7.6)	300 (2.6)
Primary incomplete	110 (2.0)	588 (14.3)	421 (23.3)	1,119 (9.9)
Primary complete	123 (2.3)	715 (17.4)	825 (45.7)	1,663 (14.7)
Secondary incomplete	2,301 (42.4)	2,153 (52.2)	288 (16.0)	4,742 (41.8)
Secondary complete	2,554 (47.1)	358 (8.7)	81 (4.5)	2,993 (26.4)
Tertiary level	299 (5.5)	185 (4.5)	54 (3.0)	538 (4.7)
** Total**	5,428 (77.4)	4,121 (100)	1,806 (93.0)	11,355 (86.9)
Missing	1,581	0	136	1,717
**Married/living with partner**				
Yes	552 (7.9)	3,530 (85.7)	267 (13.8)	4,349 (33.3)
No	6,457 (92.1)	591 (14.3)	1,675 (86.3)	8,723 (66.7)
** Total**	7,009 (100)	4,121 (100)	1,942 (100)	13,072 (100)
Missing	0	0	0	0
**Employed**				
Yes	653 (12.0)	1,139 (27.6)	1,801 (99.7)	3,593 (31.7)
No	4,770 (88.0)	2,982 (72.4)	6 (0.33)	7,758 (68.4)
** Total**	5,423 (77.4)	4,121 (100)	1,807 (93.1)	11,351 (86.8)
Missing	1,586	0	135	1,721
**Number of live births**	1 (0–1)	1 (1–2)	1 (0–2)	1 (0–1)
***Sexual behaviors***				
**Condom use at last sex**				
Yes	4,213 (67.8)	1,613 (46.6)	587 (48.0)	6,413 (58.9)
No	1,998 (32.2)	1,847 (53.4)	637 (52.0)	4,482 (41.1)
** Total**	6,211 (88.6)	3,460 (84.0)	1,224 (63.0)	10,895 (83.4)
Missing	798	661	718	2,177
**Age at first sex**	17 (17–17)	17 (16–18)	17 (15–17)	17 (16–18)
**Coital frequency in past month**	8 (4–12)	12 (7–16)	4 (2–11)	8 (4–12)
**Number of lifetime partners**	2 (1–3)	1 (1–3)	3 (3–8)	2 (1–3)
New partner*				
Yes	92 (3.1)	86 (2.9)	80 (82.5)	258 (4.3)
No	2,859 (96.9)	2,881 (97.1)	17 (17.5)	5,757 (95.7)
** Total**	2,951 (42.1)	2,967 (72)	97 (5.0)	6,015 (46.0)
Missing	4,058	1,154	1,845	7,057
Concurrent partner**				
Yes	260 (4.3)	162 (5.5)	172 (22.7)	594 (6.1)
No	5,736 (95.7)	2,777 (94.5)	587 (77.3)	9,100 (93.9)
** Total**	5,996 (85.6)	2,939 (71.3)	759 (39.1)	9,694 (74.2)
Missing	1,013	1,182	1,183	3,378
**Baseline contraception**				
Non-hormonal	2,286 (32.8)	1,543 (37.5)	1,334 (68.9)	5,163 (39.6)
COCP	624 (8.9)	1,403 (34.1)	224 (11.6)	2,251 (17.3)
DMPA	2,234 (32.0)	1,148 (27.9)	378 (19.5)	3,760 (28.9)
Neten	1,835 (26.3)	16 (0.4)	0 (0.00)	1,851 (14.2)
** Total**	6,979 (99.6)	4,110 (99.7)	1,936 (99.7)	13,025 (99.6)
Multiple use	28	11	4	43
Missing	2	0	2	4

Data given as *N* (percent) or median (quartile 1–quartile 3). *N*s in this table are based on all women in included studies; prevalence estimates for each infection are based on the subset of women who were tested for that infection.

*Time period to define new partnership varied by study and was either last month or last 3 months.

**Time period to define concurrency varied by study and was either current, last month, or last 3 months.

COCP, combined oral contraceptive pill; DMPA, depot medroxyprogesterone acetate; Neten, norethisterone enanthate.

**Table 3:** Baseline characteristics by region/population type among participants aged 25–49 years.

Characteristic	South African clinic/community (*N =* 13,556)	Southern/Eastern African clinic/community (*N =* 6,806)	Eastern African higher-risk (*N =* 3,684)	Total (*N =* 24,046)
***Sociodemographics***				
**Age**	36 (31–41)	30 (27–34)	31 (27–35)	34 (28–39)
**Education**				
No school	740 (6.9)	389 (5.7)	334 (9.8)	1,463 (6.9)
Primary incomplete	1,974 (18.0)	1,292 (19.0)	796 (23.3)	4,062 (19.2)
Primary complete	1,058 (9.7)	1,538 (22.6)	1,763 (51.6)	4,359 (20.6)
Secondary incomplete	4,581 (41.9)	3,048 (44.8)	363 (10.6)	7,992 (37.8)
Secondary complete	2,026 (18.5)	371 (5.5)	105 (3.1)	2,502 (11.8)
Tertiary level	562 (5.1)	168 (2.5)	58 (1.7)	788 (3.7)
** Total**	10,941 (80.7)	6,806 (100)	3,419 (92.8)	21,166 (88.0)
Missing	2,615	0	265	2,880
**Married/living with partner**				
Yes	6,543 (48.3)	6,421 (94.3)	889 (24.1)	13,853 (57.6)
No	7,008 (51.7)	385 (5.7)	2,794 (75.9)	10,187 (42.4)
** Total**	13,551 (99.9)	6,806 (100)	3,683 (99.9)	24,040 (99.9)
Missing	5	0	1	6
**Employed**				
Yes	2,911 (26.5)	2,659 (39.1)	3,412 (99.8)	8,982 (42.3)
No	8,076 (73.5)	4,147 (60.9)	7 (0.2)	12,230 (57.7)
** Total**	10,987 (81.1)	6,806 (100)	3,419 (92.8)	21,212 (88.2)
Missing	2,569	0	265	2,834
**Number of live births**	3 (2–4)	3 (2–4)	2 (1–4)	3 (2–4)
***Sexual behaviors***				
**Condom use at last sex**				
Yes	4,776 (54.6)	2,653 (48.8)	880 (34.6)	8,309 (49.7)
No	3,967 (45.4)	2,789 (51.3)	1,665 (65.4)	8,421 (50.3)
** Total**	8,743 (64.5)	5,442 (80.0)	2,545 (69.1)	16,730 (69.6)
Missing	4,813	1,364	1,139	7,316
**Age at first sex**	17 (16–18)	17 (17–20)	17 (15–18)	17 (16–18)
**Coital frequency in past month**	6 (2–12)	12 (6–16)	4 (2–8)	8 (4–12)
**Number of lifetime partners**	3 (2–5)	1 (1–2)	5 (3–8)	3 (2–5)
New partner*				
Yes	99 (2.4)	50 (1.3)	138 (85.2)	287 (3.6)
No	3,924 (97.5)	3,854 (98.7)	24 (14.8)	7,802 (96.5)
** Total**	4,023 (29.7)	3,904 (57.4)	162 (4.4)	8,089 (33.6)
Missing	9,533	2,902	3,522	15,957
Concurrent partner**				
Yes	369 (3.1)	180 (4.0)	273 (18.0)	822 (4.6)
No	11,663 (96.9)	4,320 (96.0)	1,246 (82.0)	17,229 (95.5)
** Total**	12,032 (88.8)	4,500 (66.1)	1,519 (41.2)	18,051 (75.1)
Missing	1,524	2,306	2,165	5,995
**Baseline contraception**				
Non-hormonal	7,702 (57.1)	2,927 (43.2)	2,418 (65.0)	13,047 (54.5)
COCP	1,097 (8.1)	2,038 (30.0)	449 (12.2)	3,584 (15.0)
DMPA	3,391 (25.1)	1,768 (26.1)	803 (21.9)	5,962 (24.9)
Neten	1,298 (9.6)	51 (0.8)	0 (0.00)	1,349 (5.6)
** Total**	13,488 (99.5)	6,784 (99.7)	3,670 (99.6)	23,942 (99.6)
Multiple use	20	22	9	51
Missing	48	0	5	53

Data given as *N* (percent) or median (quartile 1–quartile 3). *N*s in this table are based on all women in included studies; prevalence estimates for each infection are based on the subset of women who were tested for that infection.

*Time period to define new partnership varied by study and was either last month or last 3 months.

**Time period to define concurrency varied by study and was either current, last month, or last 3 months.

COCP, combined oral contraceptive pill; DMPA, depot medroxyprogesterone acetate; Neten, norethisterone enanthate.

### Prevalence estimates

#### Chlamydia

**Fig 1:**
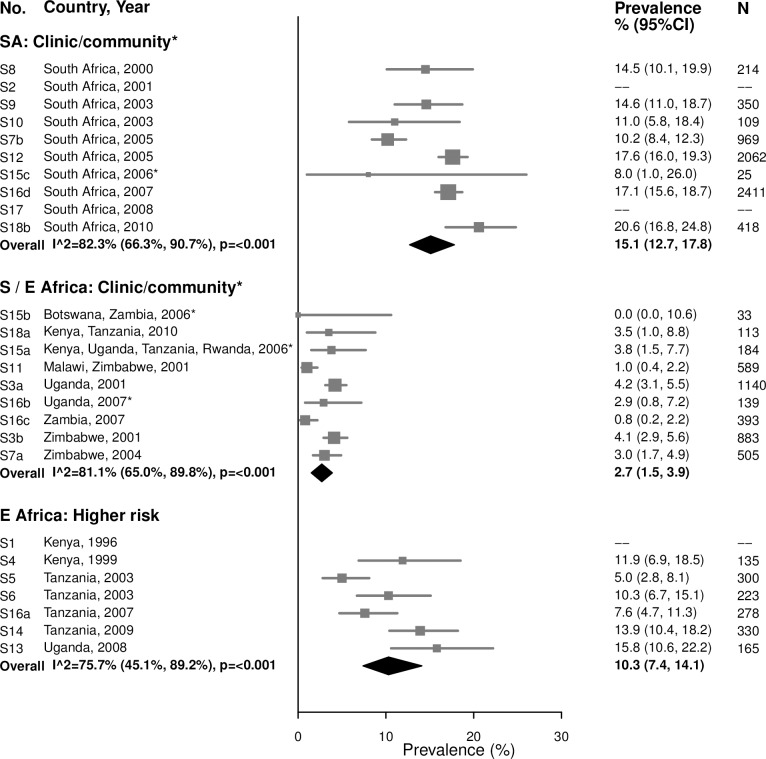
Chlamydia prevalence among 15–24-year-olds. The area of the marker is proportional to the square root of the sample size. Studies with no estimate presented did not meet the inclusion criteria for this infection and age group. Year presented is the median year that baseline data were collected during the study. *Includes studies of HIV-discordant couples. E Africa, Eastern Africa; SA, South Africa; S/E Africa, Southern and Eastern Africa.

Among women aged 15–24 years in clinic/community-based populations in South Africa, estimated chlamydia prevalence ranged from 8.0% to 20.6% (summary estimate: 15.1% [95% CI: 12.7%, 17.8%]; *I*^2^ = 82.3%), which was similar to estimated prevalence in the higher-risk populations in Eastern Africa, which ranged from 5.0% to 15.8% (summary estimate: 10.3% [95% CI: 7.4%, 14.1%]; *I*^2^ = 75.7%) (Fig 1). Prevalence was lower in the clinic/community-based populations in Southern/Eastern Africa, with a summary average estimate of 2.7% (95% CI: 1.5%, 3.9%; *I*^2^ = 81.1%).

**Fig 2:**
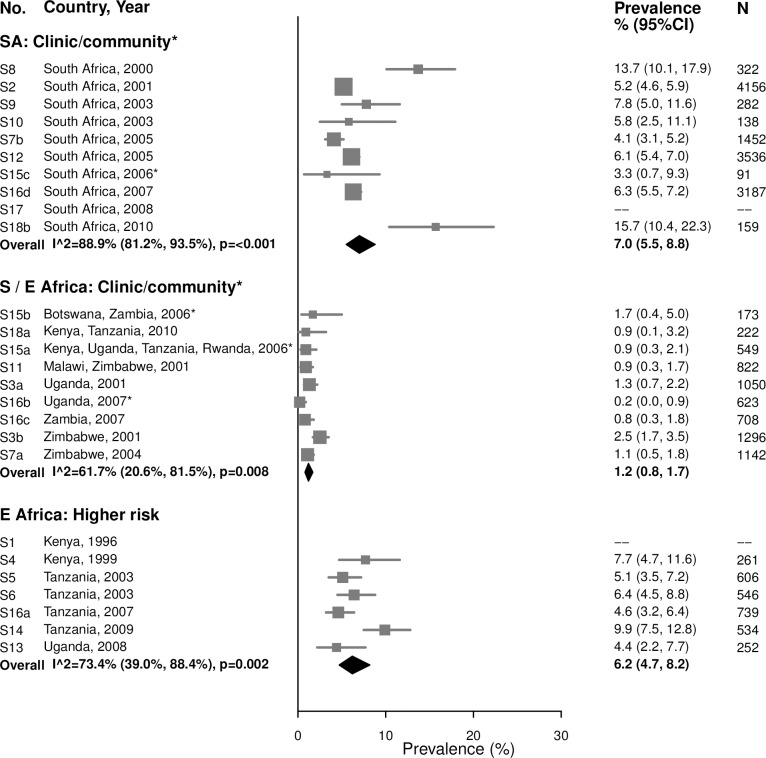
Chlamydia prevalence among 25–49-year-olds. The area of the marker is proportional to the square root of the sample size. Studies with no estimate presented did not meet the inclusion criteria for this infection and age group. Year presented is the median year that baseline data were collected during the study. *Includes studies of HIV-discordant couples. E Africa, Eastern Africa; SA, South Africa; S/E Africa, Southern and Eastern Africa.

Summary estimates of chlamydia prevalence were lower among women aged 25–49 years than among women aged 15–24 years in all region/population types (Fig 2). In the clinic/community-based populations in Southern/Eastern Africa, the summary estimate of prevalence among 25–49-year-old women was 1.2% (95% CI: 0.8%, 1.7%; *I*^2^ = 61.7%). In the higher-risk populations in Eastern Africa, the summary estimate of prevalence was 6.2% (95% CI: 4.7%, 8.2%; *I*^2^ = 73.4%).

#### Gonorrhea

**Fig 3:**
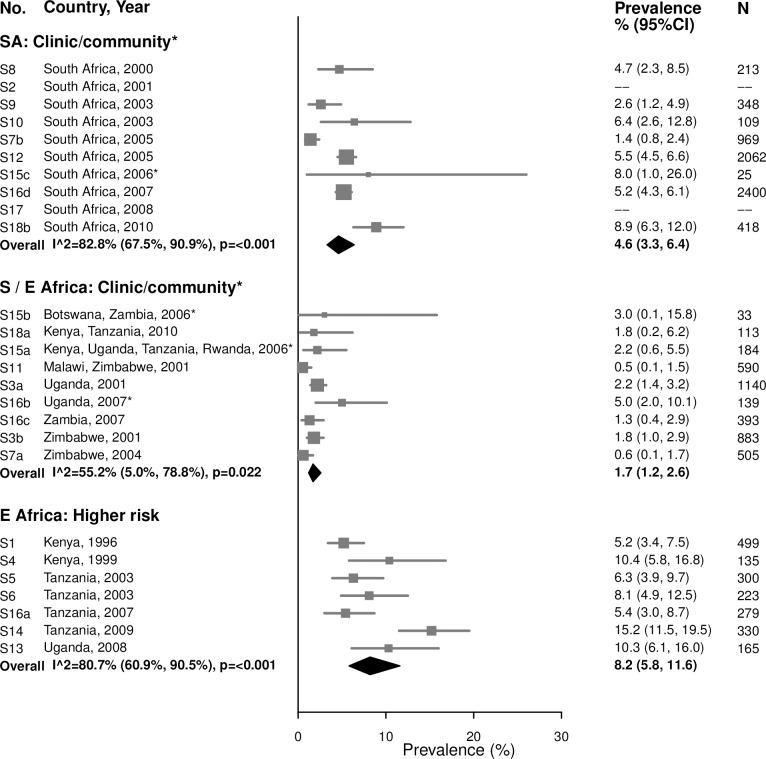
Gonorrhea prevalence among 15–24-year-olds. The area of the marker is proportional to the square root of the sample size. Studies with no estimate presented did not meet the inclusion criteria for this infection and age group. Year presented is the median year that baseline data were collected during the study. *Includes studies of HIV-discordant couples. E Africa, Eastern Africa; SA, South Africa; S/E Africa, Southern and Eastern Africa.

**Fig 4:**
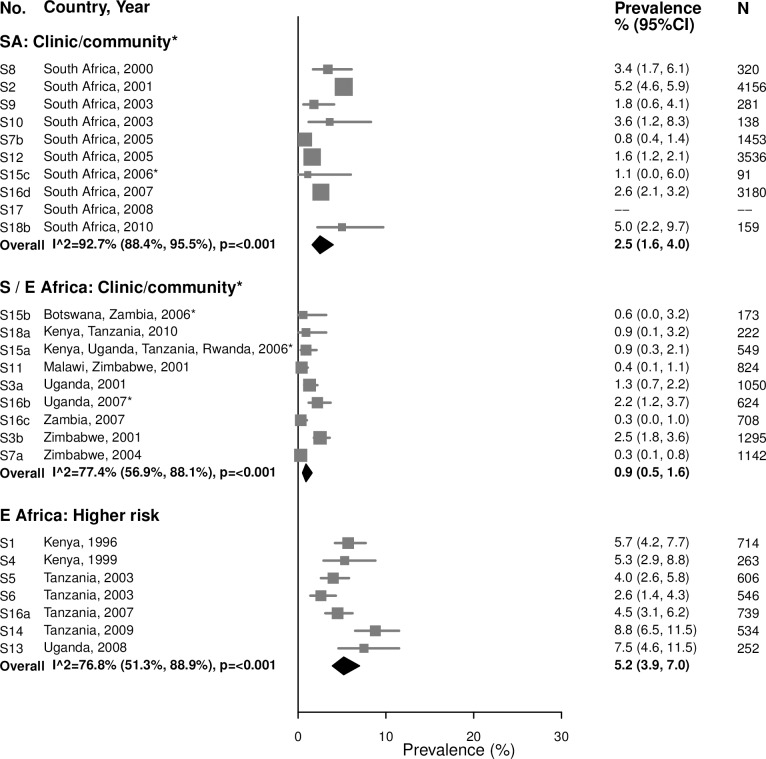
Gonorrhea prevalence among 25–49-year-olds. The area of the marker is proportional to the square root of the sample size. Studies with no estimate presented did not meet the inclusion criteria for this infection and age group. Year presented is the median year that baseline data were collected during the study. *Includes studies of HIV-discordant couples. E Africa, Eastern Africa; SA, South Africa; S/E Africa, Southern and Eastern Africa.

For women aged 15–24 years in clinic/community-based populations in South Africa, estimates of gonorrhea prevalence ranged from 1.4% to 8.9% in individual studies, and the summary estimate was 4.6% (95% CI: 3.3%, 6.4%; *I*^2^ = 82.8%) (Fig 3). The summary estimate of gonorrhea prevalence in clinic/community-based populations in Southern/Eastern Africa was 1.7% (95% CI: 1.2%, 2.6%; *I*^2^ = 55.2%). In the higher-risk populations in Eastern Africa, estimates in individual studies ranged from 5.2% to 15.2%, with a summary estimate of 8.2% (95% CI: 5.8%, 11.6%; *I*^2^ = 80.7%). As for chlamydia, prevalence of gonorrhea among women aged 25–49 years was generally lower than among women aged 15–24 years across all strata, and heterogeneity in all strata was high (Fig 4).

#### Syphilis, overall (high, low, and unknown titer infection)

**Fig 5:**
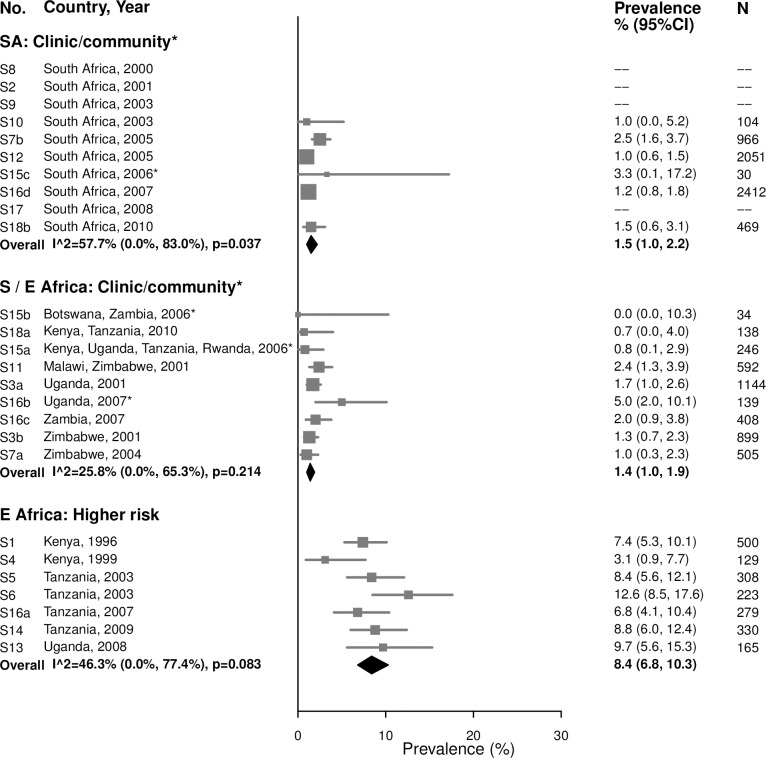
Syphilis overall prevalence among 15–24-year-olds. The area of the marker is proportional to the square root of the sample size. Studies with no estimate presented did not meet the inclusion criteria for this infection and age group. Year presented is the median year that baseline data were collected during the study. *Includes studies of HIV-discordant couples. E Africa, Eastern Africa; SA, South Africa; S/E Africa, Southern and Eastern Africa.

Among 15–24-year-old women, the summary estimate of syphilis prevalence overall (i.e., proportion of participants with a positive RPR and positive treponemal test) was less than 2% in clinic/community populations in both South Africa (1.5% [95% CI: 1.0%, 2.2%]; *I*^2^ = 57.7%) and Southern/Eastern Africa (1.4% [95% CI: 1.0%, 1.9%]; *I*^2^ = 25.8%), and higher among higher-risk populations in Eastern Africa (8.4% [95% CI: 6.8%, 10.3%]; *I*^2^ = 46.3%) (Fig 5).

**Fig 6:**
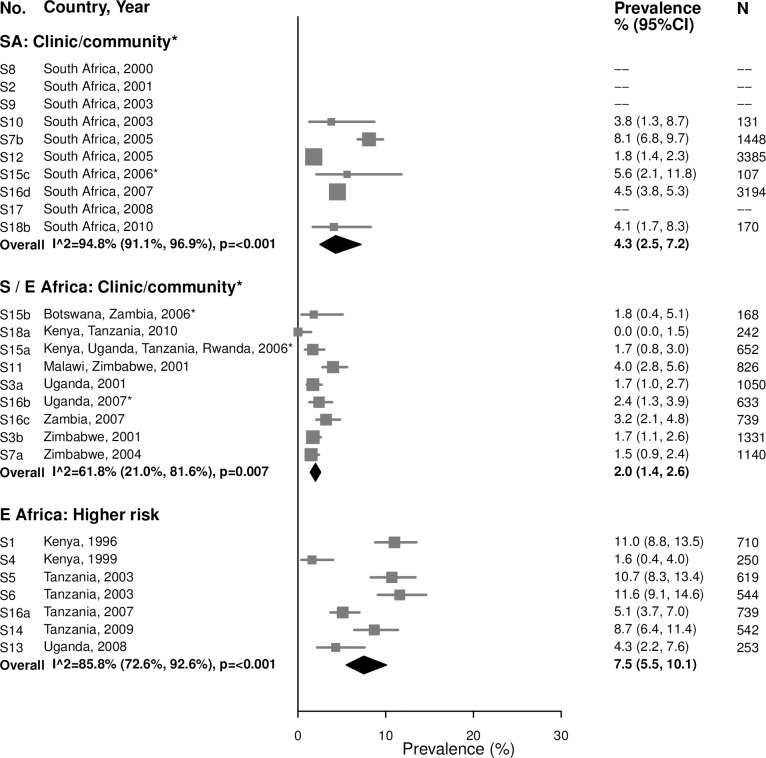
Syphilis overall prevalence among 25–49-year-olds. The area of the marker is proportional to the square root of the sample size. Studies with no estimate presented did not meet the inclusion criteria for this infection and age group. Year presented is the median year that baseline data were collected during the study. *Includes studies of HIV-discordant couples. E Africa, Eastern Africa; SA, South Africa; S/E Africa, Southern and Eastern Africa.

Among 25–49-year-olds in clinic/community populations in South Africa, the summary estimate of syphilis prevalence overall among 25–49-year-old women (4.3% [95% CI: 2.5%, 7.2%]; *I*^2^ = 94.8%) was higher than that among younger women (1.5%) (Fig 6). In clinic/community populations in Southern/Eastern Africa, the summary estimate of syphilis prevalence overall (2.0% [95% CI: 1.4%, 2.6%]; *I*^2^ = 61.8%) was similar to the summary estimate among younger women (1.4%). In the higher-risk populations in Eastern Africa, the summary estimate among older women (7.5% [95% CI: 5.5%, 10.1%]; *I*^2^ = 85.8%) was similar to prevalence among younger women (8.4%).

#### Syphilis, high-titer infection

**Fig 7:**
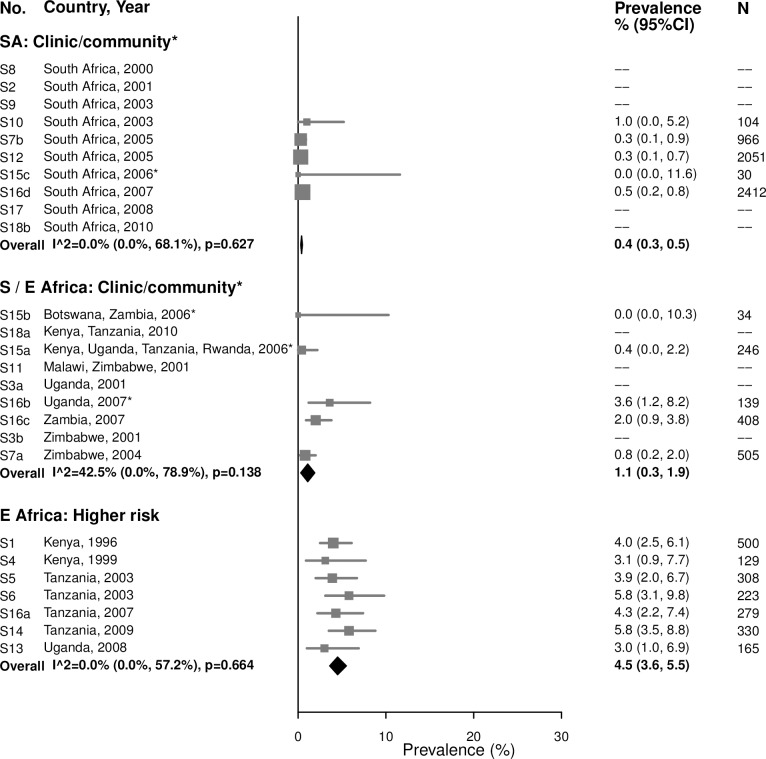
High-titer syphilis prevalence among 15–24-year-olds. The area of the marker is proportional to the square root of the sample size. Studies with no estimate presented did not meet the inclusion criteria for this infection and age group. Year presented is the median year that baseline data were collected during the study. *Includes studies of HIV-discordant couples. E Africa, Eastern Africa; SA, South Africa; S/E Africa, Southern and Eastern Africa.

**Fig 8:**
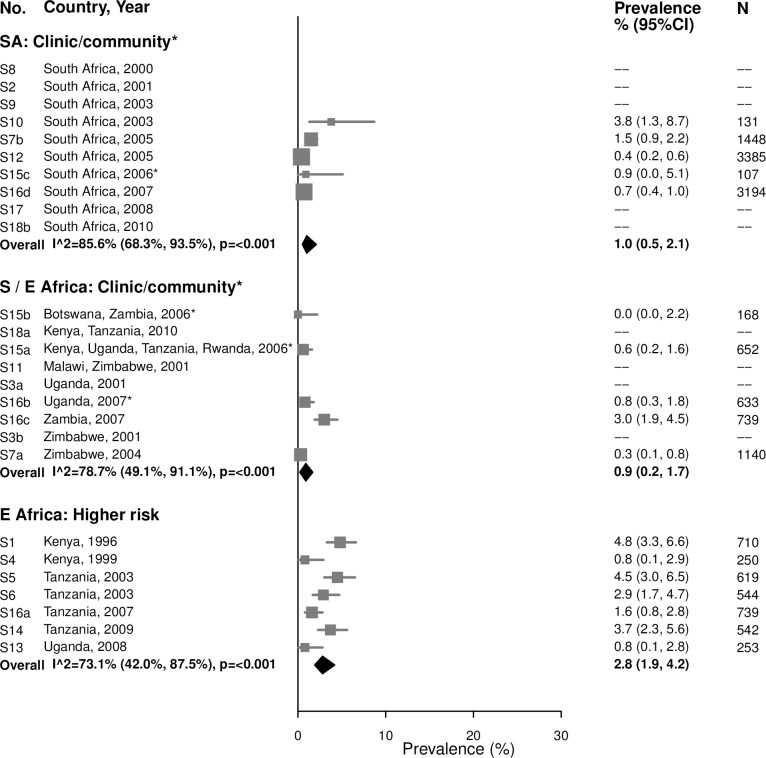
High-titer syphilis prevalence among 25–49-year-olds. The area of the marker is proportional to the square root of the sample size. Studies with no estimate presented did not meet the inclusion criteria for this infection and age group. Year presented is the median year that baseline data were collected during the study. *Includes studies of HIV-discordant couples. E Africa, Eastern Africa; SA, South Africa; S/E Africa, Southern and Eastern Africa.

Among 15–24-year-olds, the prevalence of high-titer, active infectious syphilis (i.e., participants with an RPR titer ≥ 1:8 and a positive treponemal test, among those with RPR titers) mirrored that of overall syphilis, but with low or no between-study heterogeneity (Fig 7). The summary estimate of prevalence in clinic/community populations in South Africa was 0.4% (95% CI: 0.3%, 0.5%; *I*^2^ = 0%) and in Southern/Eastern Africa was 1.1% (95% CI: 0.3%, 1.9%; *I*^2^ = 42.5%). Among higher-risk populations in Eastern Africa, the summary estimate was 4.5% (95% CI: 3.6%, 5.5%; *I*^2^ = 0%). Point estimates for women aged 25–49 years were similar to those in younger populations, but were more heterogeneous (Fig 8).

The 17 studies included in the estimation of high-titer, active infectious syphilis prevalence are a subgroup of the studies included in the estimation of overall syphilis prevalence; summary estimates of overall syphilis prevalence limited to these 17 studies are provided in S9 and S10 Figs.

#### Trichomoniasis

**Fig 9:**
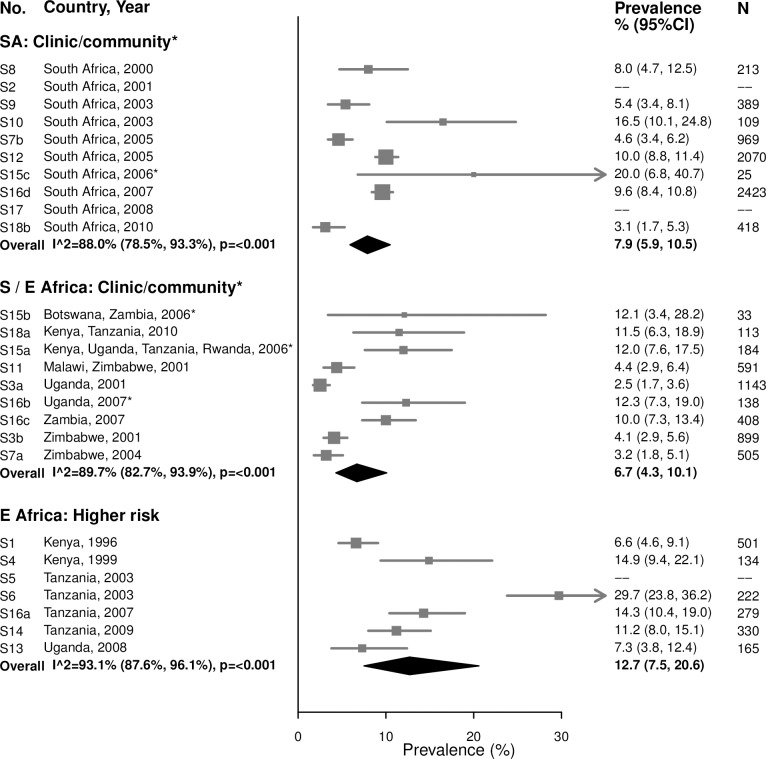
Trichomoniasis prevalence among 15–24-year-olds. The area of the marker is proportional to the square root of the sample size. Studies with no estimate presented did not meet the inclusion criteria for this infection and age group. Year presented is the median year that baseline data were collected during the study. *Includes studies of HIV-discordant couples. E Africa, Eastern Africa; SA, South Africa; S/E Africa, Southern and Eastern Africa.

**Fig 10:**
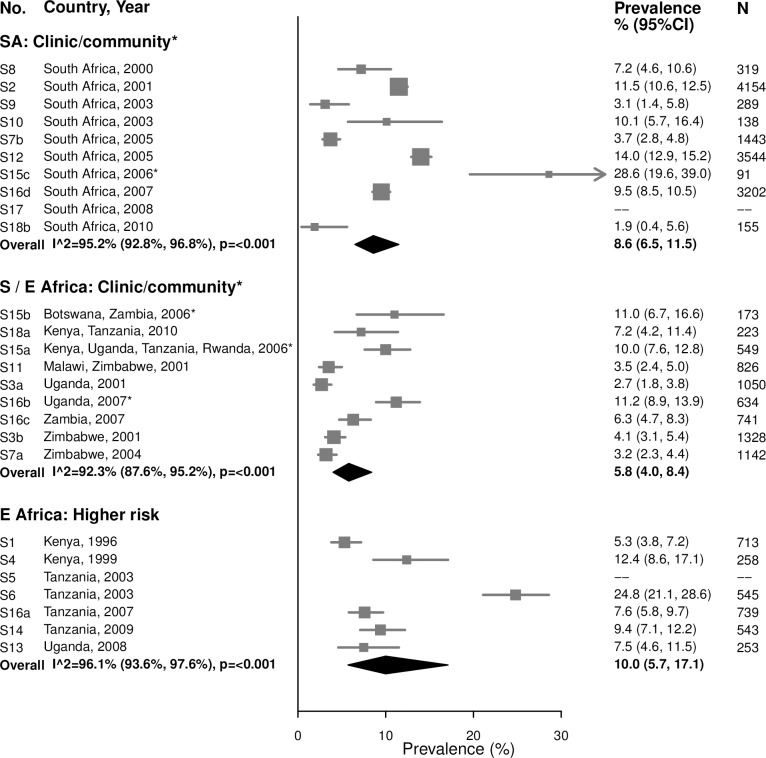
Trichomoniasis prevalence among 25–49-year-olds. The area of the marker is proportional to the square root of the sample size. Studies with no estimate presented did not meet the inclusion criteria for this infection and age group. Year presented is the median year that baseline data were collected during the study. *Includes studies of HIV-discordant couples. E Africa, Eastern Africa; SA, South Africa; S/E Africa, Southern and Eastern Africa.

For both age groups, there was high heterogeneity in estimates of trichomoniasis prevalence across all strata (Figs 9 and 10). For example, among 15–24-year-olds in higher-risk populations in Eastern Africa, estimates ranged from 6.6% to 29.7% (summary estimate: 12.7% [95% CI: 7.5%, 20.6%]; *I*^2^ = 93.1%), and among 25–29-year-olds in the South African clinic/community-based populations, estimates ranged from 1.9% to 28.6% (summary estimate: 8.6% [95% CI: 6.5%, 11.5%]; *I*^2^ = 95.2%).

#### HSV-2

**Fig 11:**
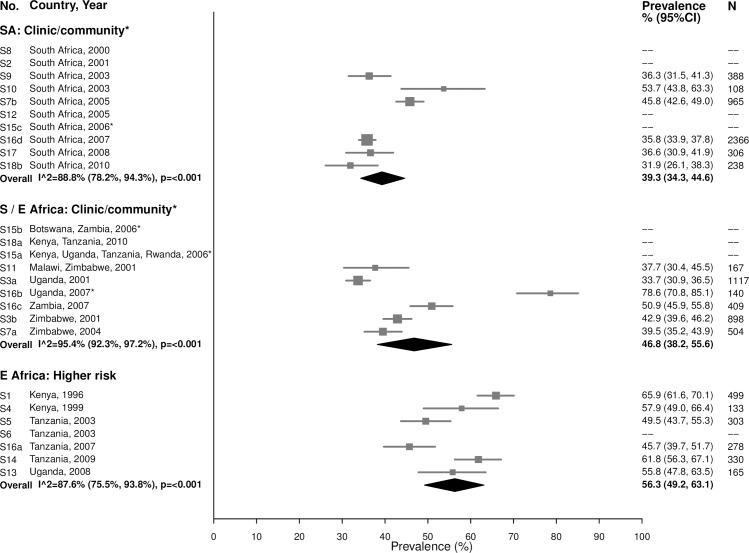
Herpes simplex virus type 2 prevalence among 15–24-year-olds. The area of the marker is proportional to the square root of the sample size. Studies with no estimate presented did not meet the inclusion criteria for this infection and age group. Year presented is the median year that baseline data were collected during the study. *Includes studies of HIV-discordant couples. E Africa, Eastern Africa; SA, South Africa; S/E Africa, Southern and Eastern Africa.

In general, HSV-2 prevalence was high among 15–24-year-olds, ranging from 31.9% to 53.7% in the South African clinic/community-based populations (summary estimate: 39.3% [95% CI: 34.3%, 44.6%]; *I*^2^ = 88.8%), 33.7% to 78.6% in Southern/Eastern African clinic/community-based populations (summary estimate: 46.8% [95% CI: 38.2%, 55.6%]; *I*^2^ = 95.4%), and 45.7% to 65.9% in the higher-risk populations in Eastern Africa (summary estimate: 56.3% [95% CI: 49.2%, 63.1%]; *I*^2^ = 87.6%) (Fig 11).

**Fig 12:**
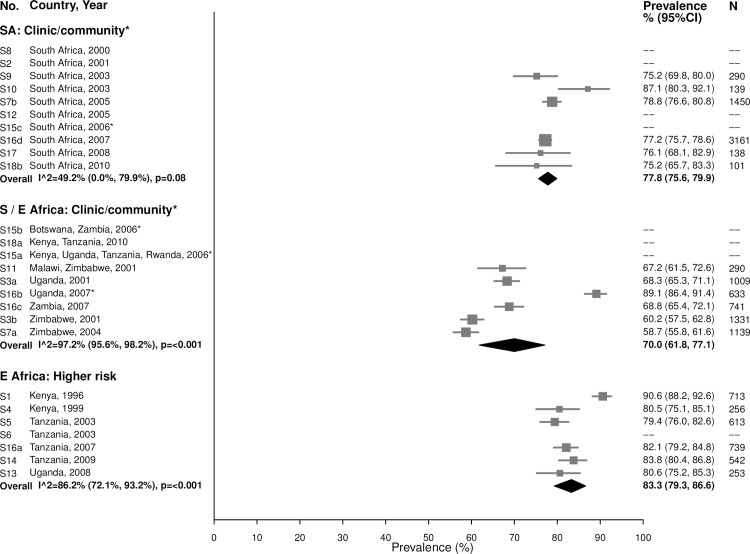
Herpes simplex virus type 2 prevalence among 25–49-year-olds. The area of the marker is proportional to the square root of the sample size. Studies with no estimate presented did not meet the inclusion criteria for this infection and age group. Year presented is the median year that baseline data were collected during the study. *Includes studies of HIV-discordant couples. E Africa, Eastern Africa; SA, South Africa; S/E Africa, Southern and Eastern Africa.

As expected due to the lifelong nature of HSV-2 infection, prevalence of HSV-2 was even higher among women aged 25–49 years (Fig 12). In the South African clinic/community-based populations, the summary estimate was 77.8% (95% CI: 75.6%, 79.9%; *I*^2^ = 49.2%), similar to summary estimates in Southern/Eastern African clinic/community-based populations (70.0% [95% CI: 61.8%, 77.1%]; *I*^2^ = 97.2%) and in the higher-risk populations in Eastern Africa (83.3% [95% CI: 79.3%, 86.6%]; *I*^2^ = 86.2%) (Fig 12).

#### Bacterial vaginosis

**Fig 13:**
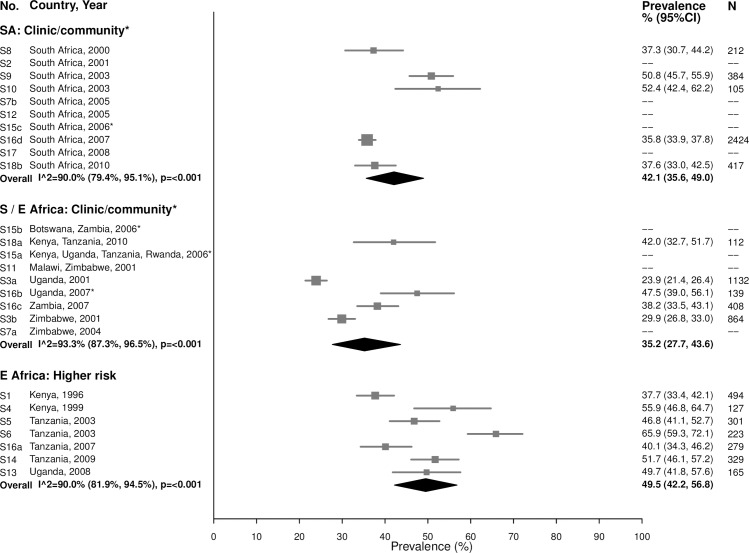
Bacterial vaginosis prevalence among 15–24-year-olds. The area of the marker is proportional to the square root of the sample size. Studies with no estimate presented did not meet the inclusion criteria for this infection and age group. Year presented is the median year that baseline data were collected during the study. *Includes studies of HIV-discordant couples. E Africa, Eastern Africa; SA, South Africa; S/E Africa, Southern and Eastern Africa.

**Fig 14:**
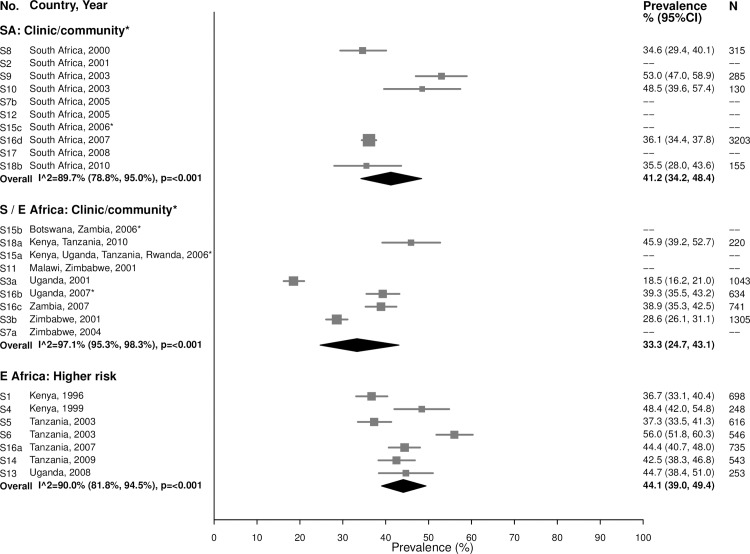
Bacterial vaginosis prevalence among 25–49-year-olds. The area of the marker is proportional to the square root of the sample size. Studies with no estimate presented did not meet the inclusion criteria for this infection and age group. Year presented is the median year that baseline data were collected during the study. *Includes studies of HIV-discordant couples.E Africa, Eastern Africa; SA, South Africa; S/E Africa, Southern and Eastern Africa.

Among 15–24-year-olds in the South African clinic/community-based populations, the summary estimate for BV prevalence was 42.1% (95% CI: 35.6%, 49.0%; *I*^2^ = 90.0%), similar to the summary estimates in Southern/Eastern African clinic/community-based populations (35.2% [95% CI: 27.7%, 43.6%]; *I*^2^ *=* 93.3%) and in the higher-risk populations in Eastern Africa (49.5% [95% CI: 42.2%, 56.8%]; *I*^2^ *=* 90.0%) (Fig 13). Prevalence was similar among women aged 25–49 years, with high heterogeneity across studies (Fig 14).

### Prevalence estimates for co-infection

Patterns of co-infection among women tested for at least 2 infections at baseline varied across STI/BV combinations (Tables 4–9). In general, women with a specific infection had a higher prevalence of another infection than women without this infection. For example, the overall estimated prevalence of chlamydia among young women in South African clinic/community-based populations was 15.1%, but chlamydia prevalence among women infected with another infection ranged from 16.3% (among women with HSV-2) to 38.7% (among women with gonorrhea; Table 4). For some infections, differences were not prominent. For example, the overall prevalence of high-titer, active syphilis among older women in South African clinic/community-based populations did not vary substantially by co-infection status (Table 5). Heterogeneity across studies varied widely by STI/BV combination; overall, 60% of estimates had low heterogeneity, 15% had mild/moderate heterogeneity, and 25% had high heterogeneity.

**Table 4:** Proportion of participants with each infection who are co-infected with an additional STI or BV among women aged 15–24 years in South African clinic/community populations.

Co-infected with	Infected with					
	Chlamydia (*N =* 1,056)	Gonorrhea (*N =* 316)	Syphilis, high-titer infection (*N =* 22)	Trichomoniasis (*N =* 559)	HSV-2 (*N =* 1,675)	BV (*N =* 1,386)
**Chlamydia** **Overall prevalence: 15.1% (12.7, 17.8)**	—	38.7 (28.6, 48.9) *N =* 316 *I*^2^ = 56.1, *P* = 0.026	17.9 (0.0, 36.2) *N =* 22 *I*^2^ = 0, *P* = 0.820	24.2 (19.5, 28.9) *N =* 554 *I*^2^ = 19.7, *P* = 0.274	16.3 (12.7, 19.9) *N =* 1,529 *I*^2^ = 56.4, *P* = 0.043	19.1 (14.8, 23.4) *N =* 1,357 *I*^2^ = 61.7, *P* = 0.023
**Gonorrhea** **Overall prevalence: 4.6% (3.3, 6.4)**	11.4 (7.2, 15.6) *N =* 1,049 *I*^2^ = 67.8, *P* = 0.003	—-	15.3 (0.0, 30.7) *N =* 22 *I*^2^ = 0, *P* = 0.430	9.0 (6.6, 11.4) *N =* 552 *I*^2^ = 0, *P* = 0.617	5.8 (2.3, 9.3) *N =* 1,523 *I*^2^ = 83.6, *P ≤* 0.001	5.7 (3.9, 7.4) *N =* 1,350 *I*^2^ = 30.6, *P* = 0.206
**Syphilis, high-titer infection** **Overall prevalence: 0.4% (0.3, 0.5)**	0.5 (0.0, 1.0) *N =* 878 *I*^2^ = 0, *P* = 1.000	1.3 (0.0, 2.7) *N =* 258 *I*^2^ = 0, *P* = 0.870	—	0.9 (0.0, 1.8) *N =* 499 *I*^2^ = 0, *P* = 0.998	0.5 (0.1, 0.8) *N =* 1,341 *I*^2^ = 0, *P* = 0.666	0.6 (0.1, 1.1) *N =* 946 *I*^2^ = 0, *P* = 0.993
**Trichomoniasis** **Overall prevalence: 7.9% (5.9, 10.5)**	12.2 (9.7, 14.7) *N =* 1,053 *I*^2^ = 20.9, *P* = 0.263	16.2 (12.1, 20.3) *N =* 313 *I*^2^ = 0, *P* = 0.720	25.8 (0.3, 51.2) *N =* 22 *I*^2^ = 33.5, *P* = 0.211	—	8.7 (5.4, 12.0) *N =* 1,547 *I*^2^ = 70.2, *P* = 0.005	9.4 (3.6, 15.1) *N =* 1,378 *I*^2^ = 92.8, *P ≤* 0.001
**HSV-2** **Overall prevalence: 39.3% (34.4, 44.6)**	45.2 (33.2, 57.3) *N =* 600 *I*^2^ = 82.1, *P ≤* 0.001	52.9 (45.4, 60.3) *N =* 175 *I*^2^ = 0, *P* = 0.590	47.8 (5.6, 90.0) *N =* 15 *I*^2^ = 45.2, *P* = 0.161	49.1 (40.1, 58.1) *N =* 315 *I*^2^ = 34.2, *P* = 0.193	—	41.2 (37.4, 45.0) *N =* 1,163 *I*^2^ = 0, *P* = 0.333
**BV** **Overall prevalence: 42.1% (35.6, 49.0)**	42.1 (15.1, 69.1) *N =* 934 *I*^2^ = 98.9, *P ≤* 0.001	38.3 (11.7, 65.0) *N =* 293 *I*^2^ = 96.5, *P ≤* 0.001	38.8 (0.0, 84.6) *N =* 19 *I*^2^ = 70.1, *P* = 0.035	38.3 (11.7, 64.9) *N =* 496 *I*^2^ = 97.8, *P ≤* 0.001	48.2 (37.9, 58.4) *N =* 1,106 *I*^2^ = 83.3, *P ≤* 0.001	—

Data are presented as percent (95% CI) infected with the column infection who are co-infected with the row infection. *N* is the number of participants who were infected with the column infection and were also tested for the row infection; includes data from studies where at least 80% of all participants or at least 80% of a randomly selected sample of enrolled participants were tested for both infections.

BV, bacterial vaginosis; HSV-2, herpes simplex virus type 2; STI, sexually transmitted infection.

**Table 5:** Proportion of participants with each infection who are co-infected with an additional STI or BV among women aged 25–49 years in South African clinic/community populations.

Co-infected with	Infected with					
	Chlamydia (*N =* 796)	Gonorrhea (*N =* 398)	Syphilis, high-titer infection (*N =* 60)	Trichomoniasis (*N =* 1,406)	HSV-2 (*N =* 4,101)	BV (*N =* 1,589)
**Chlamydia** **Overall prevalence: 7.0% (5.5, 8.8)**	—	32.4 (0.0, 69.9) *N =* 398 *I*^2^ = 98.7, *P ≤* 0.001	5.8 (0.0, 11.8) *N =* 60 *I*^2^ = 0, *P* = 0.707	7.1 (4.1, 10.1) *N =* 1,403 *I*^2^ = 60.6, *P* = 0.009	6.5 (4.3, 8.7) *N =* 3,963 *I*^2^ = 76.7, *P ≤* 0.001	8.8 (6.8, 10.8) *N =* 1,568 *I*^2^ = 0, *P* = 0.306
**Gonorrhea** **Overall prevalence: 2.5% (1.6, 4.0)**	18.5 (0.0, 57.5) *N =* 793 *I*^2^ = 99.8, *P ≤* 0.001	—	3.7 (0.0, 11.1) *N =* 60 *I*^2^ = 0, *P* = 0.905	4.0 (1.4, 6.5) *N =* 1,402 *I*^2^ = 64.1, *P* = 0.004	2.2 (0.8, 3.6) *N =* 3,959 *I*^2^ = 80.1, *P ≤* 0.001	2.9 (1.8, 3.9) *N =* 1,562 *I*^2^ = 0, *P* = 0.328
**Syphilis, high-titer infection** **Overall prevalence: 1.0% (0.5, 2.1)**	0.6 (0.0, 1.2) *N =* 475 *I*^2^ = 0, *P* = 0.732	1.1 (0.0, 3.4) *N =* 153 *I*^2^ = 0, *P* = 0.961	—	0.3 (0.0, 0.7) *N =* 848 *I*^2^ = 0, *P* = 0.994	1.3 (0.5, 2.0) *N =* 3,682 *I*^2^ = 34.7, *P* = 0.176	0.6 (0.2, 1.1) *N =* 1,268 *I*^2^ = 0, *P* = 0.795
**Trichomoniasis** **Overall prevalence: 8.6% (6.5, 11.5)**	11.0 (5.0, 17.1) *N =* 792 *I*^2^ = 82.8, *P ≤* 0.001	15.3 (11.7, 18.9) *N =* 396 *I*^2^ = 0, *P* = 0.475	6.5 (0.0, 13.3) *N =* 60 *I*^2^ = 0, *P* = 0.973	—	5.8 (1.6, 9.9) *N =* 3,970 *I*^2^ = 94.8, *P ≤* 0.001	9.5 (5.0, 14.0) *N =* 1,582 *I*^2^ = 82.3, *P ≤* 0.001
**HSV-2** **Overall prevalence: 77.8% (75.6, 79.9)**	81.0 (75.1, 86.9) *N =* 296 *I*^2^ = 21.2, *P* = 0.280	83.2 (75.9, 90.5) *N =* 106 *I*^2^ = 0, *P* = 0.999	90.9 (82.0, 99.8) *N =* 47 *I*^2^ = 0, *P* = 0.915	84.5 (73.5, 95.6) *N =* 368 *I*^2^ = 57.8, *P* = 0.050	—	82.9 (81.0, 84.9) *N =* 1,387 *I*^2^ = 0, *P* = 0.453
**BV** **Overall prevalence: 41.2% (34.2, 48.4)**	38.9 (13.4, 64.5) *N =* 508 *I*^2^ = 97.6, *P ≤* 0.001	30.1 (3.1, 57.1) *N =* 168 *I*^2^ = 92.5, *P ≤* 0.001	26.7 (0.0, 56.8) *N =* 38 *I*^2^ = 70.1, *P* = 0.035	49.5 (16.8, 82.3) *N =* 832 *I*^2^ = 98.8, *P ≤* 0.001	47.5 (37.1, 57.9) *N =* 2,825 *I*^2^ = 90.3, *P ≤* 0.001	—

Data are presented as percent (95% CI) infected with the column infection who are co-infected with the row infection. *N* is the number of participants who were infected with the column infection and were also tested for the row infection; includes data from studies where at least 80% of all participants or at least 80% of a randomly selected sample of enrolled participants were tested for both infections.

BV, bacterial vaginosis; HSV-2, herpes simplex virus type 2; STI, sexually transmitted infection.

**Table 6:** Proportion of participants with each infection who are co-infected with an additional STI or BV among women aged 15–24 years in Southern/Eastern African clinic/community populations.

Co-infected with	Infected with					
	Chlamydia (*N =* 123)	Gonorrhea (*N =* 66)	Syphilis, high-titer infection (*N =* 18)	Trichomoniasis (*N =* 205)	HSV-2 (*N =* 1,341)	BV (*N =* 853)
**Chlamydia** **Overall prevalence: 2.7% (1.5, 3.9)**	—	22.9 (7.8, 37.9) *N =* 66 *I*^2^ = 24.5, *P* = 0.225	12.0 (0.0, 31.5) *N =* 17 *I*^2^ = 0, *P* = 0.904	5.3 (1.0, 9.6) *N =* 201 *I*^2^ = 0, *P* = 0.465	3.6 (1.1, 6.0) *N =* 1,324 *I*^2^ = 85.8, *P ≤* 0.001	3.7 (2.1, 5.4) *N =* 844 *I*^2^ = 33.4, *P* = 0.186
**Gonorrhea** **Overall prevalence: 1.7% (1.2, 2.6)**	13.2 (6.4, 19.9) *N =* 123 *I*^2^ = 0, *P* = 0.537	—	0.0 (0.0, 23.0) *N =* 17 *I*^2^ = 0, *P* = 1.000	4.8 (1.4, 8.2) *N =* 202 *I*^2^ = 0, *P* = 0.724	2.4 (1.3, 3.4) *N =* 1,324 *I*^2^ = 27.2, *P* = 0.231	2.1 (1.1, 3.1) *N =* 844 *I*^2^ = 0, *P* = 0.795
**Syphilis, high-titer infection** **Overall prevalence: 1.1% (0.3, 1.9)**	6.5 (0.0, 18.4) *N =* 28 *I*^2^ = 0, *P* = 0.997	0.0 (0.0, 21.7) *N =* 19 *I*^2^ = 0, *P* = 1.000	—	2.6 (0.0, 6.7) *N =* 98 *I*^2^ = 0, *P* = 1.000	1.7 (0.6, 2.9) *N =* 516 *I*^2^ = 0, *P* = 0.672	1.8 (0.0, 3.5) *N =* 222 *I*^2^ = 0, *P* = 1.000
**Trichomoniasis** **Overall prevalence: 6.7% (4.3, 10.1)**	10.3 (4.2, 16.5) *N =* 123 *I*^2^ = 0, *P* = 0.938	17.3 (7.0, 27.6) *N =* 66 *I*^2^ = 0, *P* = 0.878	12.4 (0.0, 31.5) *N =* 17 *I*^2^ = 0, *P* = 0.942	—	6.9 (3.9, 9.8) *N =* 1,339 *I*^2^ = 79.1, *P ≤* 0.001	8.9 (4.8, 13.0) *N =* 852 *I*^2^ = 78.4, *P ≤* 0.001
**HSV-2** **Overall prevalence: 46.8% (38.2, 55.6)**	56.5 (47.0, 65.9) *N =* 109 *I*^2^ = 0, *P* = 0.736	62.7 (49.3, 76.0) *N =* 56 *I*^2^ = 0, *P* = 0.500	72.6 (49.9, 95.4) *N =* 17 *I*^2^ = 0, *P* = 0.680	65.3 (47.3, 83.3) *N =* 144 *I*^2^ = 83.4, *P ≤* 0.001	—	56.9 (39.3, 74.6) *N =* 769 *I*^2^ = 95.1, *P ≤* 0.001
**BV** **Overall prevalence: 35.2% (27.7, 43.6)**	39.9 (23.8, 55.9) *N =* 100 *I*^2^ = 45.4, *P* = 0.103	30.9 (18.8, 42.9) *N =* 58 *I*^2^ = 0, *P* = 0.470	29.5 (3.1, 56.0) *N =* 13 *I*^2^ = 0, *P* = 0.519	45.8 (26.4, 65.1) *N =* 156 *I*^2^ = 86.0, *P ≤* 0.001	35.1 (27.3, 43.0) *N =* 1,120 *I*^2^ = 86.0, *P ≤* 0.001	—

Data are presented as percent (95% CI) infected with the column infection who are co-infected with the row infection. *N* is the number of participants who were infected with the column infection and were also tested for the row infection; includes data from studies where at least 80% of all participants or at least 80% of a randomly selected sample of enrolled participants were tested for both infections.

BV, bacterial vaginosis; HSV-2, herpes simplex virus type 2; STI, sexually transmitted infection.

**Table 7:** Proportion of participants with each infection who are co-infected with an additional STI or BV among women aged 25–49 years in Southern/Eastern African clinic/community populations.

Co-infected with	Infected with					
	Chlamydia (*N =* 82)	Gonorrhea (*N =* 77)	Syphilis, high-titer infection (*N =* 34)	Trichomoniasis (*N =* 357)	HSV-2 (*N =* 3,458)	BV (*N =* 1,297)
**Chlamydia** **Overall prevalence: 1.2% (0.8, 1.7)**	—	9.8 (0.5, 19.2) *N =* 77 *I*^2^ = 0, *P* = 0.770	0.0 (0.0, 13.8) *N =* 31 *I*^2^ = 0, *P* = 1.000	2.1 (0.3, 3.9) *N =* 354 *I*^2^ = 0, *P* = 0.984	1.0 (0.3, 1.6) *N =* 3,365 *I*^2^ = 76.8, *P ≤* 0.001	1.3 (0.4, 2.3) *N =* 1,273 *I*^2^ = 59.5, *P* = 0.030
**Gonorrhea** **Overall prevalence: 0.9% (0.5, 1.6)**	10.0 (0.9, 19.1) *N =* 82 *I*^2^ = 0, *P* = 0.806	—	2.4 (0.0, 16.1) *N =* 31 *I*^2^ = 0, *P* = 0.830	2.2 (0.3, 4.1) *N =* 354 *I*^2^ = 0, *P* = 0.843	1.5 (0.6, 2.4) *N =* 3,366 *I*^2^ = 84.9, *P ≤* 0.001	1.8 (0.5, 3.1) *N =* 1,273 *I*^2^ = 69.9, *P* = 0.005
**Syphilis, high-titer infection** **Overall prevalence: 0.9% (0.2, 1.7)**	0.0 (0.0, 17.2) *N =* 27 *I*^2^ = 0, *P* = 1.000	5.7 (0.0, 18.3) *N =* 25 *I*^2^ = 0, *P* = 1.000	—	1.7 (0.0, 4.0) *N =* 224 *I*^2^ = 0, *P* = 0.976	1.3 (0.2, 2.4) *N =* 1,740 *I*^2^ = 61.6, *P* = 0.023	1.3 (0.3, 2.3) *N =* 534 *I*^2^ = 0, *P* = 0.684
**Trichomoniasis** **Overall prevalence: 5.8% (4.0, 8.4)**	5.4 (0.2, 10.5) *N =* 82 *I*^2^ = 0, *P* = 0.931	10.1 (2.4, 17.9) *N =* 77 *I*^2^ = 0, *P* = 0.436	13.1 (0.0, 26.1) *N =* 32 *I*^2^ = 0, *P* = 0.922	—	6.0 (3.6, 8.3) *N =* 3,425 *I*^2^ = 89.4, *P ≤* 0.001	8.6 (4.6, 12.5) *N =* 1,297 *I*^2^ = 86.7, p *P ≤* 0.001
**HSV-2** **Overall prevalence: 70.0% (61.8, 77.1)**	59.5 (47.6, 71.4) *N =* 68 *I*^2^ = 0, *P* = 0.652	84.3 (75.2, 93.5) *N =* 69 *I*^2^ = 0, *P* = 0.956	85.2 (70.4, 100.0) *N =* 30 *I*^2^ = 0, *P* = 0.646	84.9 (75.2, 94.6) *N =* 245 *I*^2^ = 80.8, *P ≤* 0.001	—	75.1 (62.5, 87.8) *N =* 1,143 *I*^2^ = 95.4, *P ≤* 0.001
**BV** **Overall prevalence: 33.3% (24.7, 43.1)**	39.3 (20.2, 58.4) *N =* 60 *I*^2^ = 46.5, *P* = 0.096	45.9 (33.6, 58.2) *N =* 66 *I*^2^ = 0, *P* = 0.734	36.9 (18.0, 55.8) *N =* 27 *I*^2^ = 0, *P* = 0.891	45.2 (28.7, 61.7) *N =* 241 *I*^2^ = 86.3, *P ≤* 0.001	29.9 (21.3, 38.5) *N =* 2,736 *I*^2^ = 96.0, *P ≤* 0.001	—

Data are presented as percent (95% CI) infected with the column infection who are co-infected with the row infection. *N* is the number of participants who were infected with the column infection and were also tested for the row infection; includes data from studies where at least 80% of all participants or at least 80% of a randomly selected sample of enrolled participants were tested for both infections.

BV, bacterial vaginosis; HSV-2, herpes simplex virus type 2; STI, sexually transmitted infection.

**Table 8:** Proportion of participants with each infection who are co-infected with an additional STI or BV among women aged 15–24 years in Eastern African higher-risk populations.

Co-infected with	Infected with					
	Chlamydia (*N =* 147)	Gonorrhea (*N =* 159)	Syphilis, high-titer infection (*N =* 85)	Trichomoniasis (*N =* 208)	HSV-2 (*N =* 979)	BV (*N =* 909)
**Chlamydia** **Overall prevalence: 10.3% (7.4, 14.1)**	—	19.1 (9.9, 28.4) *N =* 156 *I*^2^ = 47.6, *P* = 0.076	6.9 (0.7, 13.1) *N =* 85 *I*^2^ = 0, *P* = 0.968	7.0 (3.5, 10.5) *N =* 205 *I*^2^ = 0, *P* = 0.516	9.3 (5.0, 13.6) *N =* 913 *I*^2^ = 82.1, *P ≤* 0.001	10.4 (6.6, 14.3) *N =* 867 *I*^2^ = 74.4, *P ≤* 0.001
**Gonorrhea** **Overall prevalence: 8.2% (5.8, 11.6)**	20.2 (12.7, 27.7) *N =* 147 *I*^2^ = 23.4, *P* = 0.258	—	9.2 (2.6, 15.7) *N =* 85 *I*^2^ = 0, *P* = 0.700	10.9 (4.2, 17.6) *N =* 208 *I*^2^ = 61.8, *P* = 0.023	11.5 (7.6, 15.3) *N =* 972 *I*^2^ = 71.3, *P* = 0.004	9.6 (6.3, 12.9) *N =* 909 *I*^2^ = 67.6, *P* = 0.005
**Syphilis, high-titer infection** **Overall prevalence: 4.5% (3.6, 5.5)**	4.1 (0.1, 8.0) *N =* 147 *I*^2^ = 0, *P* = 0.968	5.1 (1.3, 8.9) *N =* 158 *I*^2^ = 0, *P* = 0.679	—	9.0 (5.1, 12.9) *N =* 207 *I*^2^ = 0, *P* = 0.510	5.4 (4.0, 6.8) *N =* 972 *I*^2^ = 0, *P* = 0.735	5.5 (4.0, 7.0) *N =* 904 *I*^2^ = 0, *P* = 0.859
**Trichomoniasis** **Overall prevalence: 12.7% (7.5, 20.6)**	11.3 (3.8, 18.8) *N =* 131 *I*^2^ = 46.3, *P* = 0.114	18.2 (7.4, 29.0) *N =* 140 *I*^2^ = 68.4, *P* = 0.007	29.8 (18.9, 40.7) *N =* 73 *I*^2^ = 0, *P* = 0.976	—	12.7 (7.1, 18.2) *N =* 827 *I*^2^ = 79.6, *P ≤* 0.001	15.6 (8.7, 22.4) *N =* 768 *I*^2^ = 86.2, *P ≤* 0.001
**HSV-2** **Overall prevalence: 56.3% (49.2, 63.1)**	59.2 (50.4, 68.1) *N =* 122 *I*^2^ = 0, *P* = 0.780	79.5 (72.8, 86.2) *N =* 141 *I*^2^ = 0, *P* = 0.682	78.4 (68.5, 88.3) *N =* 71 *I*^2^ = 0, *P* = 0.941	66.5 (58.6, 74.3) *N =* 142 *I*^2^ = 0, *P* = 0.841	—	66.8 (48.5, 85.0) *N =* 905 *I*^2^ = 98.0, *P ≤* 0.001
**BV** **Overall prevalence: 49.5% (42.2, 56.8)**	58.2 (50.2, 66.2) *N =* 147 *I*^2^ = 0, *P* = 0.440	57.3 (46.9, 67.6) *N =* 159 *I*^2^ = 41.7, *P* = 0.113	64.2 (52.0, 76.4) *N =* 85 *I*^2^ = 27.2, *P* = 0.221	58.4 (43.3, 73.4) *N =* 207 *I*^2^ = 81.1, *P ≤* 0.001	50.8 (44.2, 57.4) *N =* 963 *I*^2^ = 75.3, *P* = 0.001	—

Data are presented as percent (95% CI) infected with the column infection who are co-infected with the row infection. *N* is the number of participants who were infected with the column infection and were also tested for the row infection; includes data from studies where at least 80% of all participants or at least 80% of a randomly selected sample of enrolled participants were tested for both infections.

BV, bacterial vaginosis; HSV-2, herpes simplex virus type 2; STI, sexually transmitted infection.

**Table 9:** Proportion of participants with each infection who are co-infected with an additional STI or BV among women aged 25–49 years in Eastern African higher-risk populations.

Co-infected with	Infected with					
	Chlamydia (*N =* 184)	Gonorrhea (*N =* 192)	Syphilis, high-titer infection (*N =* 114)	Trichomoniasis (*N =* 331)	HSV-2 (*N =* 2,604)	BV (*N =* 1,582)
**Chlamydia** **Overall prevalence: 6.2% (4.7, 8.2)**	—	9.6 (5.4, 13.8) *N =* 189 *I*^2^ = 0, *P* = 0.541	8.2 (2.1, 14.3) *N =* 107 *I*^2^ = 0, *P* = 0.492	7.0 (4.2, 9.8) *N =* 323 *I*^2^ = 0, *P* = 0.730	5.4 (3.6, 7.3) *N =* 2,411 *I*^2^ = 76.2, *P ≤* 0.001	5.9 (4.0, 7.8) *N =* 1,510 *I*^2^ = 60.6, *P* = 0.019
**Gonorrhea** **Overall prevalence: 5.2% (3.9, 7.0)**	9.3 (5.1, 13.6) *N =* 184 *I*^2^ = 0, *P* = 0.628	—	5.7 (1.3, 10.1) *N =* 113 *I*^2^ = 0, *P* = 0.991	5.8 (3.2, 8.5) *N =* 331 *I*^2^ = 0, *P* = 0.853	5.9 (4.8, 7.1) *N =* 2,583 *I*^2^ = 36.8, *P* = 0.161	6.1 (3.9, 8.4) *N =* 1,573 *I*^2^ = 74.2, *P ≤* 0.001
**Syphilis, high-titer infection** **Overall prevalence: 2.8% (1.9, 4.2)**	4.5 (1.1, 8.0) *N =* 183 *I*^2^ = 0, *P* = 0.722	4.0 (0.9, 7.0) *N =* 192 *I*^2^ = 0, *P* = 0.994	—	4.0 (1.7, 6.4) *N =* 329 *I*^2^ = 0, *P* = 0.706	2.9 (1.3, 4.5) *N =* 2,585 *I*^2^ = 87.6, *P ≤* 0.001	3.1 (1.8, 4.5) *N =* 1,569 *I*^2^ = 61.8, *P* = 0.015
**Trichomoniasis** **Overall prevalence: 10.0% (5.7, 17.1)**	14.2 (5.9, 22.5) *N =* 153 *I*^2^ = 53.7, *P* = 0.071	11.1 (3.0, 19.2) *N =* 168 *I*^2^ = 60.3, *P* = 0.028	16.3 (1.8, 30.7) *N =* 85 *I*^2^ = 58.2, *P* = 0.035	—	8.4 (6.2, 10.6) *N =* 2,109 *I*^2^ = 65.0, *P* = 0.014	14.2 (8.5, 19.8) *N =* 1,346 *I*^2^ = 88.0, *P ≤* 0.001
**HSV-2** **Overall prevalence: 83.3% (79.3, 86.6)**	83.1 (76.2, 90.0) *N =* 148 *I*^2^ = 21.3, *P* = 0.279	89.9 (85.1, 94.6) *N =* 178 *I*^2^ = 0, *P* = 0.342	96.2 (92.0, 100.0) *N =* 98 *I*^2^ = 0, *P* = 0.868	89.3 (83.0, 95.7) *N =* 194 *I*^2^ = 50.0, *P* = 0.091	—	88.6 (82.6, 94.6) *N =* 1,577 *I*^2^ = 95.6, *P ≤* 0.001
**BV** **Overall prevalence: 44.1% (39.0, 49.4)**	47.9 (37.1, 58.7) *N =* 182 *I*^2^ = 53.3, *P* = 0.058	51.6 (42.1, 61.1) *N =* 189 *I*^2^ = 41.3, *P* = 0.116	51.1 (41.8, 60.5) *N =* 112 *I*^2^ = 0, *P* = 0.489	59.9 (53.4, 66.5) *N =* 329 *I*^2^ = 27.4, *P* = 0.229	43.7 (40.3, 47.1) *N =* 2,569 *I*^2^ = 66.6, *P* = 0.011	—

Data are presented as percent (95% CI) infected with the column infection who are co-infected with the row infection. *N* is the number of participants who were infected with the column infection and were also tested for the row infection; includes data from studies where at least 80% of all participants or at least 80% of a randomly selected sample of enrolled participants were tested for both infections.

BV, bacterial vaginosis; HSV-2, herpes simplex virus type 2; STI, sexually transmitted infection.

Some of the most striking findings were related to chlamydia/gonorrhea co-infection. Among women with chlamydia at baseline, the estimated prevalence of co-infection with gonorrhea was about 10%–20%. Among women with gonorrhea at baseline, estimated prevalence of co-infection with chlamydia varied by population type and age group: estimated co-infection prevalence was about 30%–40% among younger and older women in South African clinic/community-based populations (Tables 4 and 5) and was lower (about 10%) among older women in Southern/Eastern African clinic/community-based populations (Table 7) and older women in higher-risk populations in Eastern Africa (Table 9).

## Discussion

In this IPD meta-analysis, we combined data from 18 prospective HIV prevention studies representing over 37,000 women in sub-Saharan Africa to estimate prevalence of chlamydia, gonorrhea, syphilis, trichomoniasis, HSV-2, and BV. We observed age-, region- and population-specific patterns in the prevalence of these infections, including higher prevalence of STIs among younger (15–24-year-old) than older (25–49-year-old) women, generally higher STI prevalence in clinic/community-based populations in South Africa than among similar populations elsewhere in Southern/Eastern Africa, and notably greater prevalence among higher-risk populations for certain STIs (e.g., gonorrhea and syphilis), but not for others (e.g., HSV-2 and BV, which had high prevalence across all population types).

A major strength of this analysis is that it provides important information on STI and BV prevalence that would not otherwise be readily available through literature searches of published data. Few of our included studies had published detailed results about STI or BV. None of the data from the included studies were identified through the literature search process used to generate the 2008 and 2012 WHO global curable STI estimates [1,37], because they included only higher-risk populations, they were published outside the time frames of the literature searches, they were not identified in the searches, or the published papers did not provide enough information (e.g., estimates not stratified by age group, sex, or country/region) to include in the WHO estimates. One study that initially did not have enough STI information in the published paper to include in the WHO 2012 estimates was later identified during this STI IPD meta-analysis and subsequently incorporated [30,31]. Combining STI/BV data from a large number of studies allowed us to estimate prevalence by age and population type more precisely for distinct regions within sub-Saharan Africa.

While this IPD meta-analysis provides a unique opportunity to strengthen understanding of the epidemiology of STIs/BV among sub-Saharan African women, several limitations need to be considered. First, the studies included in the HC-HIV meta-analysis dataset were not specifically selected to assess STI/BV prevalence, and should be considered a convenience sample of studies. There may have been additional studies from the region that did not meet inclusion criteria for the HC-HIV meta-analysis, but contained STI/BV data, including data for men. In addition, the women in these studies were not randomly sampled from the populations they represent. Women enrolled from clinical settings in particular may be more likely to be symptomatic or recently exposed to an STI, which would tend to overestimate STI prevalence. Some studies only enrolled women in clinic/community settings who reported recent sexual activity or more than 1 partner in the recent past [33]. Conversely, because all included studies were prospective evaluations of HIV acquisition, women included in this baseline evaluation were all HIV-negative. Thus, we may have underestimated STI/BV prevalence in communities with high HIV prevalence (such as in South Africa), where HIV-negative women may be at lower STI/HIV risk than others in their communities. Reassuringly, the prevalences of curable STIs we observed in clinic/community-based populations, especially in Southern/Eastern Africa, were similar to WHO 2012 estimates for African women (which were limited to general populations) [1]. Another limitation of this meta-analysis is that it included some studies that are more than 15 years old. Summary estimates could be biased if prevalence has changed over time. Evidence of temporal trends in the prevalence of STIs/BV in LMICs is limited, in part due to lack of standardized surveillance systems and differing methodology for repeated sets of regional estimates [1]. Available data from a few large studies have not shown clear evidence of changing epidemiology over the last two decades, beyond methodological issues in the estimation process [1–3]. Aside from syphilis—for which available rapid diagnostic tests and concerted efforts to screen and treat pregnant women to avert mother-to-child transmission have resulted in likely declines over time [1,38]—the other STIs and BV lack clearly effective population-level interventions in these settings. This provides confidence that even though some studies in this IPD meta-analysis are older, the data provide a general idea of the overall STI epidemic in these regions. Future research could combine these data with additional data points to further the understanding of global trends over time.

We observed high heterogeneity across studies when calculating summary estimates for STI/BV prevalence in some populations. Observed heterogeneity can reflect differences in study design, setting, sample size, and recruitment strategies between studies. We present summary estimates irrespective of the degree of between-study heterogeneity to facilitate the comparison of overall patterns between the population groups for each infection; however, observed heterogeneity should be taken into account when interpreting findings. For example, differences in test technology might explain some of the heterogeneity for some infections. We attempted to minimize this by excluding studies with less accurate tests for a particular STI or BV (e.g., studies that diagnosed BV based only on Amsel clinical criteria [39]). For some STIs, however, results from diagnostic tests with varying levels of sensitivity and specificity were included. Some of the most noticeable heterogeneity was observed among trichomoniasis estimates, for which a large number of studies used only wet mount, which is notably insensitive [40]. For HSV-2, although all included studies used EIAs, some studies used EIAs with lower specificity [41]. For chlamydia and gonorrhea, prevalence estimates were relatively consistent across studies despite some high *I*^2^ values. For syphilis estimates, heterogeneity was less pronounced. To refine prevalence estimates, future work could consider additional investigation into heterogeneity by diagnostic test type. Finally, studies that were conducted in multiple regions or among multiple population types were split into sub-studies; it is possible that estimates from these sub-studies are not independent.

We observed several patterns that highlight important features of the STI/BV epidemics in sub-Saharan Africa, which address the first strategic direction of the Global Strategy for STIs: “information for focused action: the who and the where.” In terms of the “who,” across all populations and regions, STI prevalence was generally higher among younger than older women, except for the prevalence of antibodies to HSV-2, which increased with age. Higher STI prevalence among young people has been observed worldwide and highlights the need for global efforts to improve sexual and reproductive health in adolescent girls and young women [42–44]. Additionally, we found that women who were in HIV-discordant partnerships had similar STI prevalence as women recruited from clinic/community-based populations, perhaps reflecting that although these participants may be at higher risk for HIV transmission than the general population, their risk for STIs/BV may be similar to that of clinic/community-based populations, given that they are in stable relationships.

As for the “where,” we found that women enrolled from clinic or community-based settings in South Africa had particularly high prevalence of STIs. In many cases, STI prevalence in South African women was similar to or greater than that of populations in Eastern Africa that were recruited from settings thought to be higher risk for HIV, such as women who report transactional sex. These findings are consistent with other studies in South Africa showing high prevalence of infections like chlamydia and gonorrhea [45,46]. This is particularly concerning given the likely increased risk of HIV acquisition posed by these inflammatory STIs [47,48]. Because of variability in how studies measured sexual behavior, we were unable to fully explore reasons for differences in STI prevalence across regions. South African women in the included studies were, however, less likely to be married or living with a partner compared with Southern/Eastern African women, although they were more likely than other groups to report condom use at last sex [49,50]. Additional work, such as research that incorporates anthropological and other qualitative methodologies, could help elucidate the cultural and behavioral drivers of observed differences between these populations.

The estimated prevalence of chlamydia in clinic/community populations in Southern/Eastern Africa was very similar to estimates from high-income countries in Europe and in the United States, including countries with well-established chlamydia control programs [51,52]. HSV-2 and BV prevalence was relatively high across all regions and population types. Gonorrhea and syphilis, on the other hand, were primarily concentrated among women recruited from settings thought to be at higher risk for HIV, which in this study were all in Eastern Africa. This suggests that targeting interventions to both populations and geographic regions may be important in addressing 2 of the 3 main areas of intervention focus for the Global Strategy: first, controlling the spread and impact of gonorrhea, particularly to stem the threat of gonococcal antimicrobial resistance [53], and, second, dramatically reducing mother-to-child transmission of syphilis and HIV [54]. The third main area of intervention focus of the Global Strategy is fully utilizing HPV vaccines; however, too few studies in the HC-HIV dataset collected information on HPV infection for us to evaluate this important STI.

### Implications

Innovative approaches to obtaining better data on the STI burden are critical for developing and implementing prevention interventions, and monitoring evidence of program impact. Global STI management guidelines are currently being updated [55], and key decisions about the use of syndromic management (the use of presumptive treatment for symptomatic people without the use of laboratory tests) are dependent on accurate STI and BV prevalence data in different settings [56]. Our assessment of co-infections among the STIs can also help guide decision-making related to treatment. These prevalence data can also provide critical inputs to guide decision-making about STI prevention and control programs in different settings, e.g., congenital syphilis elimination efforts, the role of STI laboratory testing and treatment, including screening, and potential cost-effectiveness.

STI prevalence data also allow more accurate assessment of the need and potential impact of future interventions. A global roadmap for accelerating development of new STI vaccines outlined how better data on STI burden in LMICs are critical for assessing more accurately the predicted impact of STI vaccines, developing investment cases for vaccine development, and guiding future vaccine implementation and evaluation [57,58]. Work on a vaccine against HSV is well underway [59], which is crucial given the HSV-2 prevalence of roughly 70%–80% among 25–49-year-old women in this analysis and the notable increased risk of HIV due to HSV [60]. Proof-of-principle findings from a study of how the meningococcal B vaccine might impact gonorrhea are also intriguing [61]. Given augmentation of HIV risk with all STIs as well as BV, and other major reproductive health outcomes such as infertility, not only STI vaccines but multipurpose prevention technologies such as vaginal microbicides and the role of expanded screening through point-of-care tests should also be explored [60,62].

Collecting better information on STI prevalence and incidence is the first key strategic direction of the new Global Strategy [4]. Given resource and logistical constraints, de novo dedicated STI prevalence studies may not be realistic in many settings, particularly in sub-Saharan Africa, where the impact of STIs and their consequences may be greatest [63]. This IPD meta-analysis leverages existing STI data to fill this unmet need and offers a new approach to obtaining critically needed information on the epidemiology of STIs in LMICs. In addition to seeking opportunities to add STI laboratory testing to surveillance studies and clinical trials from the outset, this methodology can be applied to other regions and populations, including men, to further inform global estimates of STI and BV burden.

## Supporting information

S1 Fig Chlamydia prevalence among 15–24-year-olds, stratified by initial region and population type category. E Africa, Eastern Africa; SA, South Africa; S Africa, Southern Africa. The area of the marker is proportional to the square root of the sample size. Studies with no estimate presented did not meet the inclusion criteria for this infection and age group. Year presented is the median year that baseline data were collected during the study.
(TIF)

S2 Fig Chlamydia prevalence among 25–49-year-olds, stratified by initial region and population type category. E Africa, Eastern Africa; SA, South Africa; S Africa, Southern Africa. The area of the marker is proportional to the square root of the sample size. Studies with no estimate presented did not meet the inclusion criteria for this infection and age group. Year presented is the median year that baseline data were collected during the study.
(TIF)

S3 Fig Gonorrhea prevalence among 15–24-year-olds, stratified by initial region and population type category. E Africa, Eastern Africa; SA, South Africa; S Africa, Southern Africa. The area of the marker is proportional to the square root of the sample size. Studies with no estimate presented did not meet the inclusion criteria for this infection and age group. Year presented is the median year that baseline data were collected during the study.
(TIF)

S4 Fig Gonorrhea prevalence among 25–49-year-olds, stratified by initial region and population type category. E Africa, Eastern Africa; SA, South Africa; S Africa, Southern Africa. The area of the marker is proportional to the square root of the sample size. Studies with no estimate presented did not meet the inclusion criteria for this infection and age group. Year presented is the median year that baseline data were collected during the study.
(TIF)

S5 Fig Syphilis overall prevalence among 15–24-year-olds, stratified by initial region and population type category. E Africa, Eastern Africa; SA, South Africa; S Africa, Southern Africa. The area of the marker is proportional to the square root of the sample size. Studies with no estimate presented did not meet the inclusion criteria for this infection and age group. Year presented is the median year that baseline data were collected during the study.
(TIF)

S6 Fig Syphilis overall prevalence among 25–49-year-olds, stratified by initial region and population type category. E Africa, Eastern Africa; SA, South Africa; S Africa, Southern Africa. The area of the marker is proportional to the square root of the sample size. Studies with no estimate presented did not meet the inclusion criteria for this infection and age group. Year presented is the median year that baseline data were collected during the study.
(TIF)

S7 Fig High-titer, active syphilis prevalence among 15–24-year-olds, stratified by initial region and population type category. E Africa, Eastern Africa; SA, South Africa; S Africa, Southern Africa. The area of the marker is proportional to the square root of the sample size. Studies with no estimate presented did not meet the inclusion criteria for this infection and age group. Year presented is the median year that baseline data were collected during the study.
(TIF)

S8 Fig High-titer, active syphilis prevalence among 25–49-year-olds, stratified by initial region and population type category. E Africa, Eastern Africa; SA, South Africa; S Africa, Southern Africa. The area of the marker is proportional to the square root of the sample size. Studies with no estimate presented did not meet the inclusion criteria for this infection and age group. Year presented is the median year that baseline data were collected during the study.
(TIF)

S9 Fig Syphilis overall prevalence among 15–24-year-olds among studies with titer information. E Africa, Eastern Africa; SA, South Africa; S/E Africa, Southern and Eastern Africa. The area of the marker is proportional to the square root of the sample size. Studies with no estimate presented did not meet the inclusion criteria for this infection and age group. Year presented is the median year that baseline data were collected during the study. *Includes studies of HIV-discordant couples.
(TIF)

S10 Fig Syphilis overall prevalence among 25–49-year-olds among studies with titer information. E Africa, Eastern Africa; SA, South Africa; S/E Africa, Southern and Eastern Africa. The area of the marker is proportional to the square root of the sample size. Studies with no estimate presented did not meet the inclusion criteria for this infection and age group. Year presented is the median year that baseline data were collected during the study. *Includes studies of HIV-discordant couples.
(TIF)

S11 Fig Syphilis overall prevalence among 15–24-year-olds among studies with titer information, stratified by initial region and population type category. E Africa, Eastern Africa; SA, South Africa; S Africa, Southern Africa. The area of the marker is proportional to the square root of the sample size. Studies with no estimate presented did not meet the inclusion criteria for this infection and age group. Year presented is the median year that baseline data were collected during the study.
(TIF)

S12 Fig Syphilis overall prevalence among 25–49-year-olds among studies with titer information, stratified by initial region and population type category. E Africa, Eastern Africa; SA, South Africa; S Africa, Southern Africa. The area of the marker is proportional to the square root of the sample size. Studies with no estimate presented did not meet the inclusion criteria for this infection and age group. Year presented is the median year that baseline data were collected during the study.
(TIF)

S13 Fig Trichomoniasis prevalence among 15–24-year-olds, stratified by initial region and population type category. E Africa, Eastern Africa; SA, South Africa; S Africa, Southern Africa. The area of the marker is proportional to the square root of the sample size. Studies with no estimate presented did not meet the inclusion criteria for this infection and age group. Year presented is the median year that baseline data were collected during the study.
(TIF)

S14 Fig Trichomoniasis prevalence among 25–49-year-olds, stratified by initial region and population type category. E Africa, Eastern Africa; SA, South Africa; S Africa, Southern Africa. The area of the marker is proportional to the square root of the sample size. Studies with no estimate presented did not meet the inclusion criteria for this infection and age group. Year presented is the median year that baseline data were collected during the study.
(TIF)

S15 Fig HSV-2 prevalence among 15–24-year-olds, stratified by initial region and population type category. E Africa, Eastern Africa; SA, South Africa; S Africa, Southern Africa. The area of the marker is proportional to the square root of the sample size. Studies with no estimate presented did not meet the inclusion criteria for this infection and age group. Year presented is the median year that baseline data were collected during the study.
(TIF)

S16 Fig HSV-2 prevalence among 25–49-year-olds, stratified by initial region and population type category. E Africa, Eastern Africa; SA, South Africa; S Africa, Southern Africa. The area of the marker is proportional to the square root of the sample size. Studies with no estimate presented did not meet the inclusion criteria for this infection and age group. Year presented is the median year that baseline data were collected during the study.
(TIF)

S17 Fig Bacterial vaginosis prevalence among 15–24-year-olds, stratified by initial region and population type category. E Africa, Eastern Africa; SA, South Africa; S Africa, Southern Africa. The area of the marker is proportional to the square root of the sample size. Studies with no estimate presented did not meet the inclusion criteria for this infection and age group. Year presented is the median year that baseline data were collected during the study.
(TIF)

S18 Fig Bacterial vaginosis prevalence among 25–49-year-olds, stratified by initial region and population type category. E Africa, Eastern Africa; SA, South Africa; S Africa, Southern Africa. The area of the marker is proportional to the square root of the sample size. Studies with no estimate presented did not meet the inclusion criteria for this infection and age group. Year presented is the median year that baseline data were collected during the study.
(TIF)

S1 Table Inclusion and exclusion criteria for HC-HIV meta-analysis dataset. (DOCX)

S2 Table Studies considered for inclusion in the STI/BV IPD meta-analysis by STI/BV and test technology used. (DOCX)

S3 Table Baseline characteristics by study population, all ages. (DOCX)

S4 Table Data availability of component datasets for the IPD meta-analysis. (DOCX)

S1 Text PRISMA checklist. (DOCX)

S2 Text IPD checklist. (DOCX)

S3 Text Protocol: Sexually transmitted infections among women in sub-Saharan Africa: Individual participant data meta-analysis of prospective studies. (DOCX)

## Abbreviations

BV: bacterial vaginosis
CI: confidence interval
EIA: enzyme immunoassay
Global Strategy: Global Health Sector Strategy on Sexually Transmitted Infections 2016–2021
HC: hormonal contraceptive
HSV: herpes simplex virus
HSV-2: herpes simplex virus type 2
IPD: individual participant data
LMICs: low- and middle-income countries
NAAT: nucleic acid amplification test
RPR: rapid plasma reagin
STI: sexually transmitted infection
WHO: World Health Organization
