# Correction: Prevalence of sexually transmitted infections and bacterial vaginosis among women in sub-Saharan Africa: An individual participant data meta-analysis of 18 HIV prevention studies

**DOI:** 10.1371/journal.pmed.1002608

**Published:** 2018-06-26

**Authors:** Elizabeth A. Torrone, Charles S. Morrison, Pai-Lien Chen, Cynthia Kwok, Suzanna C. Francis, Richard J. Hayes, Katharine J. Looker, Sheena McCormack, Nuala McGrath, Janneke H. H. M. van de Wijgert, Deborah Watson-Jones, Nicola Low, Sami L. Gottlieb, on behalf of the STIMA Working Group

The copyright license for this article was incorrectly noted as Creative Commons CC0 Public Domain when it should have been a Creative Commons Attribution License (CC BY 4.0). The text of the correct license is as follows: © 2018 Torrone et al. This is an open access article distributed under the terms of the Creative Commons Attribution License, which permits unrestricted use, distribution, and reproduction in any medium, provided the original author and source are credited.

## References

[1] Torrone EA, Morrison CS, Chen P-L, Kwok C, Francis SC, Hayes RJ, et al (2018) Prevalence of sexually transmitted infections and bacterial vaginosis among women in sub-Saharan Africa: An individual participant data meta-analysis of 18 HIV prevention studies. PLoS Med 15(2): e1002511 https://doi.org/10.1371/journal.pmed.1002511 29485986 10.1371/journal.pmed.1002511 PMC5828349

