# Supplementary figures and images for: Prevalence of sexually transmitted infections and bacterial vaginosis among women in sub-Saharan Africa: An individual participant data meta-analysis of 18 HIV prevention studies
Source: PLoS Med. 2018 Feb 27;15(2):e1002511. doi: 10.1371/journal.pmed.1002511 (PMC5828349; doi:10.1371/journal.pmed.1002511)

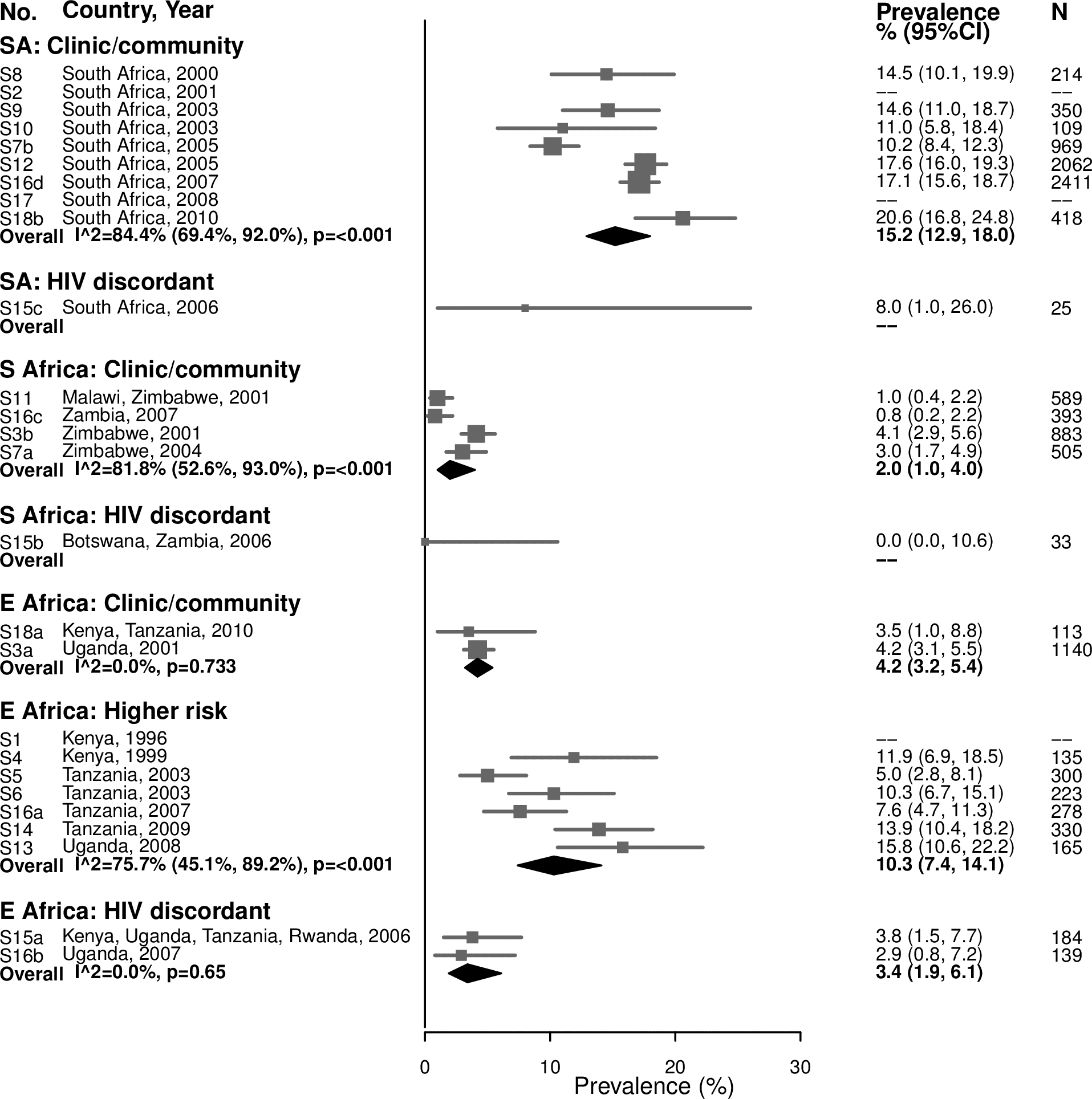

Supplement: S1 Fig — E Africa, Eastern Africa; SA, South Africa; S Africa, Southern Africa. The area of the marker is proportional to the square root of the sample size. Studies with no estimate presented did not meet the inclusion criteria for this infection and age group. Year presented is the median year that baseline data were collected during the study. (TIF) [file pmed.1002511.s001.tif]

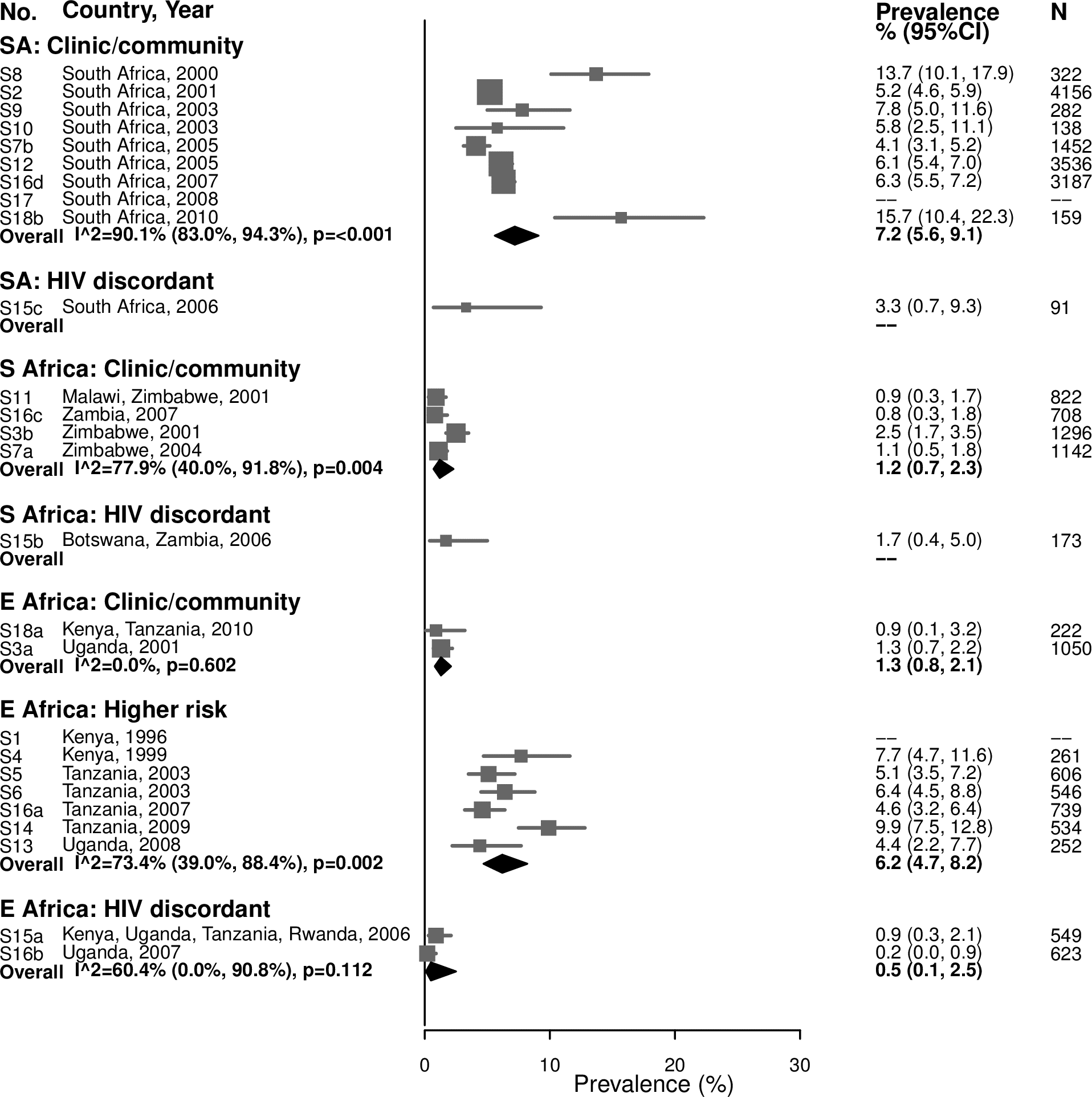

Supplement: S2 Fig — E Africa, Eastern Africa; SA, South Africa; S Africa, Southern Africa. The area of the marker is proportional to the square root of the sample size. Studies with no estimate presented did not meet the inclusion criteria for this infection and age group. Year presented is the median year that baseline data were collected during the study. (TIF) [file pmed.1002511.s002.tif]

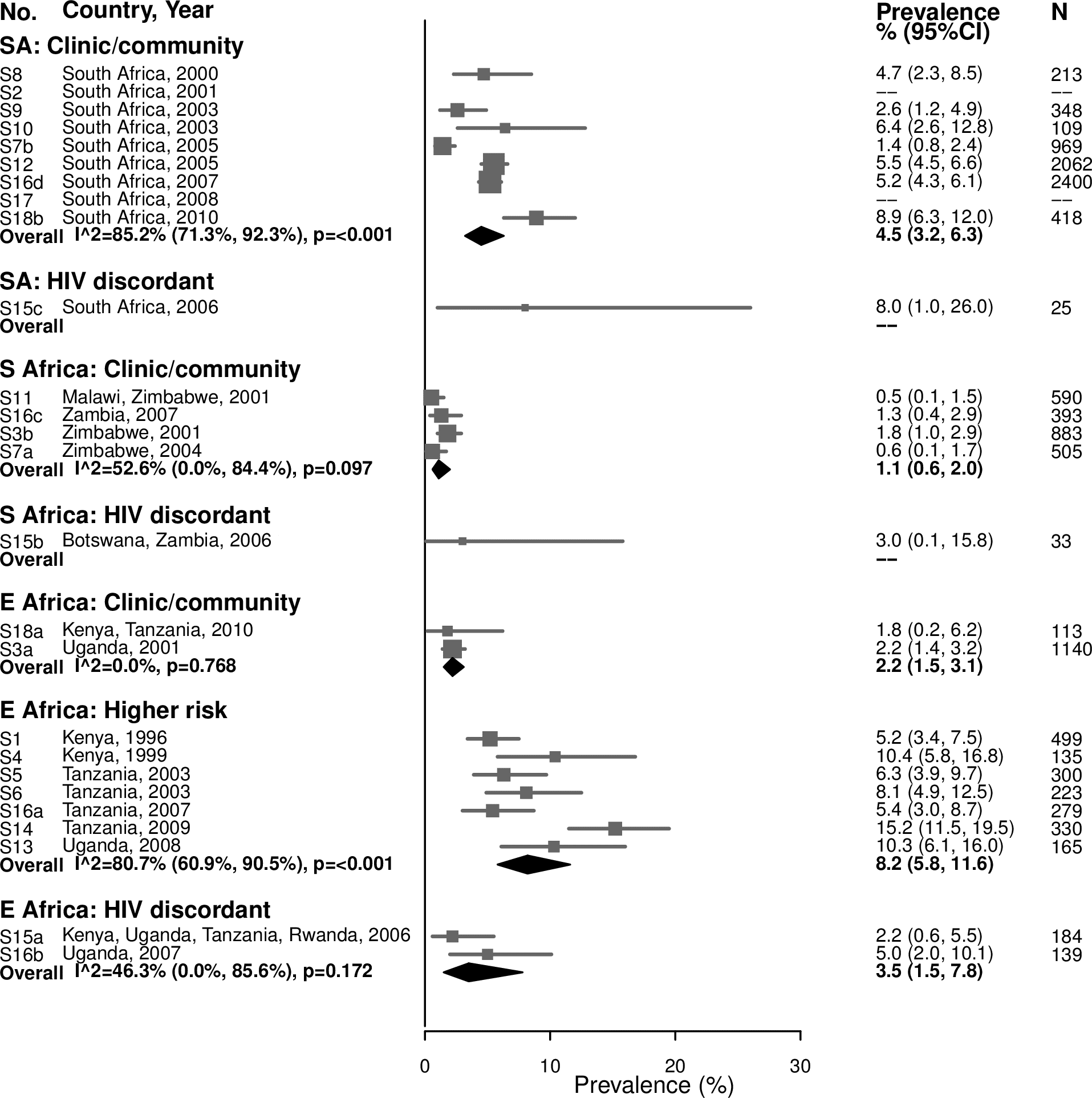

Supplement: S3 Fig — E Africa, Eastern Africa; SA, South Africa; S Africa, Southern Africa. The area of the marker is proportional to the square root of the sample size. Studies with no estimate presented did not meet the inclusion criteria for this infection and age group. Year presented is the median year that baseline data were collected during the study. (TIF) [file pmed.1002511.s003.tif]

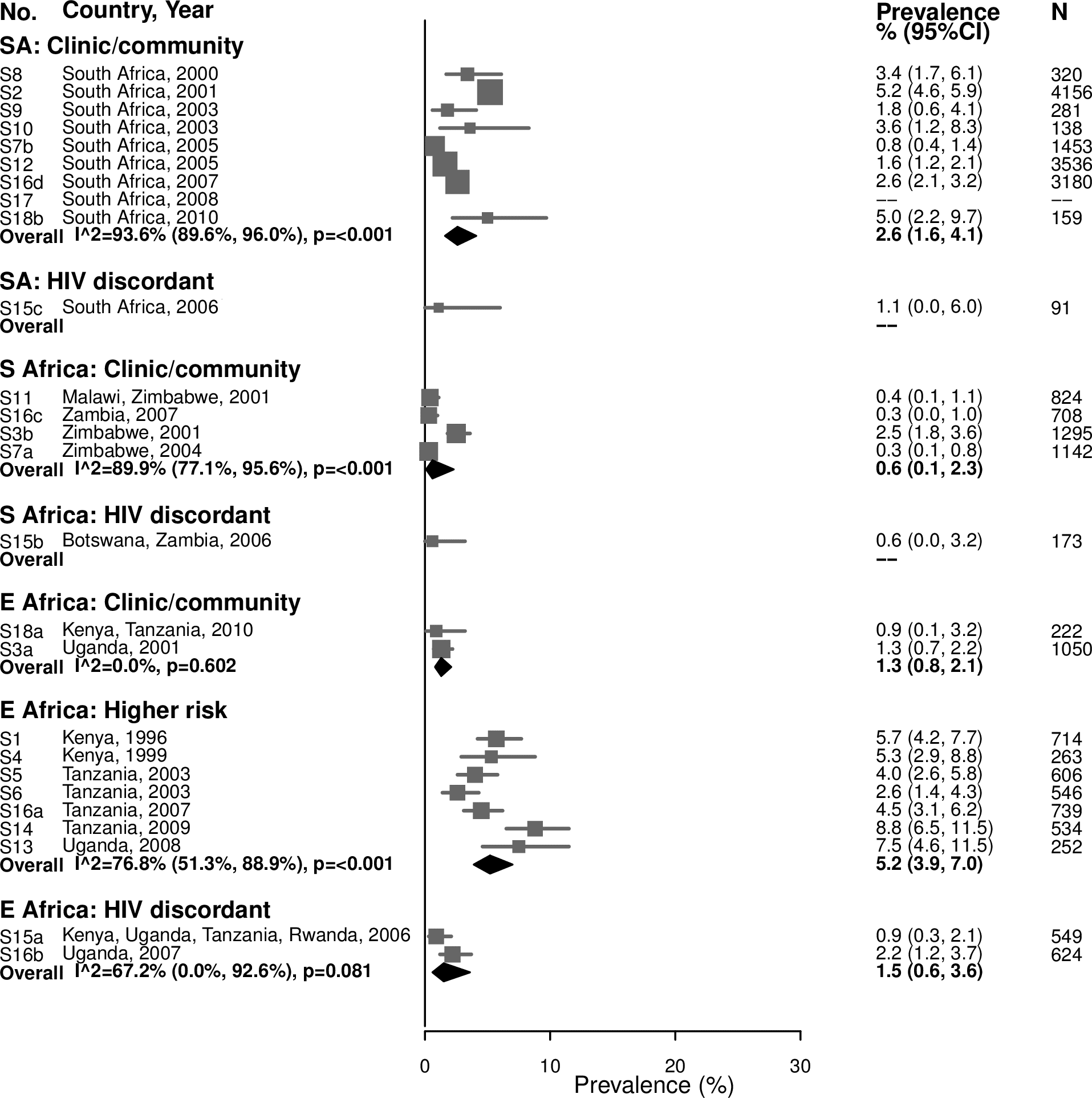

Supplement: S4 Fig — E Africa, Eastern Africa; SA, South Africa; S Africa, Southern Africa. The area of the marker is proportional to the square root of the sample size. Studies with no estimate presented did not meet the inclusion criteria for this infection and age group. Year presented is the median year that baseline data were collected during the study. (TIF) [file pmed.1002511.s004.tif]

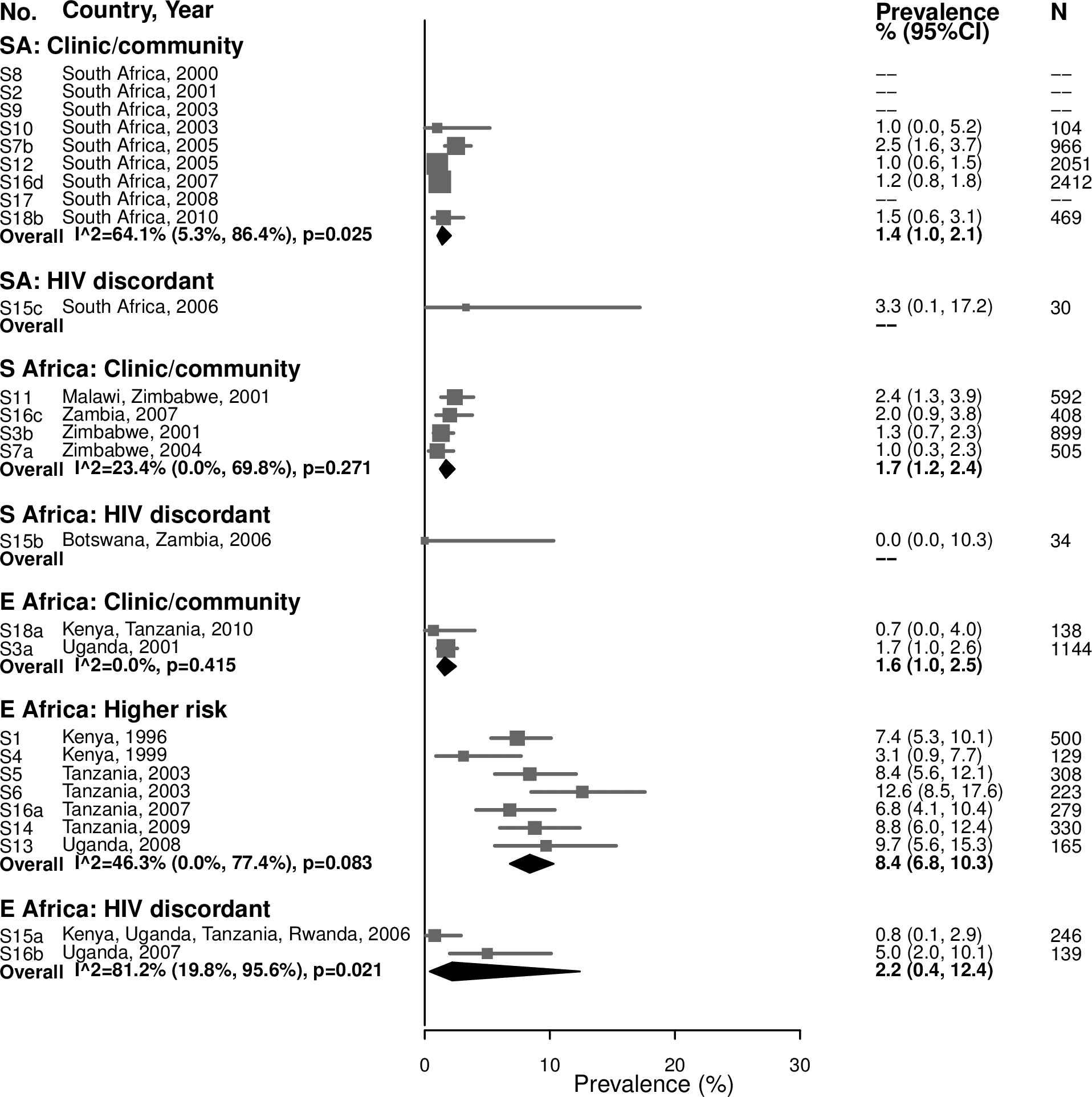

Supplement: S5 Fig — E Africa, Eastern Africa; SA, South Africa; S Africa, Southern Africa. The area of the marker is proportional to the square root of the sample size. Studies with no estimate presented did not meet the inclusion criteria for this infection and age group. Year presented is the median year that baseline data were collected during the study. (TIF) [file pmed.1002511.s005.tif]

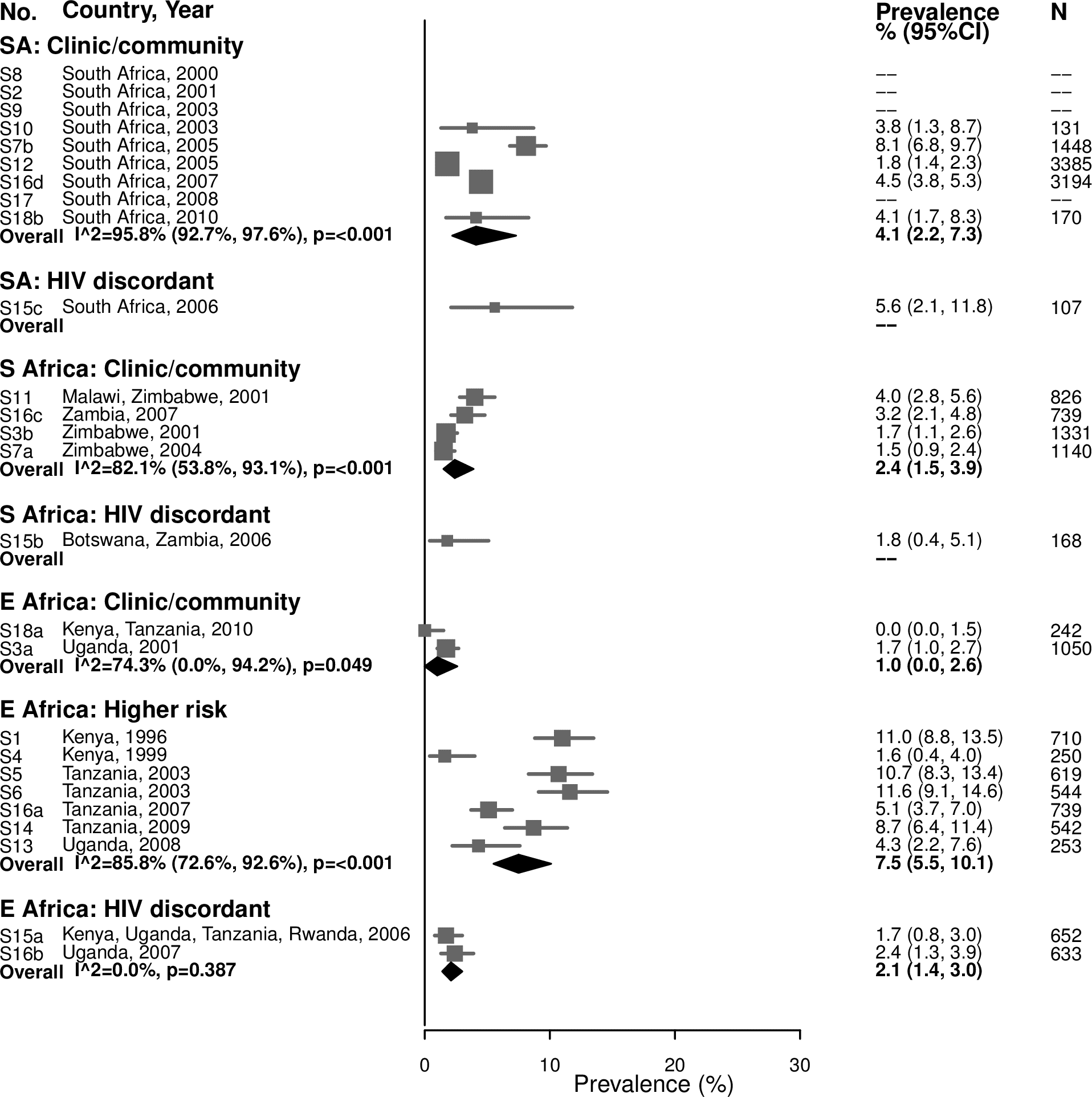

Supplement: S6 Fig — E Africa, Eastern Africa; SA, South Africa; S Africa, Southern Africa. The area of the marker is proportional to the square root of the sample size. Studies with no estimate presented did not meet the inclusion criteria for this infection and age group. Year presented is the median year that baseline data were collected during the study. (TIF) [file pmed.1002511.s006.tif]

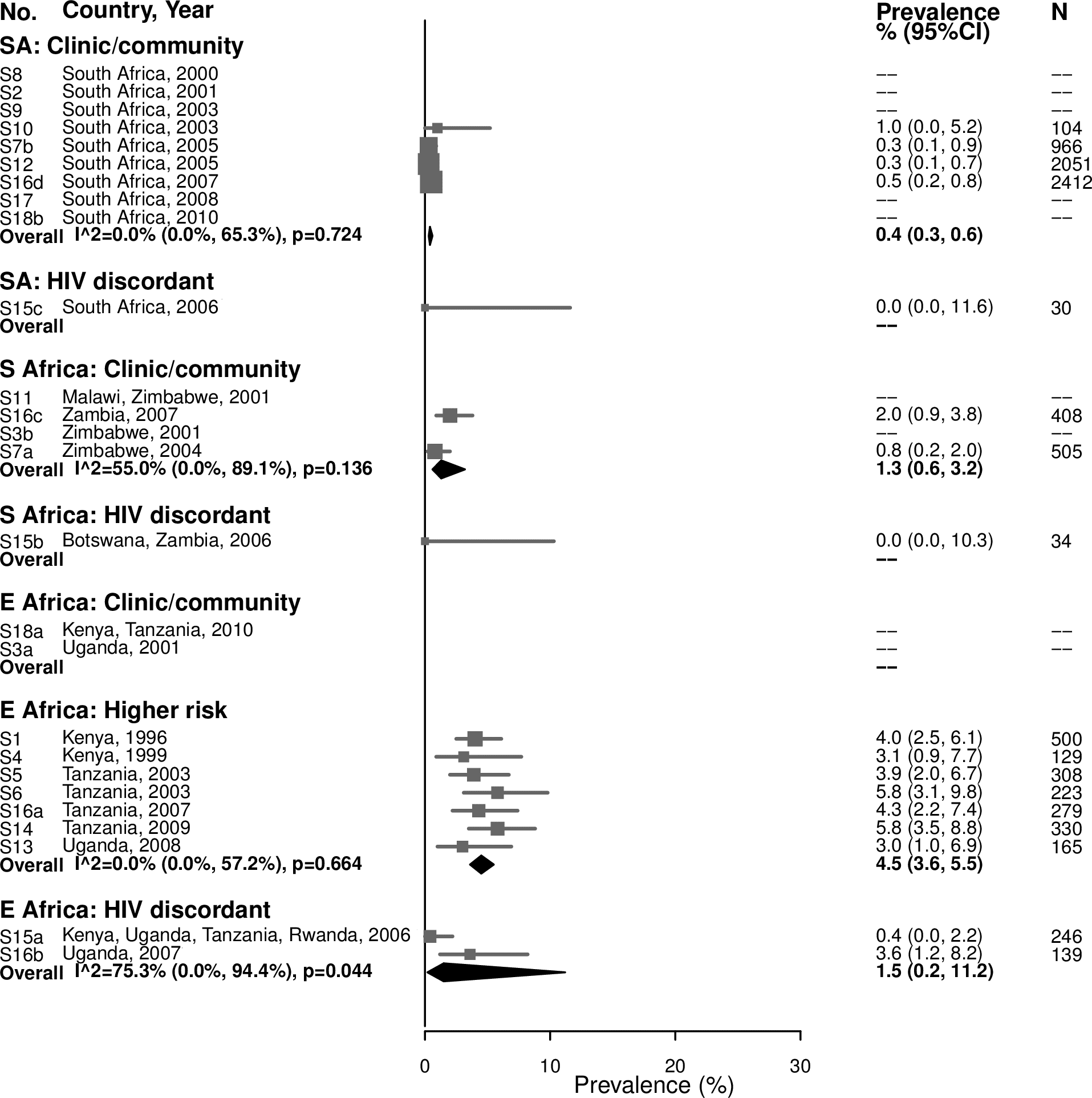

Supplement: S7 Fig — E Africa, Eastern Africa; SA, South Africa; S Africa, Southern Africa. The area of the marker is proportional to the square root of the sample size. Studies with no estimate presented did not meet the inclusion criteria for this infection and age group. Year presented is the median year that baseline data were collected during the study. (TIF) [file pmed.1002511.s007.tif]

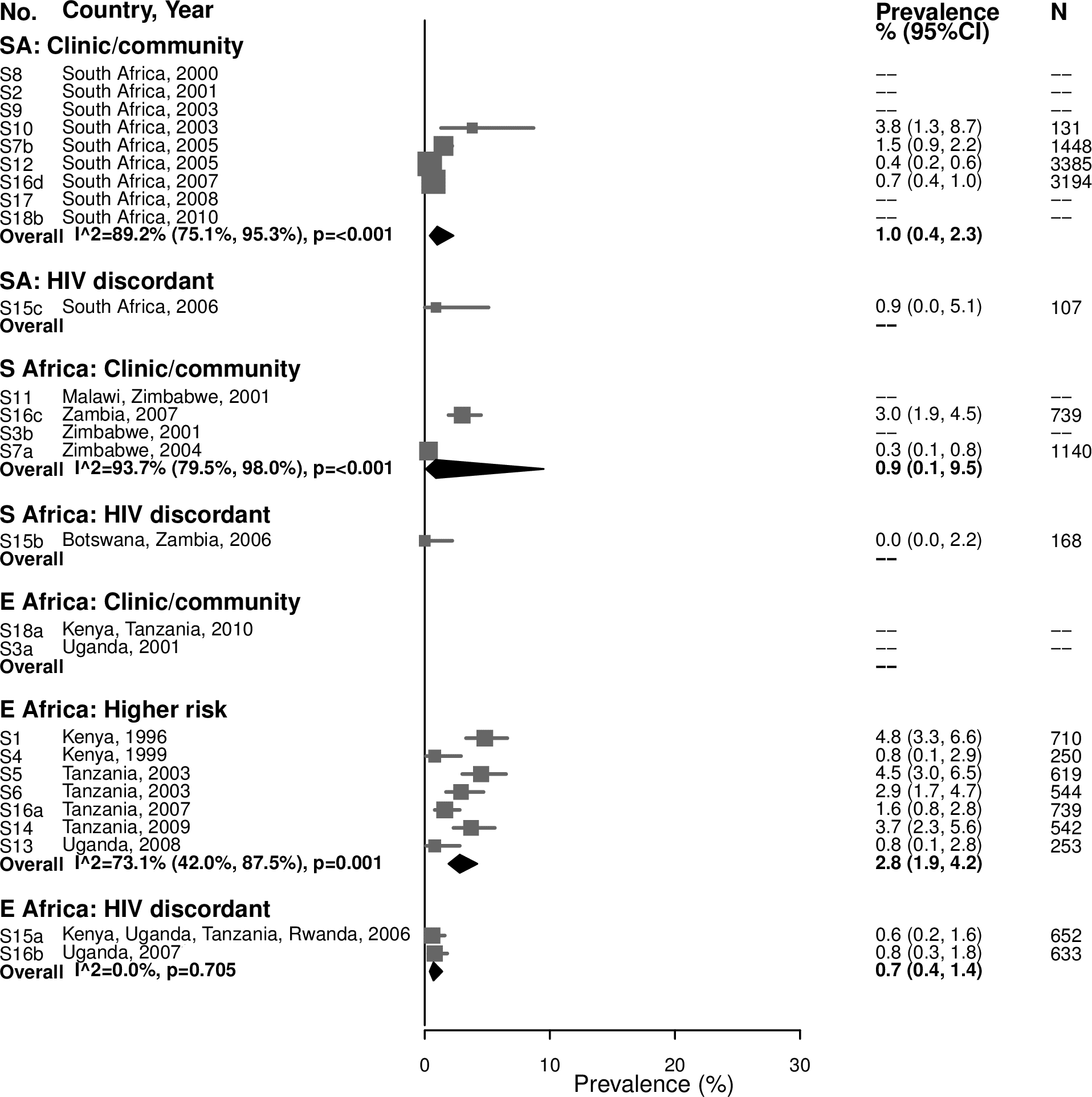

Supplement: S8 Fig — E Africa, Eastern Africa; SA, South Africa; S Africa, Southern Africa. The area of the marker is proportional to the square root of the sample size. Studies with no estimate presented did not meet the inclusion criteria for this infection and age group. Year presented is the median year that baseline data were collected during the study. (TIF) [file pmed.1002511.s008.tif]

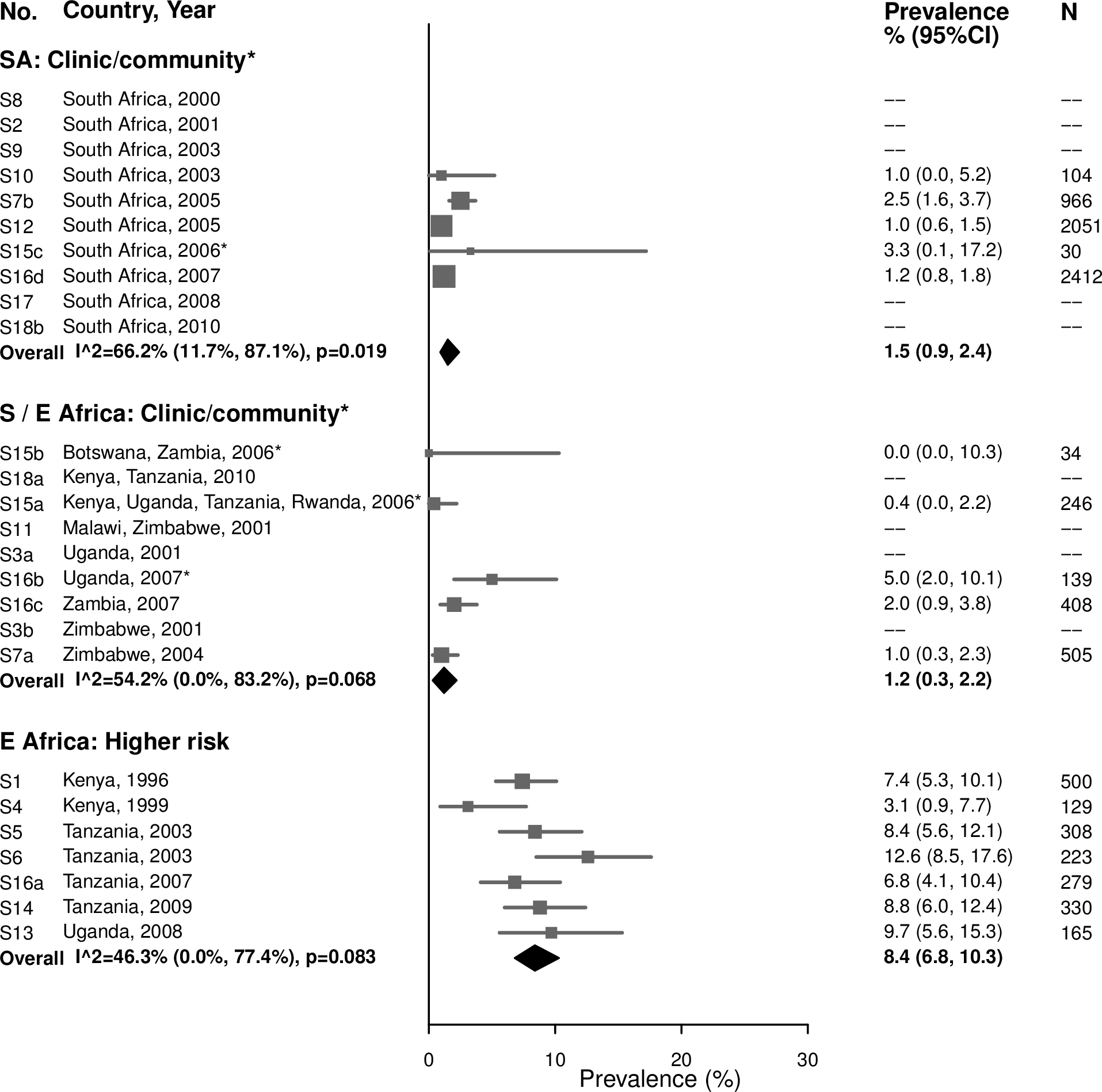

Supplement: S9 Fig — E Africa, Eastern Africa; SA, South Africa; S/E Africa, Southern and Eastern Africa. The area of the marker is proportional to the square root of the sample size. Studies with no estimate presented did not meet the inclusion criteria for this infection and age group. Year presented is the median year that baseline data were collected during the study. *Includes studies of HIV-discordant couples. (TIF) [file pmed.1002511.s009.tif]

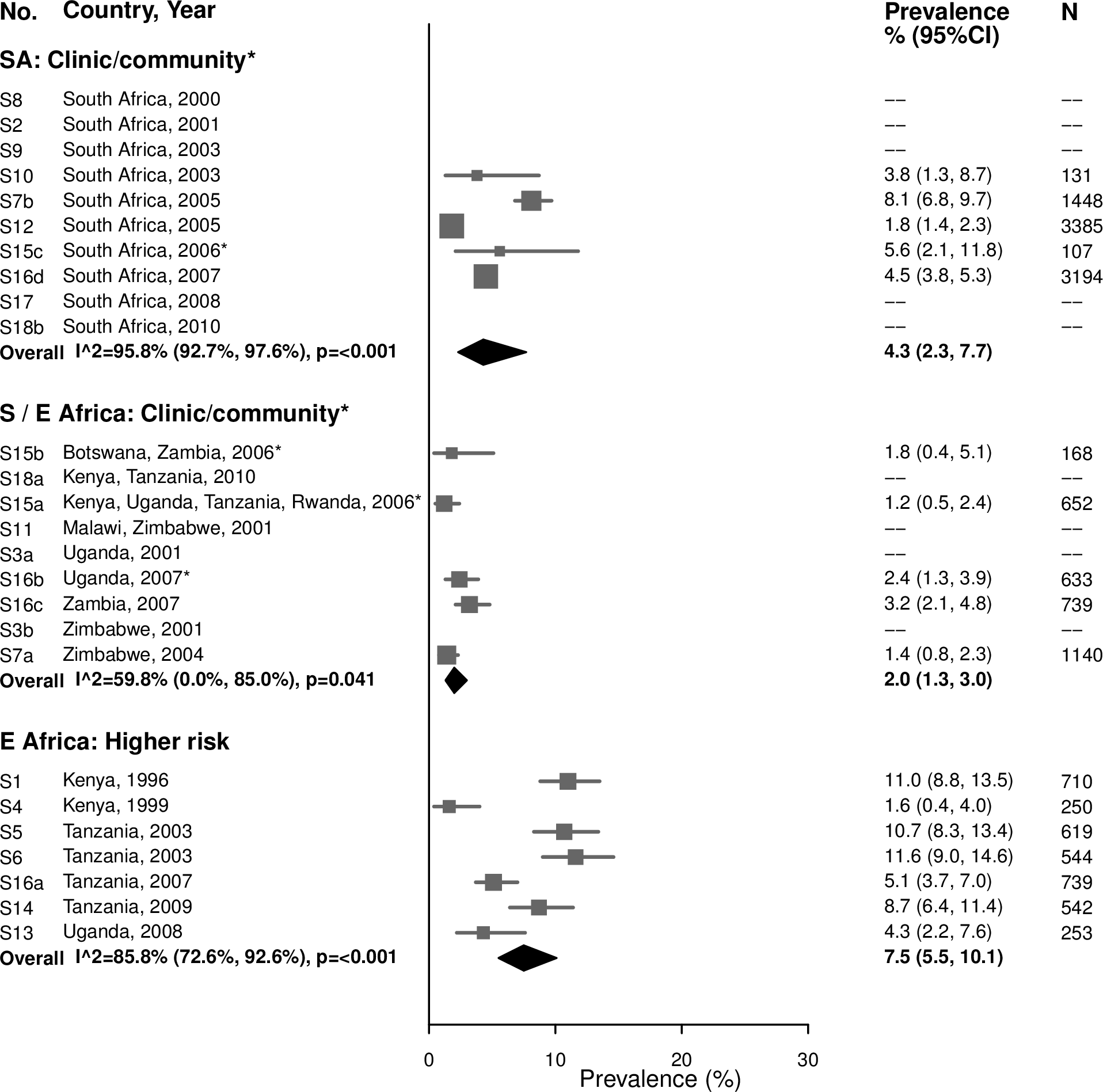

Supplement: S10 Fig — E Africa, Eastern Africa; SA, South Africa; S/E Africa, Southern and Eastern Africa. The area of the marker is proportional to the square root of the sample size. Studies with no estimate presented did not meet the inclusion criteria for this infection and age group. Year presented is the median year that baseline data were collected during the study. *Includes studies of HIV-discordant couples. (TIF) [file pmed.1002511.s010.tif]

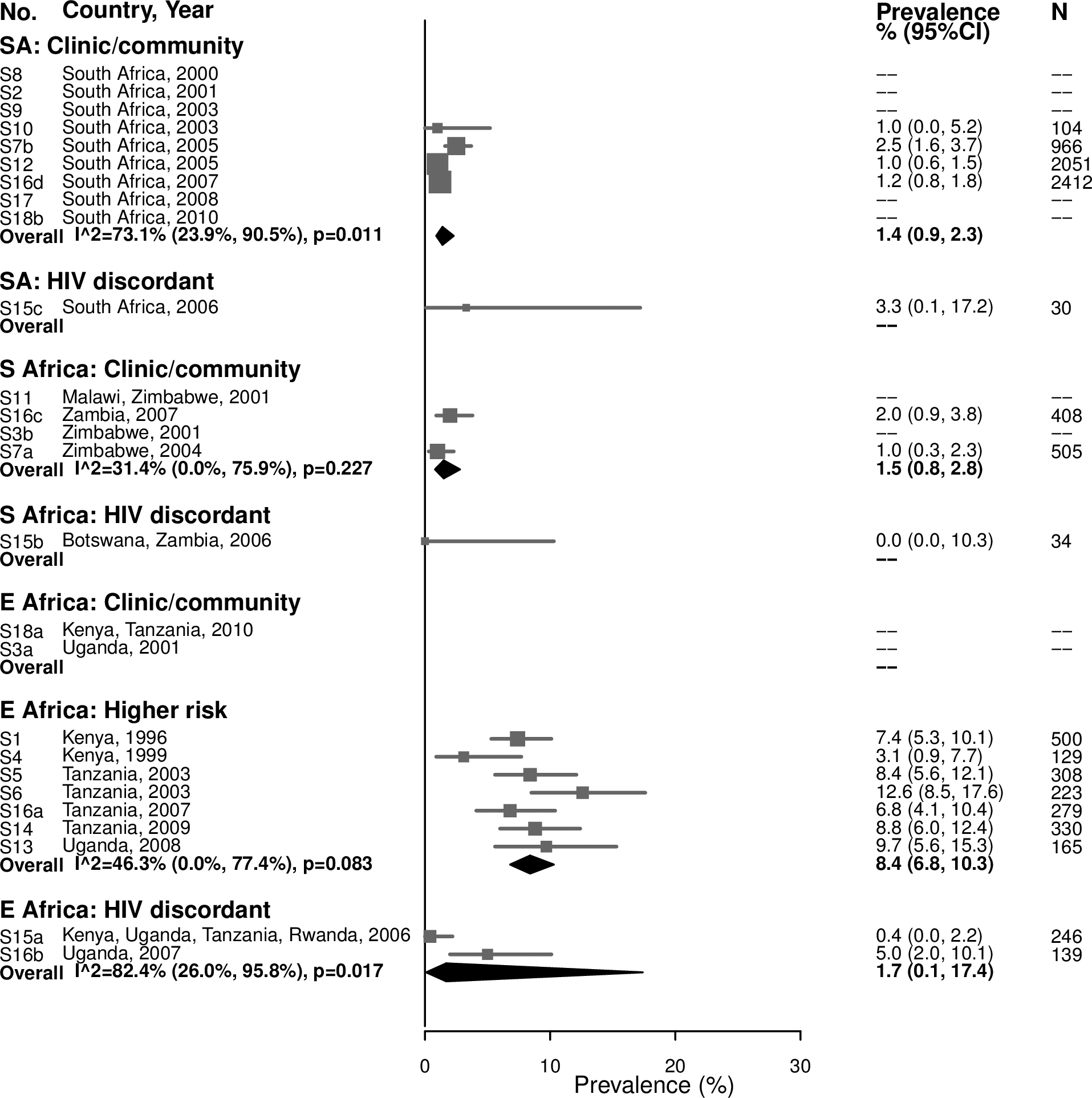

Supplement: S11 Fig — E Africa, Eastern Africa; SA, South Africa; S Africa, Southern Africa. The area of the marker is proportional to the square root of the sample size. Studies with no estimate presented did not meet the inclusion criteria for this infection and age group. Year presented is the median year that baseline data were collected during the study. (TIF) [file pmed.1002511.s011.tif]

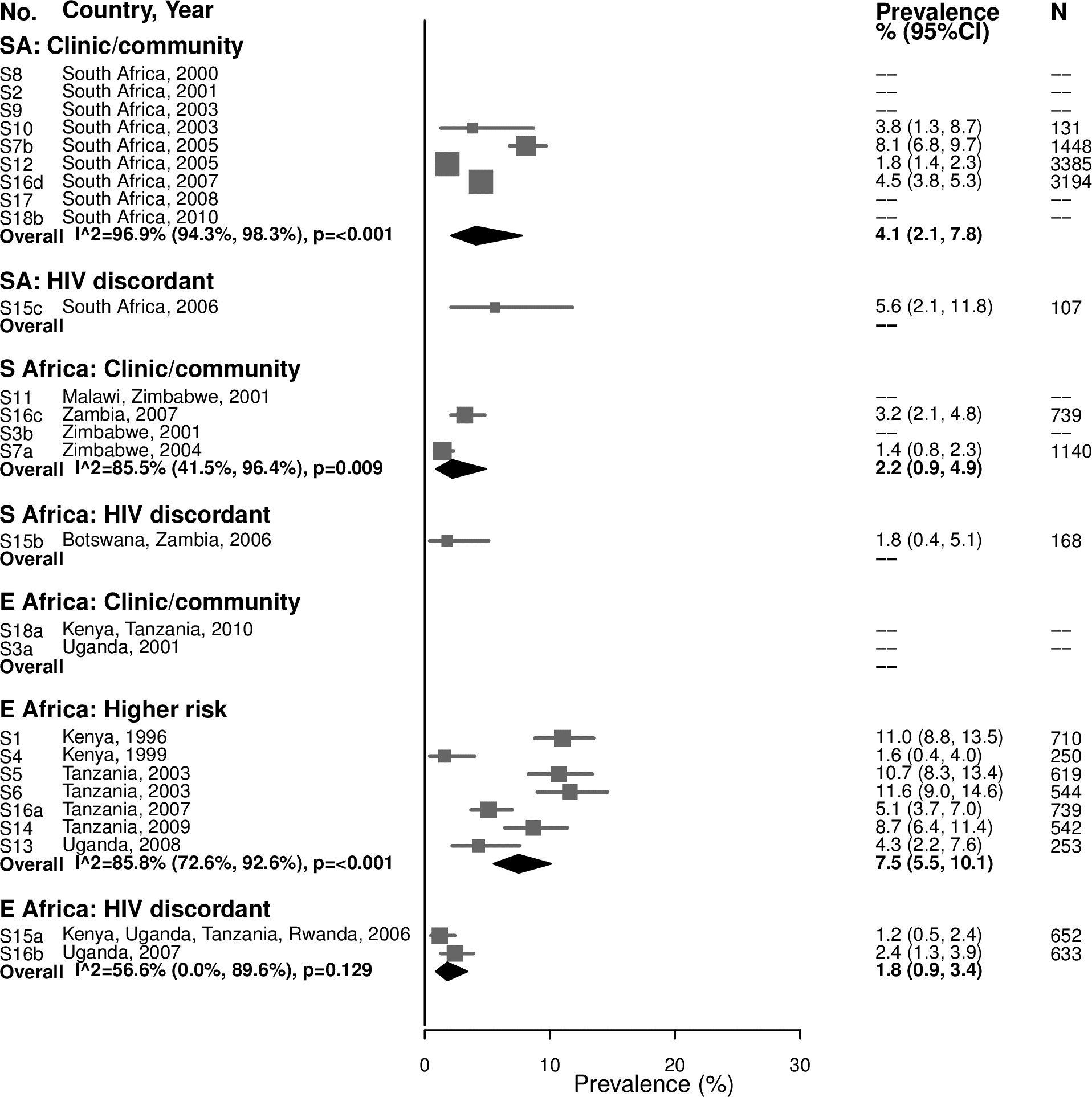

Supplement: S12 Fig — E Africa, Eastern Africa; SA, South Africa; S Africa, Southern Africa. The area of the marker is proportional to the square root of the sample size. Studies with no estimate presented did not meet the inclusion criteria for this infection and age group. Year presented is the median year that baseline data were collected during the study. (TIF) [file pmed.1002511.s012.tif]

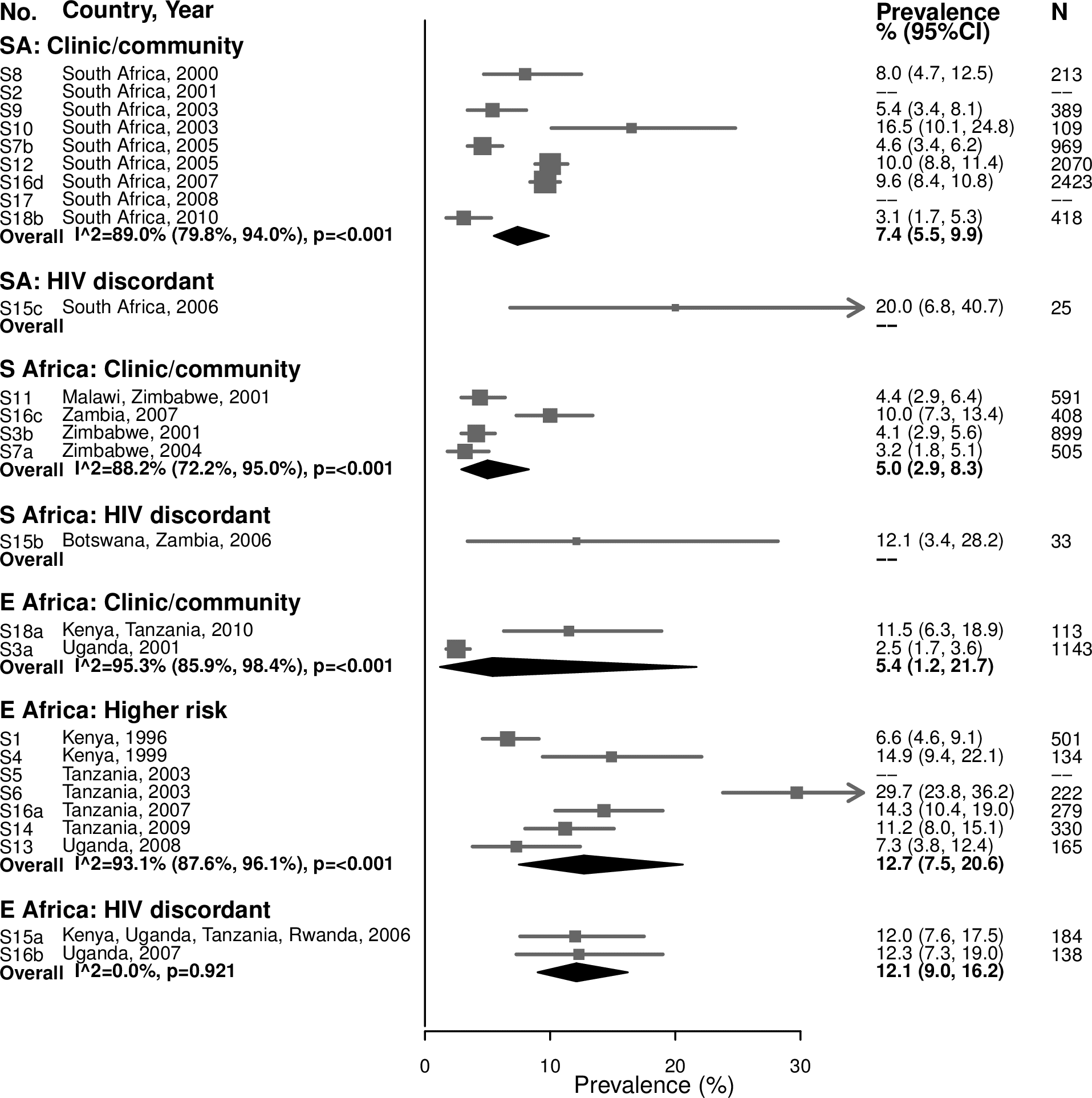

Supplement: S13 Fig — E Africa, Eastern Africa; SA, South Africa; S Africa, Southern Africa. The area of the marker is proportional to the square root of the sample size. Studies with no estimate presented did not meet the inclusion criteria for this infection and age group. Year presented is the median year that baseline data were collected during the study. (TIF) [file pmed.1002511.s013.tif]

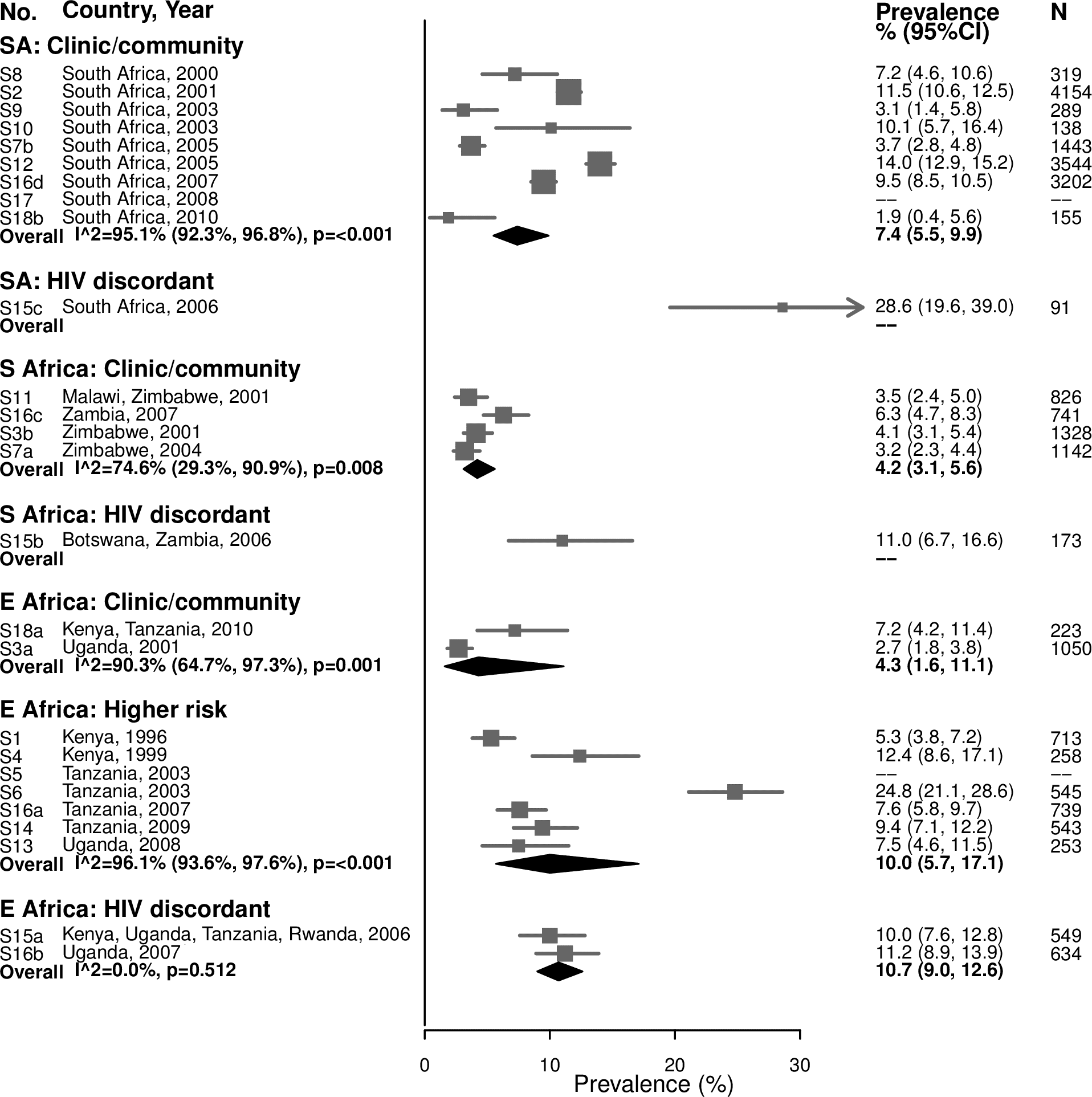

Supplement: S14 Fig — E Africa, Eastern Africa; SA, South Africa; S Africa, Southern Africa. The area of the marker is proportional to the square root of the sample size. Studies with no estimate presented did not meet the inclusion criteria for this infection and age group. Year presented is the median year that baseline data were collected during the study. (TIF) [file pmed.1002511.s014.tif]

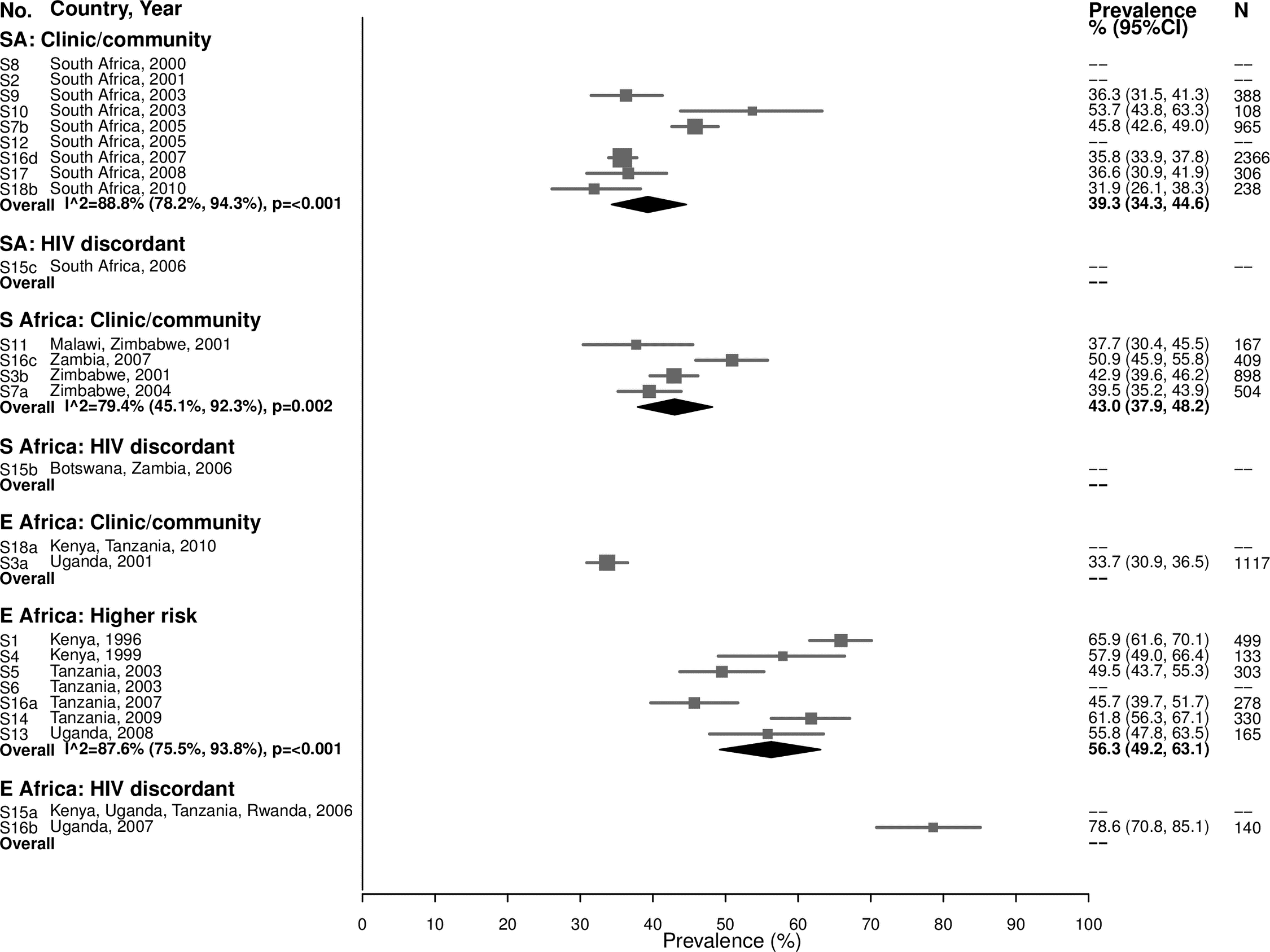

Supplement: S15 Fig — E Africa, Eastern Africa; SA, South Africa; S Africa, Southern Africa. The area of the marker is proportional to the square root of the sample size. Studies with no estimate presented did not meet the inclusion criteria for this infection and age group. Year presented is the median year that baseline data were collected during the study. (TIF) [file pmed.1002511.s015.tif]

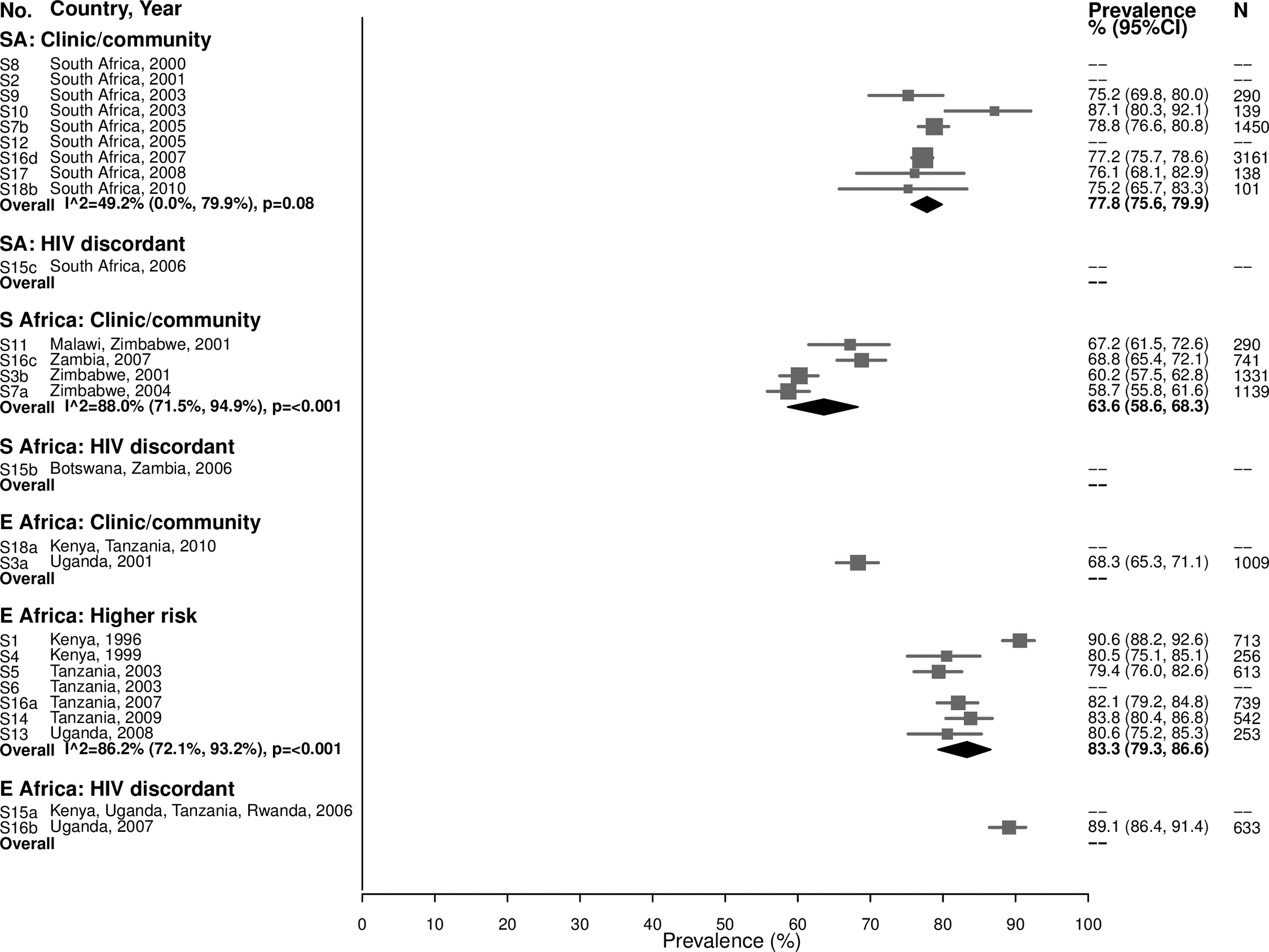

Supplement: S16 Fig — E Africa, Eastern Africa; SA, South Africa; S Africa, Southern Africa. The area of the marker is proportional to the square root of the sample size. Studies with no estimate presented did not meet the inclusion criteria for this infection and age group. Year presented is the median year that baseline data were collected during the study. (TIF) [file pmed.1002511.s016.tif]

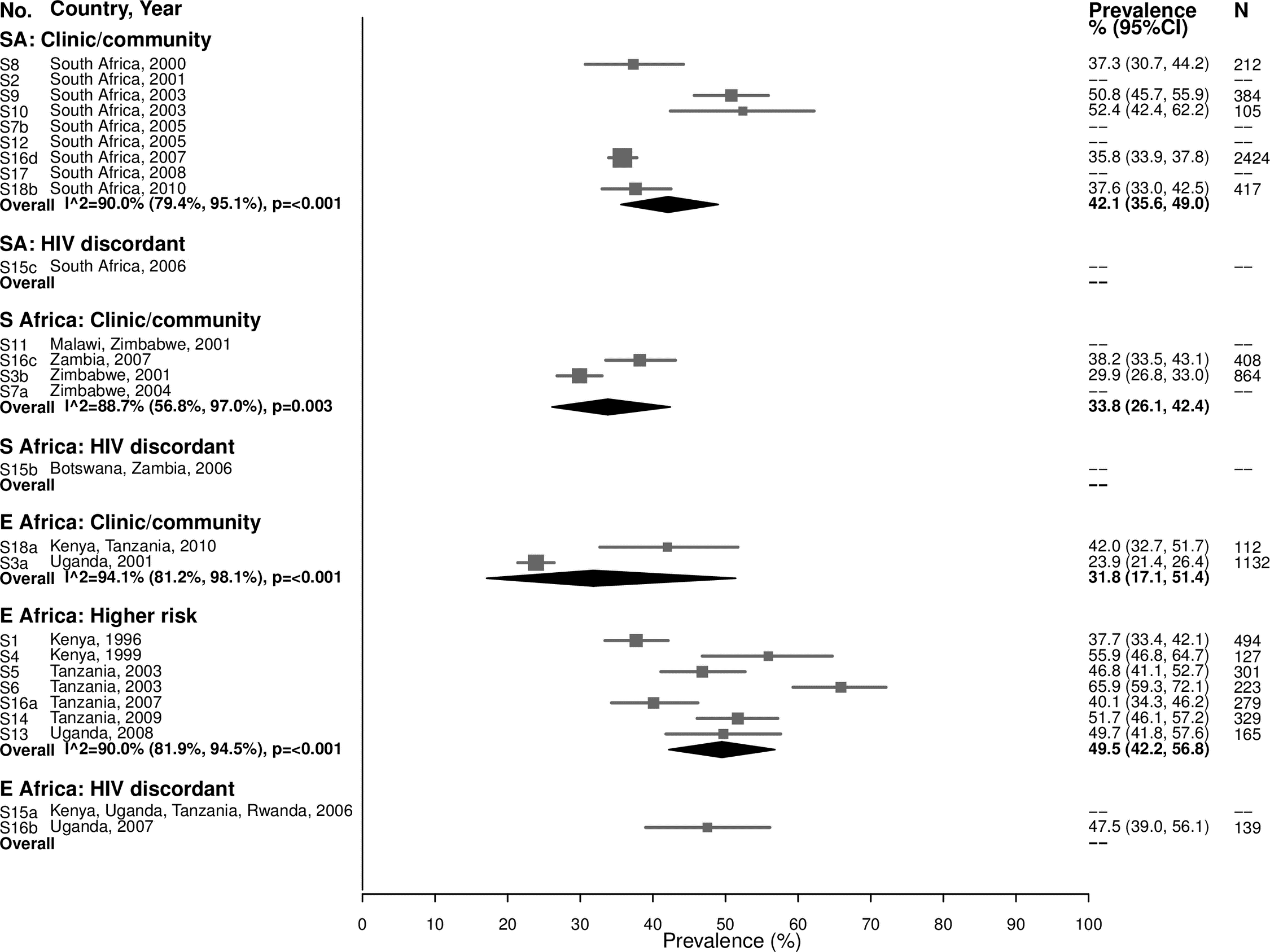

Supplement: S17 Fig — E Africa, Eastern Africa; SA, South Africa; S Africa, Southern Africa. The area of the marker is proportional to the square root of the sample size. Studies with no estimate presented did not meet the inclusion criteria for this infection and age group. Year presented is the median year that baseline data were collected during the study. (TIF) [file pmed.1002511.s017.tif]

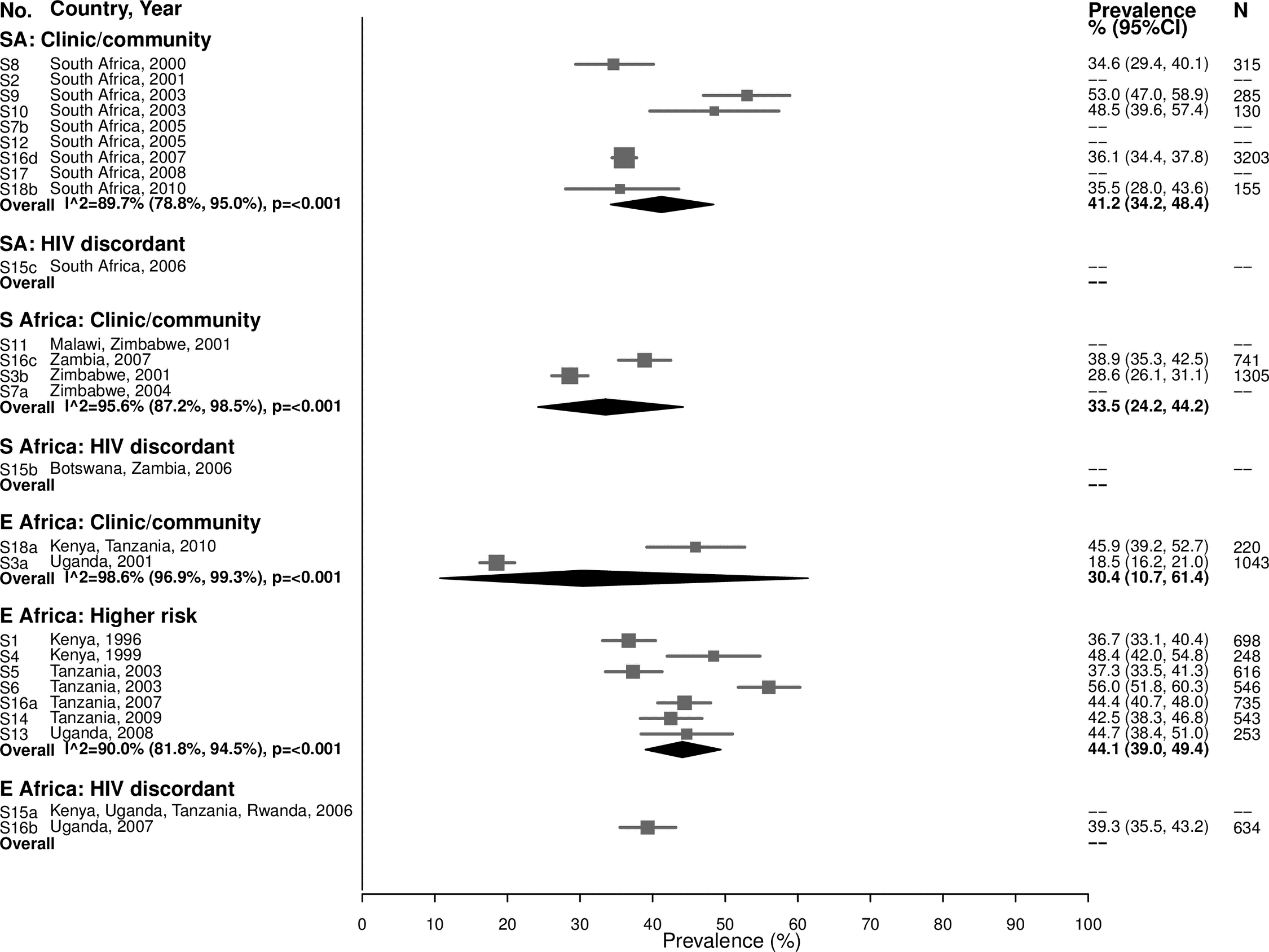

Supplement: S18 Fig — E Africa, Eastern Africa; SA, South Africa; S Africa, Southern Africa. The area of the marker is proportional to the square root of the sample size. Studies with no estimate presented did not meet the inclusion criteria for this infection and age group. Year presented is the median year that baseline data were collected during the study. (TIF) [file pmed.1002511.s018.tif]
