# Supplementary material for: Prevalence of sexually transmitted infections and bacterial vaginosis among women in sub-Saharan Africa: An individual participant data meta-analysis of 18 HIV prevention studies
Source: PLoS Med. 2018 Feb 27;15(2):e1002511. doi: 10.1371/journal.pmed.1002511 (PMC5828349; doi:10.1371/journal.pmed.1002511)
Supplement: S1 Table — (DOCX) [file pmed.1002511.s019.docx]

**Supplemental Table 1. Inclusion and exclusion criteria for HC-HIV meta-analysis dataset**

| Inclusion criteria |
| --- |
| Studies which:   - Measured HIV prospectively at multiple time points with a testing interval of 6 months or less; - Measured HIV using a standardized testing algorithm; - Measured hormonal contraceptive use prospectively at multiple time points with a measurement interval of 6 months or less; - Measured hormonal contraceptive use using a standardized questionnaire; - Included women between the ages of 15-49 years; - Included women who used injectable contraception; - Included at least 15 incident HIV infections in the dataset; - Measured important covariates including (at a minimum) age, condom use, and number of sexual partners. |
| Exclusion criteria |
| Studies which:   - Did not measure HIV infection and hormonal contraceptive use prospectively and at multiple time points with a testing interval of more than 6 months; - Did not measure hormonal contraceptive use using standardized data collection forms, and HIV using a standardized testing algorithm; - Had a significant amount (>5%) of missing HIV infection or hormonal contraceptive use data; - Had small numbers of women (or person-time) using hormonal contraception; - Had over 6 months between scheduled follow-up visits.   For studies that had an intervention arm where anti-retroviral drugs were provided (for prevention of HIV infection), women assigned to the intervention arm were excluded. |
