# Supplementary material for: Prevalence of sexually transmitted infections and bacterial vaginosis among women in sub-Saharan Africa: An individual participant data meta-analysis of 18 HIV prevention studies
Source: PLoS Med. 2018 Feb 27;15(2):e1002511. doi: 10.1371/journal.pmed.1002511 (PMC5828349; doi:10.1371/journal.pmed.1002511)
Supplement: S2 Table — (DOCX) [file pmed.1002511.s020.docx]

**S2 Table. Studies included in the STI/BV IPD meta-analysis by STI/BV and test technology used**

| Study #  [Reference] | Chlamydia  Test Type | Gonorrhea Test Type | Syphilis  Overall  Test Type | Syphilis  High titer, active infection  Test Type | Trichomoniasis  Test Type | HSV-2  Test Type | BV  Test Type |
| --- | --- | --- | --- | --- | --- | --- | --- |
| S1 [1,2] | Not included^6^ | Culture | RPR/TPHA | RPR/TPHA | Wet mount | EIA | Nugent |
| S2 [3,4] | DNA assay | DNA assay | Not included^2^ | Not included^2^ | Wet mount | Not included^2^ | Not included^2^ |
| S3a [5,6] | NAAT | NAAT | RPR/TPHA | Not included^3^ | Wet mount | EIA | Amsel & Nugent |
| S3b [5,6] | NAAT | NAAT | RPR/TPHA | Not included^3^ | Wet mount | EIA | Amsel & Nugent |
| S4 [7] | PCR | PCR & culture | RPR/TPHA | RPR/TPHA | In Pouch culture | EIA | Nugent |
| S5 [8] | PCR | PCR | RPR/TPPA | RPR/TPPA | Not included^2^ | EIA | Ison Hay |
| S6 [9,10] | PCR | PCR | RPR/TPPA | RPR/TPPA | In Pouch culture & wet mount | Not included^1^ | Nugent & Ison Hay |
| S7a [11,12] | PCR | PCR | RPR/TPHA | RPR/TPHA | PCR | EIA | Not included^2^ |
| S7b [11,12] | PCR | PCR | RPR/TPHA | RPR/TPHA | PCR | EIA | Not included^2^ |
| S8 [13] | NAAT | NAAT | Not included^3^ | Not included^3^ | Culture | Not included | Nugent |
| S9 [14] | NAAT | NAAT | Not included^3^ | Not included^3^ | NAAT | EIA | Nugent |
| S10 [15] | SDA | SDA | RPR/TPPA/TPHA | RPR/TPPA/TPHA | PCR | EIA | Nugent |
| S11 [16,17] | EIA | Culture | RPR/TPHA | Not included | Wet mount | EIA | Amsel |
| S12 [18,19] | PCR | PCR | RPR/TPHA | RPR/TPHA | In Pouch culture | Not included^?^ | Amsel |
| S13 [20] | PCR | PCR | RPR/TPHA | RPR/TPHA | In Pouch culture | EIA | Nugent |
| S14 [21] | PCR | PCR | RPR/TPPA | RPR/TPPA | In Pouch culture | EIA | Nugent |
| S15a [22,23] | NAAT | NAAT | RPR/TPHA | RPR/TPHA | NAAT | Not included^1^ | Not included^2^ |
| S15b [22,23] | NAAT | NAAT | RPR/TPHA | RPR/TPHA | NAAT | Not included | Not included^2^ |
| S15c [22,23] | NAAT | NAAT | RPR/TPHA | RPR/TPHA | NAAT | Not included | Not included^2^ |
| S16a [24,25] | NAAT | NAAT | RPR/TPHA | RPR/TPHA | In Pouch culture | EIA | Ison-hay |
| S16b [24,25] | NAAT | NAAT | RPR/TPHA | RPR/TPHA | In Pouch culture | EIA | Ison-hay |
| S16c [24,25] | NAAT | NAAT | RPR/TPHA | RPR/TPHA | In Pouch culture | EIA | Ison-hay |
| S16d [24,25] | NAAT | NAAT | RPR/TPHA | RPR/TPHA | In Pouch culture | EIA | Ison-hay |
| S17 [26] | Not included^2^ | Not included^2^ | Not included^2^ | Not included^2^ | Not included^2^ | EIA | Not included^2^ |
| S18a [27] | NAAT | NAAT | RPR/TPHA | Not included^3^ | Wet mount | Not included^?^ | Nugent |
| S18b [27] | NAAT | NAAT | RPR/TPHA | Not included^3^ | Wet mount | EIA | Nugent |
| # of studies included | **24** | **25** | **20** | **17** | **24** | **18** | **19** |
| # of studies excluded | **2** | **1** | **4** | **9** | **2** | **8** | **7** |
| Reasons for exclusion   1. Required STI for study inclusion 2. Did not test for STI/BV at baseline visit 3. Did not test for STI/BV using diagnostic test required for inclusion 4. Did not test >80% of participants (or >80% of randomly selected sample of enrolled participants) 5. >10% of participants tested had indeterminate results 6. Data not available for analysis | | | | | | | |
| BV, bacterial vaginosis; EIA, enzyme immunoassay; HSV-2, herpes simplex virus type 2; IPD, individual participant data; NAAT, nucleic acid amplification test; RPR, rapid plasma reagin; SDA, strand displacement amplification; STI, sexually transmitted infection; TPHA, *Treponema pallidum* hemagglutination assay; TPPA, *Treponema pallidum* particle agglutination assay | | | | | | | |

References

1. Baeten JM, Benki S, Chohan V, Lavreys L, McClelland RS, Mandaliya K, et al. Hormonal contraceptive use, herpes simplex virus infection, and risk of HIV-1 acquisition among Kenyan women. AIDS. 2007; 21 (13): 1771-1777.
2. Martin HL, Jr., Nyange PM, Richardson BA, Lavreys L, Mandaliya K, Jackson DJ, et al. Hormonal contraception, sexually transmitted diseases, and risk of heterosexual transmission of human immunodeficiency virus type 1. J Infect Dis. 1998;178(4):1053-9.
3. Myer L, Denny L, Wright TC, Kuhn L. Prospective study of hormonal contraception and women's risk of HIV infection in South Africa. Int J Epidemiol. 2007;36(1):166-74.
4. Myer L, Denny L, de Souza M, Wright TC, Jr., Kuhn L. Distinguishing the temporal association between women's intravaginal practices and risk of human immunodeficiency virus infection: a prospective study of South African women. Am J Epidemiol. 2006;163(6):552-60.
5. Morrison CS, Chen PL, Kwok C, Richardson BA, Chipato T, Mugerwa R, et al. Hormonal contraception and HIV acquisition: reanalysis using marginal structural modeling. AIDS. 2010;24(11):1778-81.
6. Morrison CS, Skoler-Karpoff S, Kwok C, Chen PL, van de Wijgert J, Gehret-Plagianos M, et al. Hormonal contraception and the risk of HIV acquisition. AIDS. 2007 Jan 2;21(1):85-95.
7. Kaul R, Kimani J, Nagelkerke NJ, Fonck K, Ngugi EN, Keli F, et al. Monthly antibiotic chemoprophylaxis and incidence of sexually transmitted infections and HIV-1 infection in Kenyan sex workers: a randomized controlled trial. JAMA. 2004;291(21):2555-62.
8. Vallely A, Kasindi S, Hambleton IR, Knight L, Chirwa T, Balira R, et al. Microbicides development program, Tanzania-baseline characteristics of an occupational cohort and reattendance at 3 months. Sex Transm Dis. 2007;34(9):638-43.
9. Watson-Jones D, Baisley K, Weiss HA, et al. Risk factors for HIV incidence in women participating in an HSV suppressive treatment trial in Tanzania. AIDS. 2009;23(3):415-22.
10. Watson-Jones D, Weiss HA, Rusizoka M, Changalucha J, Baisley K, Mugeye K, et al. Effect of herpes simplex suppression on incidence of HIV among women in Tanzania. N Engl J Med. 2008;358(15):1560-71.
11. McCoy SI, Zheng W, Montgomery ET, Blanchard K, van der Straten A, de Bruyn G, et al. Oral and injectable contraception use and risk of HIV acquisition among women in sub-Saharan Africa. AIDS. 2013;27(6):1001-9. doi: 10.1097/QAD.0b013e32835da401.
12. Padian NS, van der Straten A, Ramjee G, Chipato T, de Bruyn G, Blanchard K, et al. Diaphragm and lubricant gel for prevention of HIV acquisition in southern African women: a randomised controlled trial. Lancet. 2007;370(9583):251-61.
13. Kleinschmidt I, Rees H, Delany S, Smith D, Dinat N, Nkala B, et al. Injectable progestin contraceptive use and risk of HIV infection in a South African family planning cohort. Contraception. 2007;75(6):461-7.
14. Delany-Moretlwe S, Rees H. Tshireletso study for women's health. Microbicide feasibility study. Protocol. 2010. Hillbrow (South Africa): Reproductive Health Research Unit, University of Witwatersrand.
15. McGrath N, Chimbwete C, Bennish M, Cassol S, Nunn A, et al. A feasibility study in preparation for phase III microbicide trials in the Hlabisa sub-district, South Africa. 2014. Available at: https://www.southampton.ac.uk/medicine/about/staff/nmm1c12.page (Accessed 3 August 2017)
16. Kumwenda N, Kemewenda J, Kafulafula G, Makanani B, Taulo F, Nkhoma C, et al. HIV-1 incidence among women of reproductive age in Malawi. Int J STD AIDS. 2008 19: 339-341. doi: 10.1258/ijsa.2007.007165 PMID: 18482966
17. Kumwenda N, Hoffman I, Chirenje M, Kelly C, Coletti A, Ristow A, et al. HIV incidence among women of reproductive age in Malawi and Zimbabwe. Sex Transm Dis. 2006;33(11):646-51.
18. Morrison CS, Skoler-Karpoff S, Kwok C, Chen PL, van de Wijgert J, Gehret-Plagianos M, et al. Hormonal contraception and the risk of HIV acquisition among women in South Africa. AIDS. 2012;26(4):497-504
19. Skoler-Karpoff S, Ramjee G, Ahmed K, Altini L, Plagianos MG, Friedland B, et al. Efficacy of Carraguard for prevention of HIV infection in women in South Africa: a randomised, double-blind, placebo-controlled trial. Lancet. 2008;372(9654):1977-87.
20. Vandepitte J, Bukenya J, Weiss HA, Nakubulwa S, Francis SC, Hughes P, et al. HIV and other sexually transmitted infections in a cohort of women involved in high-risk sexual behavior in Kampala, Uganda. Sex Transm Dis. 2011;38(4):316-23.
21. Kapiga SH, Ewings FM, Ao T, Chilongani J, Mongi A, Baisley K, et al. The epidemiology of HIV and HSV-2 infections among women participating in microbicide and vaccine feasibility studies in Northern Tanzania. PLoS One. 2013;8(7):e68825.
22. Heffron R, Donnell D, Rees H, Celum C, Mugo N, Were E, et al. Use of hormonal contraceptives and risk of HIV-1 transmission: a prospective cohort study. Lancet Infect Dis. 2012;12(1):19-26.
23. Celum C, Wald A, Lingappa JR, Magaret AS, Wang RS, Mugo N, et al. Acyclovir and transmission of HIV-1 from persons infected with HIV-1 and HSV-2. N Engl J Med. 2010;362(5):427-39.
24. Crook AM, Ford D, Gafos M, Hayes R, Kamali A, Kapiga S, et al. Injectable and oral contraceptives and risk of HIV acquisition in women: an analysis of data from the MDP301 trial. Hum Reprod. 2014;29(8):1810-7.
25. McCormack S, Ramjee G, Kamali A, Rees H, Crook AM, Gafos M, et al. PRO2000 vaginal gel for prevention of HIV-1 infection (Microbicides Development Programme 301): a phase 3, randomised, double-blind, parallel-group trial. Lancet. 2010;376(9749):1329-37.
26. Abdool Karim Q, Abdool Karim SS, Frohlich JA, Grobler AC, Baxter C, Mansoor LE, et al. Effectiveness and safety of tenofovir gel, an antiretroviral microbicide, for the prevention of HIV infection in women. Science. 2010;329(5996):1168-74.
27. Van Damme L, Corneli A, Ahmed K, Agot K, Lombaard J, Kapiga S, et al. Preexposure prophylaxis for HIV infection among African women. N Engl J Med. 2012;367(5):411-22.
