# Supplementary material for: Prevalence of sexually transmitted infections and bacterial vaginosis among women in sub-Saharan Africa: An individual participant data meta-analysis of 18 HIV prevention studies
Source: PLoS Med. 2018 Feb 27;15(2):e1002511. doi: 10.1371/journal.pmed.1002511 (PMC5828349; doi:10.1371/journal.pmed.1002511)
Supplement: S3 Table — (DOCX) [file pmed.1002511.s021.docx]

S3 Table. Baseline characteristics by region/population type

| **Characteristic** | **Eastern African** | | | **Southern African** | | **South African** | |
| --- | --- | --- | --- | --- | --- | --- | --- |
|  | **Clinic or community-based (*N*= 2574)** | **Higher-risk**  **(*N*= 5626)** | **HIV-discordant couples**  **(*N*= 1687)** | **Clinic or community-based (*N*= 6452)** | **HIV-discordant couples**  **(*N*= 214)** | **Higher-risk**  **(*N*= 5626)** | **HIV-discordant couples**  **(*N*= 1687)** |
| ***Sociodemographics*** |  |  |  |  |  |  |  |
| **Age group** |  |  |  |  |  |  |  |
| 15 – 24 | 1282 (49.8) | 1942 (34.5) | 393 (23.3) | 2409 (37.3) | 37 (17.3) | 6978 (34.2) | 31 (22.5) |
| 25 - 49 | 1292 (50.2) | 3684 (65.5) | 1294 (76.7) | 4043 (62.7) | 177 (82.7) | 13449 (65.8) | 107 (77.5) |
| **Total** | 2574 (100) | 5626 (100) | 1687 (100) | 6452 (100) | 214 (100) | 20427 (100) | 138 (100) |
| Missing | 0 | 0 | 0 | 0 | 0 | 0 | 0 |
| **Education** |  |  |  |  |  |  |  |
| No school | 63 (2.5) | 471 (9.0) | 167 (9.9) | 269 (4.2) | 12 (5.6) | 778 (4.8) | 3 (2.2) |
| Primary incomplete | 590 (22.9) | 1217 (23.3) | 671 (39.8) | 566 (8.8) | 53 (24.8) | 2068 (12.7) | 16 (11.6) |
| Primary complete | 444 (17.3) | 2588 (49.5) | 270 (16.0) | 1494 (23.2) | 45 (21.0) | 1176 (7.3) | 5 (3.6) |
| Secondary incomplete | 994 (38.6) | 651 (12.5) | 387 (22.9) | 3759 (58.3) | 61 (28.5) | 6836 (42.1) | 46 (33.3) |
| Secondary complete | 233 (9.1) | 186 (3.6) | 150 (8.9) | 319 (4.9) | 27 (12.6) | 4519 (27.8) | 61 (44.2) |
| Tertiary level | 250 (9.7) | 112 (2.4) | 42 (2.5) | 45 (0.7) | 16 (7.5) | 854 (5.3) | 7 (5.1) |
| **Total** | 2574 (100) | 5225 (92.8) | 1687 (100) | 6452 (100) | 214 (100) | 16231 (79.5) | 138 (100) |
| Missing | 0 | 401 | 0 | 0 | 0 | 4196 | 0 |
| **Married/Living with partner** |  |  |  |  |  |  |  |
| Yes | 1980 (76.9) | 1156 (20.6) | 1615 (95.7) | 6161 (95.5) | 195 (91.1) | 7004 (34.3) | 91 (65.9) |
| No | 594 (23.1) | 4469 (79.5) | 72 (4.3) | 291 (4.5) | 19 (8.9) | 13418 (65.7) | 47 (34.1) |
| **Total** | 2574 (100) | 5625 (99.9) | 1687 (100) | 6452 (100) | 214 (100) | 20422 (99.9) | 138 (100) |
| Missing | 0 | 1 | 0 | 0 | 0 | 5 | 0 |

| **Characteristic** | **Eastern African** | | | **Southern African** | | **South African** | |
| --- | --- | --- | --- | --- | --- | --- | --- |
|  | **Clinic or community-based (*N*= 2574)** | **Higher-risk**  **(*N*= 5626)** | **HIV-discordant couples**  **(*N*= 1687)** | **Clinic or community-based (*N*= 6452)** | **HIV-discordant couples**  **(*N*= 214)** | **Higher-risk**  **(*N*= 5626)** | **HIV-discordant couples**  **(*N*= 1687)** |
| **Employed** |  |  |  |  |  |  |  |
| Yes | 1291 (50.2) | 5213 (99.8) | 314 (18.6) | 2117 (32.8) | 76 (35.5) | 3527 (21.7) | 37 (26.8) |
| No | 1283 (49.8) | 13 (0.3) | 1373 (81.4) | 4335 (67.2) | 138 (64.5) | 12745 (78.3) | 101 (73.2) |
| **Total** | 2574 (100) | 5226 (92.9) | 1687 (100) | 6452 (100) | 214 (100) | 16272 (79.7) | 138 (100) |
| Missing | 0 | 400 | 0 | 0 | 0 | 4155 | 0 |
| **Number of live births** | 2 (1-4) 2280 | 2 (1-3) 4067 | (N/A) | 2 (1-3) 3861 | (N/A) | 2 (1-3) 13906 | (N/A) 0 |
| ***Sexual Behavior*** |  |  |  |  |  |  |  |
| **Condom use at last sex** |  |  |  |  |  |  |  |
| Yes | 643 (29.3) | 1467 (38.9) | 518 (66.9) | 3105 (52.3) | N/A | 8989 (60.1) | N/A |
| No | 1551 (70.7) | 2302 (61.1) | 256 (33.1) | 2829 (47.7) | N/A | 5965 (39.9) | N/A |
| **Total** | 2194 (85.2) | 3769 (67.0) | 774 (45.9) | 5934 (92.0) | N/A | 14954 (73.2) | N/A |
| Missing | 380 | 1857 | 913 | 518 | 214 | 5473 | 138 |
| **Age at first sex** | 16 (15-18) | 17 (15-18) | 17 (17-17) | 18 (17-20) | (N/A) 0 | 17 (16-18) | (N/A) |
| **Coital frequency in past month** | 8 (4-14) | 4 (2-8) | 4 (2-8) | 12 (8-20) | 4 (2-7) 46 | 7 (4-12) | 5 (3-11) |
| **Number of lifetime partners** | 3 (2-4) | 4 (3-8) | (N/A) 0 | 1 (1-1) | (N/A) 0 | 3 (2-5) | (N/A) |
| **New partner*** |  |  |  |  |  |  |  |
| Yes | 109 (4.2) | 218 (84.2) | 1 (0.2) | 25 (0.7) | 1 (2.17) | 191 (2.8) | 0 (0.00) |
| No | 2464 (95.8) | 41 (15.8) | 632 (99.8) | 3594 (99.3) | 45 (97.8) | 6713 (97.2) | 70 (100.0) |
| **Total** | 2573 (99.9) | 259 (4.6) | 633 (37.5) | 3619 (56.1) | 46 (21.5) | 6904 (33.8) | 70 (50.7) |
| Missing | 1 | 5367 | 1054 | 2833 | 168 | 13523 | 68 |

| **Characteristic** | **Eastern African** | | | **Southern African** | | **South African** | |
| --- | --- | --- | --- | --- | --- | --- | --- |
|  | **Clinic or community-based (*N*= 2574)** | **Higher-risk**  **(*N*= 5626)** | **HIV-discordant couples**  **(*N*= 1687)** | **Clinic or community-based (*N*= 6452)** | **HIV-discordant couples**  **(*N*= 214)** | **Higher-risk**  **(*N*= 5626)** | **HIV-discordant couples**  **(*N*= 1687)** |
| **Concurrent partner**** |  |  |  |  |  |  |  |
| Yes | 323 (12.6) | 445 (19.5) | 3 (0.21) | 14 (0.4) | 2 (3.8) | 627 (3.5) | 2 (2.7) |
| No | 2251 (87.5) | 1833 (80.5) | 1428 (99.79) | 3367 (99.6) | 51 (96.2) | 17326 (96.5) | 73 (97.3) |
| **Total** | 2574 (100) | 2278 (40.5) | 1431 (84.83) | 3381 (52.4) | 53 (24.8) | 17953 (87.9) | 75 (54.4) |
| Missing | 0 | 3348 | 256 | 3071 | 161 | 2474 | 63 |
| **Baseline contraception** |  |  |  |  |  |  |  |
| Non-hormonal | 773 (30.0) | 3752 (66.9) | 1250 (74.58) | 2282 (35.5) | 165 (77.1) | 9886 (48.6) | 102 (73.9) |
| COCP | 791 (30.7) | 673 (12.0) | 72 (4.30) | 2554 (39.7) | 24 (11.2) | 1713 (8.4) | 8 (5.8) |
| DMPA | 1010 (39.2) | 1181 (21.1) | 354 (21.12) | 1527 (23.8) | 25 (11.7) | 5597 (27.5) | 28 (20.3) |
| Neten | 0 (0.00) | 0 (0.00) | 0 (0.00) | 67 (1.04) | 0 (0.00) | 3133 (15.4) | 0 (0.00) |
| **Total** | 2574 (100) | 5606 (99.6) | 1676 (99.35) | 6430 (99.7) | 214 (100) | 20329 (99.5) | 138 (100) |
| Multiple Use | 0 | 13 | 11 | 22 | 0 | 48 | 0 |
| Missing | 0 | 7 | 0 | 0 | 0 | 50 | 0 |
| Data given as *N* (percent) or median (quartile 1–quartile 3). *N*s in this table are based on all women in included studies; prevalence estimates for each infection are based on the subset of women who were tested for that infection.  * Time period to define new partnership varied by study and was either last month or last 3 months  ** Time period to define concurrency varied by study and was either current, last month, or last 3 months  Abbreviations: COCP : combined oral contraceptive pill; DMPA, depot medroxyprogesterone acetate; Neten, norethisterone enanthate | | | | | | | |
