# Supplementary material for: Prevalence of sexually transmitted infections and bacterial vaginosis among women in sub-Saharan Africa: An individual participant data meta-analysis of 18 HIV prevention studies
Source: PLoS Med. 2018 Feb 27;15(2):e1002511. doi: 10.1371/journal.pmed.1002511 (PMC5828349; doi:10.1371/journal.pmed.1002511)
Supplement: S4 Table — (DOCX) [file pmed.1002511.s022.docx]

| **S4 Table. Data availability of component datasets for the individual participant data meta-analysis** | | | |
| --- | --- | --- | --- |
| Name of Study | Study Website | Contact Person | Email |
| 1. Mombasa Sex Worker Study | N/A | Scott McClelland | [mcclell@uw.edu](file:///C:\Users\cmorrison\AppData\Local\Microsoft\Windows\Temporary%20Internet%20Files\Content.Outlook\NPHYNQGN\mcclell@uw.edu) |
| 2. Cape Town Cervical Cancer Project | N/A | Landon Myer | [landon.myer@uct.ac.za](mailto:landon.myer@uct.ac.za) |
| 3. HC-HIV Study | N/A | Jen Deese | JDeese@fhi360.org |
| 4. Kibera HIV Study | N/A | Rupert Kaul | [rupert.kaul@utoronto.ca](mailto:rupert.kaul@utoronto.ca) |
| 5. MDP Mwanza Microbicide Feasibility Study* | N/A | Saidi Kapiga | [Saidi.Kapiga@lshtm.ac.uk](mailto:Saidi.Kapiga@lshtm.ac.uk) |
| 6. HSV Intervention Study* | N/A | Saidi Kapiga | [Saidi.Kapiga@lshtm.ac.uk](mailto:Saidi.Kapiga@lshtm.ac.uk) |
| 7. MIRA Study | N/A | Ariane van der Straten  Sue Mavedzenge | [ariane@rti.org](mailto:ariane@rti.org)  smavedzenge@rti.org |
| 8. Palesa Study | N/A | Helen Rees  Sinead Delany-Moretlwe | [hrees@wrhi.ac.za](file:///C:\Users\cmorrison\AppData\Local\Microsoft\Windows\Temporary%20Internet%20Files\Content.Outlook\NPHYNQGN\hrees@wrhi.ac.za)  [sdelany@wrhi.ac.za](file:///C:\Users\cmorrison\AppData\Local\Microsoft\Windows\Temporary%20Internet%20Files\Content.Outlook\NPHYNQGN\sdelany@wrhi.ac.za) |
| 9. Tshireletso Study | N/A | Helen Rees  Sinead Delany-Moretlwe | [hrees@wrhi.ac.za](file:///C:\Users\cmorrison\AppData\Local\Microsoft\Windows\Temporary%20Internet%20Files\Content.Outlook\NPHYNQGN\hrees@wrhi.ac.za)  [sdelany@wrhi.ac.za](file:///C:\Users\cmorrison\AppData\Local\Microsoft\Windows\Temporary%20Internet%20Files\Content.Outlook\NPHYNQGN\sdelany@wrhi.ac.za) |
| 10. MDP KZN Microbicide Feasibility Study | N/A | Angela Crook | angela.crook@ucl.ac.uk |
| 11. HIV NET 016 | N/A | Joelle Brown | [Joelle.Brown@ucsf.edu](mailto:Joelle.Brown@ucsf.edu) |
| 12. Carraguard Microbicide Trial | N/A | Barbara Friedland  Marlena Plagianos | [bfriedland@popcouncil.org](file:///C:\Users\cmorrison\AppData\Local\Microsoft\Windows\Temporary%20Internet%20Files\Content.Outlook\NPHYNQGN\bfriedland@popcouncil.org)  [mplagianos@popcouncil.org](mailto:nrutenberg@popcouncil.org) |
| 13. Uganda EDCTP Microbicide Feasibility Study* | N/A | Janet Seeley | [Janet.Seeley@LSHTM.ac.uk](mailto:Janet.Seeley@LSHTM.ac.uk)  [Janet.Seeley@mrcuganda.org](mailto:Janet.Seeley@mrcuganda.org) |
| 14. Tanzania EDCTP Microbicide Feasibility Study* | N/A | Saidi Kapiga | [Saidi.Kapiga@lshtm.ac.uk](mailto:Saidi.Kapiga@lshtm.ac.uk) |
| 15. Partners in Prevention Trial | [http://depts.washington.edu/uwicrc/research/studies/pip_transmission](http://depts.washington.edu/uwicrc/research/studies/pip_transmission.html) | Jared Baeten | [jbaeten@uw.edu](file:///C:\Users\cmorrison\AppData\Local\Microsoft\Windows\Temporary%20Internet%20Files\Content.Outlook\NPHYNQGN\jbaeten@uw.edu) |
| 16. MDP 301 Microbicide Trial | <http://www.mdp.mrc.ac.uk/> | Angela Crook | angela.crook@ucl.ac.uk |
| 17. CAPRISA 004 Trial | [http://www.caprisa.org](http://www.caprisa.org/) | Nigel Garrett | [Nigel.Garrett@caprisa.org](mailto:Anneke.grobler@caprisa.org) |
| 18. FEMPrEP Trial | <http://femprep.fhi360.org/> | Lut Van Damme  Jennifer Deese | Lut.VanDamme@gatesfoundation.org  [jdeese@fhi360.org](file:///C:\Users\tcolter\AppData\Local\Microsoft\Windows\Temporary%20Internet%20Files\Content.Outlook\56Z0X303\jdeese@fhi360.org) |

* The Tanzanian (Mwanza Intervention Trials Unit) and the Ugandan (MRC/UVRI Uganda Research Unit) data may be made available on request as per the respective organizations’ Data Sharing Policy guidelines; interested parties must submit a Data Access Application form to the Data Access Committee. For studies 5, 6, and 14, requests should be made to Saidi Kapiga (Chair of the committee).  For study 13, requests should be made to Janet Seeley (Programme Head for this project).
