# Supplementary material for: Prevalence of sexually transmitted infections and bacterial vaginosis among women in sub-Saharan Africa: An individual participant data meta-analysis of 18 HIV prevention studies
Source: PLoS Med. 2018 Feb 27;15(2):e1002511. doi: 10.1371/journal.pmed.1002511 (PMC5828349; doi:10.1371/journal.pmed.1002511)
Supplement: S3 Text — (DOCX) [file pmed.1002511.s025.docx]

**Sexually Transmitted Infections among Women in Sub-Saharan Africa: Individual Participant Data Meta-Analysis of Prospective Studies**

**Funded by:** World Health Organization (WHO), Human Reproduction Programme

**Principal Investigators:** Elizabeth Torrone (Centers for Disease Control and Prevention)

Sami Gottlieb (WHO)

Charles Morrison (FHI 360)

**Primary analysis team:** Joelle Brown (University of California at San Francisco)

Nathalie Broutet (WHO)

Pai Lien Chen (FHI 360)

Jennifer Deese (FHI 360)

Suzanna Francis (London School of Hygiene and Tropical Medicine)

Ndema Habib (WHO)

Richard Hayes (LSHTM)

Cynthia Kwok (FHI 360)

Katharine Looker (University of Bristol)

Nicola Low (University of Bern)

Nuala McGrath (University of Southampton)

Vittal Mogasale (International Vaccine Institute)

Janneke van de Wijgert (University of Liverpool, University Medical Center Utrecht)

**Additional collaborators**: Jared Baeten, Angela Crook, Sinead Delany-Moretlwe, Barbara Friedland, Nigel Garrett, Renée Heffron, Anatoli Kamali, Saidi Kapiga, Rupert Kaul, Kelly Leask, Sue Mavedzenge, Scott McClelland, Sheena McCormack, Landon Myer, Marlena Plagianos, Helen Rees, Janet Seeley, Melanie Taylor, Lut Van Damme, Ariane Van der Straten, Deborah Watson-Jones

**Sexually Transmitted Infections among Women in Sub-Saharan Africa: Individual Participant Data Meta-Analysis of Prospective Studies**

**TABLE OF CONTENTS**

**STUDY TEAM**  1

**STUDY SUMMARY** 3

**LIST OF ABBREVIATIONS AND ACRONYMS** 4

**1 BACKGROUND** 5

**2 OBJECTIVES AND HYPOTHESES** 5

**3 METHODS**…… 6

3.1 Overview of studies … 6

3.2 Inclusion and exclusion criteria … 7

3.3 Selection of STIs/BV … 8

3.4 Definition of prevalence and incidence … 8

3.5 Censoring for incidence calculation … 9

3.6 Stratification of prevalence and incidence estimates… 9

3.7 Estimation of STI co-infection………………………….………….……….. 10

3.88 Assessment of the risk of bias in individual studies…………….……….. 10

4 **DATA MANAGEMENT** 10

­

**5 ANALYSIS** 11

5.1 Descriptive Analysis 11

5.2 Meta-analysis 11

5.3 Study Limitations and Sensitivity Analyses 12

**6 REFERENCES** 14

**APPENDICES**

Table 1. Studies in HC-HIV meta-analysis dataset 17

Table 2. Inclusion/exclusion criteria for HC-HIV meta-analysis dataset……….. 21

Table 3. Available STI/BV data 22

Table 4. Categorization of syphilis results…..………………………………………26

Table 5. Planned tables and forest plots……………..…………………………..…27

**SUPPLEMENTAL Tables**

Supplement Table 1. Proposed stratifications for prevalence estimates

Supplement Table 2. Proposed stratifications for incidence estimates

Supplement Table 3. Proposed stratifications for STI co-infection estimates

**Sexually Transmitted Infections among Women in Sub-Saharan Africa: Individual Participant Data Meta-Analysis of Prospective Studies**

**STUDY SUMMARY**

**Purpose:** To conduct an individual participant data (IPD) meta-analysis of prospective HIV prevention studies to evaluate prevalence and incidence of sexually transmitted infections (STIs) and bacterial vaginosis (BV) among women in sub-Saharan Africa

**Design:** Meta-analysis of individual participant data from 18 prospective HIV prevention studies using both one- and two-stage meta-analysis approaches.

**Study Population:** Approximately 37,000 women from clinic and community populations and groups of women at high-risk of HIV infection enrolled in selected prospective studies in sub-Saharan Africa.

**Primary Objectives:** 1) To evaluate the prevalence of selected

STIs/BV among women in sub-Saharan Africa by region, population type, and other pre-specified characteristics.

2) To evaluate the incidence of selected

STIs among women in sub-Saharan Africa by region, population type, and other pre-specified characteristics.

**Secondary Objectives:** 1) To evaluate the prevalence and incidence of co-

infections among the STIs/BV.

**LIST OF ABBREVIATIONS AND ACRONYMS**

BV bacterial vaginosis

CDC Centers for Disease Control and Prevention

EDCTP European and Developing Countries Clinical Trials Partnership

EIA enzyme immunoassay

HC-HIV Hormonal Contraception and Risk of HIV Acquisition

HIV human immunodeficiency virus

HPV human papillomavirus

HSV-2 herpes simplex virus type 2

IPD individual-participant data

RCT randomized controlled trial

RPR rapid plasma reagin

STI sexually transmitted infection

STROBE strengthening the reporting of observational studies in epidemiology

TPHA *Treponema pallidum* hemagglutination assay

TPPA *Treponema pallidum* particle agglutination assay

VPRP Vaginal Practices Research Partnership

WHO World Health Organization

**1. BACKGROUND**

Sexually transmitted infections (STIs) and bacterial vaginosis (BV) are widespread globally. These conditions result in a range of sexual, reproductive and maternal-child health consequences, including genital symptoms, pregnancy complications, infertility, enhanced HIV transmission, anogenital cancers, and important psychosocial consequences. The World Health Organization (WHO) estimates that 357 million new curable STIs (gonorrhea, chlamydia, syphilis, and trichomoniasis) occurred globally [2] and 417 million people had herpes simplex virus type 2 (HSV-2) infection during 2012 [3]. However, the paucity of high-quality published STI prevalence studies, especially among general populations and for low-income countries, including the lack of data from most African countries, has limited the accuracy of the estimation process. When studies do exist, they are often small and involve different analytic methods, making their use problematic for summary estimates. Global estimates of BV occurrence are not available.

In 2013, WHO held a technical consultation on accelerating development of effective new STI vaccines, which resulted in a global roadmap for STI vaccine development [4]. The roadmap outlines the critical need for better data on the burden of STI infection in low-income countries to more accurately assess the potential impact of STI vaccines, to stimulate investment in vaccine development, and to guide future vaccine implementation and evaluation. In addition, global STI management guidelines are currently being updated [5], and key decisions about the use of syndromic management (the use of presumptive treatment for symptomatic people without the use of laboratory tests) are dependent on accurate STI and BV prevalence data in different settings. Another consultation was held in 2013 on Methods for Improved Global STI Estimates. Technical experts from this meeting also identified the need for additional estimates and highlighted the importance of exploring potential data available through clinical research trials networks to improve estimates.

In 2014, in collaboration with investigators from multiple prospective studies, FHI360 Clinical Sciences and Biostatistics Departments led an individual participant data (IPD) meta-analysis of the association between use of hormonal contraception and HIV acquisition. [6] Researchers identified 18 randomized controlled trials of non-contraceptive HIV prevention interventions and cohort studies that contained prospectively-collected data on both hormonal contraceptive use and incident HIV-1 infections [7-24]. With permission of the principal investigators of each study, individual level data from each study were obtained and combined for the Hormonal Contraception and Risk of HIV Acquisition (HC-HIV) individual participant data (IPD) meta-analysis study. Many of these studies also captured information on STI/BV prevalence at baseline and acquisition throughout the course of the study. Although these studies were not specifically identified and combined to investigate STIs/BV, the HC-HIV meta-analysis dataset presents a unique opportunity to explore estimates of STI/BV prevalence and incidence among women in sub-Saharan Africa.

**2. OBJECTIVES**

Using individual level data from the 18 prospective, longitudinal studies included in the HC-HIV meta-analysis dataset, we will estimate the prevalence and incidence of selected STIs/BV among women in sub-Saharan Africa and investigate potential differences by region, population type, and demographic characteristics.

Specifically, we will investigate the following objectives:

1. To evaluate the prevalence of selected STIs (chlamydia, gonorrhea, trichomoniasis, syphilis, and HSV-2) and BV among women in sub-Saharan Africa by region, population type, time period, age, and pregnancy status.
   1. To evaluate the prevalence of co-infections among the STIs/BV
2. To evaluate the incidence of selected STIs (chlamydia, gonorrhea, syphilis, trichomoniasis,and HSV-2) among women in sub-Saharan Africa by region, population type, time period, and age.
   1. To evaluate the incidence of co-infections among the STIs

The term “STIs/BV” may refer to up to 6 conditions: gonorrhea, chlamydia, syphilis, trichomoniasis, HSV-2, and/or BV. For each objective, the precise conditions included were determined by the biological/ epidemiological basis for the particular research question, the availability of STI/BV-specific data at different time points, the validity of combining test results across studies or of calculating incidence for a given condition, and other methodological and biological considerations. See Section 3.3 for more details. Human papillomavirus (HPV) is not included as the majority of studies did not include HPV data.

**3. METHODS**

**3.1. Overview of Studies**

The HC-HIV meta-analysis dataset contains 18 randomized controlled trials of non-contraceptive HIV prevention interventions and cohort studies that contained prospectively-collected data on both hormonal contraceptive use and incident HIV-1 infections. Only studies from sub-Saharan Africa (where most studies have been conducted) were included. The HC-HIV meta-analysis dataset includes data from the 10 studies in the Vaginal Practices Research Partnership (VPRP), a partnership among numerous research groups with longitudinal datasets to investigate a possible association between various vaginal practices and HIV acquisition, and 8 additional studies (Appendix Table 1). All included studies met specific inclusion and exclusion criteria (Appendix Table 2).

The included studies in the HC-HIV meta-analysis dataset were not specifically selected to answer the objectives of this STI/BV prevalence and incidence study and there may be additional studies that could be included (e.g., other cross-sectional or longitudinal studies that include STI/BV data). However, the HC-HIV meta-analysis dataset was previously created in collaboration with study PIs and includes key variables of interest for this study, including STI/BV data, maximizing the utilization of the HC-HIV meta-analysis dataset as well as the efficiency of this analysis. Nonetheless, for the STI/BV analysis, the HC-HIV meta-analysis dataset should be considered a convenience sample of studies.

**3.1.2 Study participants**

The HC-HIV meta-analysis dataset includes study populations where heterosexual transmission is thought to be the predominant mode of HIV transmission. Studies include both women from the general population and groups of women identified at high-risk of HIV infection such as women who engage in transactional sex and HIV-negative women who are part of HIV-discordant couples. Only women from Sub-Saharan Africa are included. If studies included sites outside of Sub-Saharan Africa, only women from the Sub-Saharan African sites were included in the HC-HIV meta-analysis dataset. For incident analyses in the STI/BV meta-analysis, only women in intervention arms which did not include an intervention likely to impact STI incidence will be included. (See Section 3.2)

**3.2. Inclusion and exclusion criteria of studies in the HC-HIV meta-analysis dataset for STI/BV meta-analysis**

The HC-HIV meta-analysis dataset contains data from 18 studies. For each objective, studies in the HC-HIV meta-analysis dataset will be included in the STI/BV analysis based on study design, population, and data availability for each study.

Within each objective, inclusion and exclusion criteria will be applied to each STI/BV separately.

Objective 1: To evaluate prevalence of chlamydia, gonorrhea, trichomoniasis, syphilis, HSV-2, and BV among women in sub-Saharan Africa by region, population type, age, pregnancy status, and time period.

The following types of studies in the HC-HIV meta-analysis dataset will be included:

- Measured the STI/BV at baseline as part of protocol
- Indicates laboratory test used for the diagnosis of the STI/BV
- Includes test result for diagnosis of the STI/BV
- Tested at least 80% of all participants in the study for the STI/BV or tested at least 80% of randomly selected sample of enrolled participants
- Measured important covariates

The following types of studies in the HC-HIV meta-analysis dataset will be excluded:

- Where the study design required the STI/BV diagnosis in question for study inclusion
- If >10% of those tested for the STI/BV had indeterminate test results

Objective 2: To evaluate incidence of chlamydia, gonorrhea, syphilis, trichomoniasis, and HSV-2 among women in sub-Saharan Africa by region, population type, age, pregnancy status, and time period

The following types of studies in the HC-HIV meta-analysis dataset will be included:

- Measured the STI at two or more study visits as part of protocol
  - - Tested for chlamydia, gonorrhea, trichomoniasis, or syphilis at least every 6 months
    - Tested for HSV-2 at least every 12 months or for studies that tested at baseline and end of the study, also had retrospective interim testing at least every 12 months for participants that seroconverted at study end.
- Includes variable to indicate laboratory test used for the diagnosis of the STI
- Includes a population or sub-population that did not receive a STI prevention intervention
  - - - If the intervention in the intervention arm does not have a demonstrated or plausible chance of impact on STI incidence, participants in the intervention arm will be included.
- Protocol indicated testing all participants for STI at specific visits
  - - - Tested at least 80% of enrolled participants for the STI at follow-up or at least 80% of randomly selected participants as per protocol at follow-up
- Measured important covariates

The following types of studies in the HC-HIV meta-analysis dataset will be excluded:

- Where the study design required the STI diagnosis in question for study inclusion
- Where all participants received an STI prevention intervention likely to have an impact on STI incidence
- Where only symptomatic patients were tested on follow-up visits
- If >10% of those tested for the STI had indeterminate test results

**3.3. Selection of STIs/BV for each objective**

See Appendix Table 3 for available STI/BV data in selected studies.

For objective 1, prevalence will be estimated for six STIs/BV (chlamydia, gonorrhea, syphilis, trichomoniasis, BV, and HSV-2) based on availability of data. For the majority of studies, HPV data were not available.

For objective 2, incidence will be estimated for five of the selected STIs (chlamydia, gonorrhea, syphilis, trichomoniasis, and HSV-2). BV was not included due to concerns about the accuracy of diagnostic criteria to assess incidence given the intervals between follow-up tests.

**3.4. Definition of prevalence and incidence**

Prevalent STI/BV infections will be defined by diagnosis at the baseline visit. Infections will be coded as positive, negative, or missing/not classifiable/not collected or asked. Positive syphilis cases will be further categorized based on rapid plasma regain (RPR) titer testing as high titer active infection, low titer infection or unknown RPR titer infection.

Infections defined as:

Chlamydia: Positive: Positive nucleic acid amplification test (NAAT), enzyme immunoassay (EIA), or hybrid capture test

Gonorrhea: Positive: Positive NAAT, or culture

Trichomoniasis: Positive: Positive NAAT, InPouch culture, or wet mount

HSV-2: Positive: Positive type-specific serologic test

BV: Positive: Nugent score (≥7), Ison-Hay grade (≥Grade III), or Amsel criteria (≥ 3 criteria)

Syphilis (see Appendix table 4):

Positive (overall): positive RPR AND positive treponemal test (*Treponema pallidum* hemagglutination assay [TPHA], *Treponema pallidum* particle agglutination assay [TPPA] or Determine TP rapid test)

With subclassification based on RPR titer testing:

Positive, high titer active infection: positive RPR with a titer ≥1:8 AND positive treponemal test

Positive, low titer infection: 1) positive RPR with a titer <1:8 AND positive treponemal test

Positive, unknown RPR titer infection: 1) positive RPR with missing titer AND positive treponemal test

Negative: 1) negative RPR or 2) positive or missing RPR AND negative treponemal test

Missing: 1) positive RPR AND missing treponemal test or 2) missing RPR AND missing or positive treponemal test

For estimation of incident infections, individual study protocols will be reviewed to assess time frames of repeat STI testing, types of laboratory tests used, treatment information for curable STIs. For chlamydia, gonorrhea, trichomoniasis, and HSV-2, incident infection will be defined as the first positive laboratory test following a previously negative result for the infection. For trichomoniasis, incidence estimations will be limited to those studies where *T. vaginalis* testing was done using InPouch or NAAT. For syphilis incidence, using the categories defined above, an incident infection will be defined as:

1. participants who are negative at baseline who are diagnosed at a follow-up visit with a positive infection OR
2. participants who had a positive, low titer infection at baseline who are diagnosed at follow-up with a positive, high titer active infection with a 4-fold increase in titer.

For all analyses, time of infection will be estimated as the midpoint between the last negative test and the first positive test.

**3.5. Censoring for incidence calculation**

Study participants will be censored at the first time they are documented as diagnosed with selected STI or at the end of the study or at the last follow-up visit where STI testing was done. If resources allow and data are available/complete, a sensitivity analysis will be conducted to estimate incidence of recurrent STIs; analyses will apply appropriate statistical methods to account for non-independence of subject-specific observations.

**3.6. Stratification of prevalence and incidence estimates**

Because of possible heterogeneity in STI/BV prevalence and incidence among studies, for each STI/BV we plan to stratify results according to pre-specified characteristics. (See Supplemental Tables 1.1 – 1.2 for proposed stratifications for prevalence estimates and Supplemental Tables 2.1 – 2.2 for proposed stratifications of incidence estimates.) We will first stratify according to the study-level characteristics for region and population type. Within these strata (region and population type), we will then explore STI/BV prevalence and STI incidence according to individual-level characteristics, including: age, time period of enrolment, and pregnancy status (prevalence estimates only). Additional stratifications, such as by sexual behaviors including number of recent sexual partners, could be explored if resources allow. Additionally, diagnostic test used can influence observed prevalence. For BV, those diagnosed by Nugent or Ison-Hay criteria will be evaluated separately from those diagnosed by Amsel criteria only. A sensitivity analysis will be conducted to determine if the estimates remain robust if Amsel only results are included with the Nugent/Ison-Hay results (see section 5.3). For chlamydia, gonorrhea, syphilis and trichomoniasis, estimates will be evaluated by diagnostic test type to investigate robustness of estimates (see section 5.3).

Region (based on geographical location of the study) will be defined as South Africa, Eastern Africa (Kenya, Uganda, Tanzania, Rwanda), or Southern Africa (Zambia, Zimbabwe, Malawi, Botswana). Population types (based on overall study population enrolled) will be defined as higher-risk or clinic/community-based populations. Higher-risk populations include studies where participants were recruited from bars or other recreational facilities like guesthouses and hotels, or were women who engage in transactional sex. Clinic/community-based populations include participants recruited in a clinic setting, such as a family planning/reproductive health clinic, as well as studies where participants were recruited from the general population or community. Some studies in the HC-HIV meta-analytic dataset recruited HIV-negative partners in HIV-discordant couples. Although these participants may be at higher risk for HIV transmission than the general population, their risk for STI/BV may be similar to clinic/community-based populations given that they are in a stable relationship. These studies will initially be analyzed separately, STI/BV specific estimates reviewed, and if appropriate (based on comparison with estimates from other populations), will be included in either the higher-risk strata or the clinic-based strata. If not appropriate, estimates from these studies will be presented separately. See Appendix Table 1 for proposed classifications of studies according to these two characteristics.

Time period, age, and pregnancy status will be based on individual participant characteristics. Time period will be categorized as participants whose baseline data were collected prior to 2002 (“earliest” time period), participants whose baseline data were collected during 2003 to 2006 (“later” time period), and participants whose baseline data were collected in 2007 or later (“latest” time period). The categorization of time periods may be adjusted if necessary based on available sample size. Age will be categorized as 15-49 years, 15-24 years, 25-49 years, and if possible given sample size, 15-19 years, and 20-24 years. The categorization of age may be adjusted if necessary based on available sample size. Pregnancy status will be based on pregnancy status at baseline, either self-reported or by pregnancy test.

To the extent possible given sample size, prevalence and incidence estimates will be cross-stratified by various characteristics.

**3.7 Estimation of STI co-infection**

To broadly evaluate the prevalence and incidence of STI co-infection for the STIs/BV under consideration, the percentage of participants co-infected with two STIs/BV will be estimated. STI co-infection estimates will be estimated by population type (based on overall study population enrolled) defined as higher-risk or clinic/community-based populations and within population type by age group. For co-infection estimates, the denominators will be limited to those participants who were tested for the two STIs under consideration. See Supplemental Table 3. If resources allow, additional co-infection analyses will estimate the number of STIs/BV diagnoses received by participants (e.g., 0, 1, 1-2, 3+).

**3.8. Assessment of the risk of bias in individual studies**

All studies included in the HC-HIV meta-analysis dataset are cohort studies or randomized trials.

Knowledge of methodological features that can bias study results is important for interpreting the findings from individual studies and how the differences between studies might contribute to heterogeneity in study results. Unlike meta-analyses based on aggregated data, which generally rely only on information within published papers, an in-depth assessment of the risk of bias in individual studies will be possible. The assessment will make use of the individual participant data, study protocols, clinical study reports, available publications and communications with the study investigators. Additional factors can be assessed, such as whether those lost to follow-up were systematically different to those who remained in the study.

This process has already been completed for the HC-HIV IPD meta-analysis but will be revisited to include issues related to the assessment of STI prevalence and incidence. Using the study protocol, published articles and data sent by the studies, two reviewers will assess independently the following items (no score will be used) that has been adapted from the Strengthening the Reporting of Observational Studies (STROBE) statement and checklist:

For objectives 1 and 2:

1. How much data on STIs/BV are missing?
2. Were important covariates measured in the study?
3. Were appropriate STI/BV diagnostic tests used?

For objective 2:

1. Was the level of retention high in the study (>80% at 12 months)?
2. How frequently were STIs measured?

**4. DATA MANAGEMENT**

The HC-HIV meta-analysis dataset will be used as the base for this STI meta-analysis. This dataset was established in accordance with ethics guidelines, and the Protection of Human Subjects Committee of FHI 360 approved the study and judged it as exempt research (PHSC #10263). All included studies had relevant country-specific institutional ethical review and regulatory board approvals, and all participants within each study provided written informed consent for study participation. No information that could identify an individual woman was obtained in establishing the HC-HIV dataset. A unique number was assigned to each study and each woman. A list with the HC-HIV IPD meta-analysis identification numbers and the study identification numbers from the original 18 studies was retained in case clarification about specific variables was needed.

To create the final analytic dataset for the STI analysis, any additional variables needed will be requested through a data format sheet. The data format sheet includes the instructions for numerical coding, with the question number to which each variable relates and the required format. The study/data manager from any of the original 18 studies from which data are needed will send the data format sheet and the corresponding data to a designated biostatistician at FHI360. Variables from each study will be recorded if necessary, using pre-specified categories. The categories will depend on the consistency of coding between studies. Once the final analytic dataset for the STI analysis is completed, the link between the STI meta-analysis identification numbers and the HC-HIV dataset numbers will be destroyed. All the data will be stored securely on the access required storage drive.

**5. ANALYSIS**

The following sections and Table 1 describe the statistical methods that will be used to investigate the objectives and hypotheses in Section 2. We will use descriptive statistics and statistical tests to examine between-study heterogeneity and univariable and multivariable methods for the analysis of IPD. Table 5 provides a summary of the planned forest plots and tables.

**5.1 Descriptive Analysis**

We will conduct descriptive analyses before proceeding to meta-analyses in order to gain a detailed understanding of the data received within and between studies. Descriptive statistics will be used to summarize study participant characteristics (i.e. socio-demographic characteristics, risk behaviors, and study duration, etc.) by each study and overall.

Categorical variables or continuous variables that have been categorized will be summarized by frequencies and percentages and analyzed using Cochran-Mantel-Haenszel tests across study groups and sites. Data recorded for continuous variables will be summarized by medians and ranges and analyzed by Wilcoxon Mann Whitney tests among the study groups and/or sites.

**5.2. Meta-analysis**

We will conduct meta-analysis of individual participant data using both one- and two-stage meta-analysis approaches [25]. Each method has advantages and disadvantages. When both methods are used, they can provide valuable complementary information.

**5.2.1 Two-stage meta-analysis**

We will use two-stage meta-analysis as our primary approach to examine prevalence and incidence estimates. Using this method, participants in each study are compared directly only with other participants in the same study. The two-stage method is well suited to assess between study heterogeneity. While the method is less suitable for identifying prognostic factors (due to the different sets of covariates measured across datasets), it is possible to assess effect modification.

In the first stage, IPD in each study are analyzed separately to obtain summary statistics. We will use binomial regression models to estimate prevalence estimates for each selected STI/BV and Poisson regression to estimate the incidence for each selected STI.

If appropriate, the second stage is to use standard meta-analysis techniques to combine the summary measures to give an overall estimate of prevalence and incidence across studies by population-type. The decision to combine results and conduct further analyses using random and/or fixed effect models depends on the level of between-study heterogeneity.

The same approach will be used to estimate prevalence and incidence for each stratum of interest based on region, population type, age, pregnancy status, and time period of the study.

**5.2.1.a Heterogeneity Assessment**

Marked heterogeneity between studies may make it inappropriate to calculate overall summary measures of prevalence and incidence for each STI/BV, but exploring this heterogeneity can provide valuable insights. First, we will examine between-study heterogeneity in estimates of prevalence and incidence visually using forest plots of the summary estimates. We will use two statistics to measure the degree of heterogeneity in this meta-analysis: 1) the Q-statistic for which a p-value <0.10 will be interpreted as statistical evidence of heterogeneity (exceeding what would be expected by chance); 2) the I^2^ statistic and its 95% confidence interval, which describes the percentage of total variation across studies due to heterogeneity other than chance [26].

We will use the I^2^ statistic to classify the degree of between-study heterogeneity into low heterogeneity (I^2^ <50%), mild or moderate heterogeneity (I^2^ between 50-75%), and high heterogeneity (I^2^ > 75%). If we find low between-study heterogeneity, no further investigation of heterogeneity will be done. Otherwise, we will examine potential reasons for between-study heterogeneity using stratification or meta-regression. The outcome of this exploration of heterogeneity will determine decisions about the appropriateness of meta-analysis to pool the prevalence and incidence estimates from the component studies. If the decision is to pool the estimates, random-effect models will be used to incorporate the heterogeneity.

**5.2.2 One-stage meta-analysis**

The one-stage method combines and analyzes data from all studies as if they belong to a single study. Study identity is included in statistical models to take into account the fact that the data are from different studies. This can be seen as a multilevel model, with two levels, which allows the estimation of effects of interest in relation to both study-level and patient-level covariates. The one-stage method might not be a valid option if there is marked heterogeneity between the studies.

We will use stratified binomial regression model to examine overall prevalence estimates. We will use stratified Poisson regression to examine the overall incidence estimates. The impact of the over dispersion effects on the pooled prevalence and incidence estimates will also be evaluated.

**5.3. Study limitations and sensitivity analyses**

Though this meta-analysis provides high quality individual participant data and valuable information about the prevalence and incidence of selected STIs/BV, our approach has some limitations. First, the validity of the results of the meta-analysis is dependent on the quality of the individual component studies. While the IPD meta-analysis can help avoid problems associated with the analyses and reporting of the component studies, it cannot eliminate bias due to their study design or conduct. For example, studies which recruited high-risk women will likely have higher incidence estimates. In addition, due to the variety of study designs, not all stratification subgroups may be available in all component studies. Second, as the HC-HIV meta-analysis dataset is a convenience sample of prospective longitudinal studies that were identified for an analysis of hormonal contraception and the risk of HIV acquisition, the studies included may not be representative of all populations in the regions of interest for the STI/BV analysis. Finally, it is also possible that we may be unable to recode variables appropriately across studies and may not be able to investigate all sub-classifications of interest.

We will perform the following sensitivity analyses to examine the robustness of the results of our meta-analyses:

1. Based on the type of diagnostic criteria – stratify and analyze data/studies by the following criteria :
2. For chlamydia and gonorrhea: any test vs. NAAT only
3. For BV: any test vs. Nugent/Ison-Hay only
4. Based on the assessment of the risk of bias of the component study – stratify studies with and without an identified risk of bias. Suggested stratification for incidence estimates: studies that have retention rates <80% at one year vs. studies that have ≥80% retention
5. Based on intervention arm – repeat incidence analysis excluding all intervention arms.
6. Based on length of follow-up – for calculation of incidence, stratify studies with short versus longer follow-up time periods. Suggested stratification: ≤3 months vs 3 or more months followup
7. Based on study inclusion criteria – remove studies that included diagnosis with a specific STI for study inclusion for investigation of the prevalence and incidence of other STIs (if resources allow)
8. Based on censoring criteria – for calculation of incidence do not censor at first diagnosis of STI in question (if resources allow)

Finally, in consideration of the described potential limitations of our study, we will interpret the results of the meta-analyses cautiously.

1. **REFERENCES**
2. Low N, Chersich MF, Schmidlin K et al. Intravaginal Practices, Bacterial Vaginosis, and HIV Infection in women: Individual Participant Data Meta-analysis. PLoS Med 2011; 8(2): e1000416. Doi:10.1371/journal.pmed.1000416.

Newman L, Rowley J, Vander Hoorn S, et al. Global estimates of the prevalence and incidence of four curable sexually transmitted infections in 2012 based on systematic review and global reporting. PLoS One. 2015;10(12):e0143304.

Looker KJ, Magaret AS, Turner KM, Vickerman P, Gottlieb SL, Newman LM. Global estimates of prevalent and incident herpes simplex virus type 2 infections in 2012. PLoS One 2015; 10(1):e114989.

Broutet N, Fruth U, Deal C, Gottlieb SL, Rees H; participants of the 2013 STI Vaccine Technical Consultation. Vaccines against sexually transmitted infections: The way forward. Vaccine 2014; 32(14):1630-7.

1. WHO. Guidelines for the management of sexually transmitted infections. Geneva: WHO; 2004, Available at: <http://www.who.int/hiv/pub/sti/pub6/en/>
2. Morrison CS, Chen PL, Kwok C, et al Hormonal contraception and the risk of HIV acquisition: an individual participant data meta-analysis. PLoS Med. 2015 Jan 22;12(1):e1001778. doi: 10.1371/journal.pmed.1001778.
3. Low N, Chersich MF, Schmidlin K et al. Intravaginal Practices, Bacterial Vaginosis, and HIV Infection in women: Individual Participant Data Meta-analysis. PLoS Med 2011; 8(2): e1000416. Doi:10.1371/journal.pmed.1000416.
4. Ungchusak K, Rehle T, Thammapronpilap P et al. Determinant of HIV infection among female commercial sex workers in northeastern Thailand: results from a longitudinal study. J Acquir Immune Defic Syndr Hum Retrovirol 1996; 12: 500-507.
5. Kumwenda JJ, Makanani B, Taulo F et al. Natural history and risk factors associated with early and established HIV type 1 infection among reproductive-age women in Malawi. CID 2008; 46: 1913-1920.
6. Feldblum PJ, Lie CC, Weaver MA et al. Baseline factors associated with incident HIV and STI in four microbicide trials. Sexually Transmitted Diseases 2010; 37: 594-601.
7. Heffron R, Donnell D, Rees H et al. for the Partners in Prevention HSV/HIV Transmission Study Team. Use of hormonal contraceptives and risk of HIV-1 transmission: a prospective cohort study. The Lancet Infectious Diseases 2011; published online Oct 4, 2011. DOI:10:1016/S1473-3099(11)70247-X.
8. Bultreys M, Chao A, Habimana P et al. Incident HIV-1 infection in a cohort of young women in Butare, Rwanda. AIDS 1994; 8: 1585-1591.
9. Kleinschmidt I, Rees H, Delany S et al. Injectable progestin contraceptive use and risk of HIV infection in a South African family planning cohort. Contraception 2007; 75: 461-467.
10. Baeten JM, Benki S, Chohan V et al. Hormonal contraceptive use, herpes simplex virus infection, and risk of HIV-1 acquisition among Kenyan women. AIDS 2007; 21: 1771-1777.
11. Watson-Jones D, Baisley K, Weiss HA et al. Risk factors for HIV incidence in women participating in an HSV suppressive treatment trial in Tanzania. AIDS 2009; 23: 415-422.
12. Kilmarx PH, Limpakarnjanarat K, Mastro TD et al. HIV-1 seroconversion in a prospective study of female sex workers in northern Thailand: continued high incidence among brothel-based women. AIDS 1998; 12: 1889-1898.
13. Morrison CS, Chen PL, Kwok C, et al. Hormonal contraception and HIV acquisition: reanalysis using marginal structural modeling. AIDS 2010; 24: 1778-1781.
14. Morrison CS, Skoler-Karpoff S, Kowk C, et al. Hormonal Contraception and the risk of HIV acquisition among women in South Africa. AIDS 2012; 26: 497-504.
15. Wand H and Ramjee G. The effects of injectable hormonal contraceptives on HIV seroconversion and on sexually transmitted infections. AIDS 2012; 26: 375-380.
16. Myer L, Denny L, Wright TC et al. Prospective study of hormonal contraception and women’s risk of HIV infection in South Africa. International Journal of Epidemiology 2007; 36: 166-174.
17. Reid SE, Dai JY, Wang J et al. Pregnancy, contraceptive use, and HIV acquisition in HPTN 039: relevance for HIV prevention trials among African women. JAIDS 2010; 53: 606-613.
18. Kiddugavu M, Makumbi F, Wawer M et al. Hormonal contraceptive use and HIV-1 infection in a population-based cohort in Rakai, Uganda. AIDS 2003; 17: 233-240.
19. Kapiga SH, Lyamuya EF, Lwihula GK et al. The incidence of HIV infection among women using family planning methods in Dar es Salaam, Tanzania. AIDS 1998; 12: 75-84.
20. Morrsion CS, Richardson BA, Mmiro F, et al. Hormonal contraception and the risk of HIV acquisition. AIDS 2007; 21: 85-95.
21. Bowden J, Tierney JF, Simmonds M, et al. Individual patient data meta-analysis of time-to-event outcomes: one-stage versus two-stage approaches for estimating the hazard ratio under a random effects model. Research Synthesis Methods 2011; 2: 150–162. doi: 10.1002/jrsm.45.
22. Higgins J, Thompson S. Quantifying heterogeneity in a meta-analysis. Statistics in Medicine 2002; 21(11):1539-58.

**Table 1. Studies included in the HC-HIV meta-analysis dataset**

| Study  # | PI | Country | Region category | Study Population | Study population category | Study design/objective | Ages included | Included  *N* |
| --- | --- | --- | --- | --- | --- | --- | --- | --- |
| 1 | McClelland | Kenya (Mombasa) | Eastern Africa | Women who engage in transactional sex | High-risk | Cohort: hormonal contraception and HIV | 16-48 | 1,270 |
| 2 | Myer | South Africa | South Africa | Women not screened for cervical cancer | Clinic/  community-based  population | RCT: cervical cancer screening interventions | 35-49 | 4,160 |
| 3a | Morrison | Uganda | Eastern Africa | Women attending RH clinics | Clinic/  community-based  population | Cohort: hormonal contraception and HIV | 18-35 | 2,201 |
| 3b | Morrison | Zimbabwe | Southern  Africa | Women attending RH clinics | Clinic/  community-based  population | Cohort: hormonal contraception and HIV | 18-35 | 2,248 |
| 4 | Kaul | Kenya | Eastern  Africa | Women who engage in transactional sex | High-risk | RCT: presumptive antibiotic treatment | 18-49 | 414 |
| 5 | Francis | Tanzania | Eastern  Africa | Women working in bars | High-risk | Cohort: microbicide feasibility study | 15-49 | 978 |
| 6 | Watson-Jones | Tanzania | Eastern  Africa | Women working in bars and other recreational facilities | High-risk | RCT: HSV suppression to reduce HIV transmission | 16-35 | 781 |
| 7a | van der Straten, Padian | Zimbabwe  (MIRA) | Southern  Africa | Sexually active women | Clinic/  community-based  population | RCT: diaphragm/ condoms to reduce HIV acquisition | 18-49 | 2,455 |
| 7b | van der Straten, Padian | South Africa  (MIRA) | South Africa | Sexually active women | Clinic/  community -based  population | RCT: diaphragm/ condoms to reduce HIV acquisition | 18-49 | 2,493 |
| 8 | Kleinschmidt, Rees | South Africa  (PALESA) | South Africa | Women attending RH clinics | Clinic/  community-based  population | Cohort: hormonal contraception and HIV | 18-41 | 551 |
| 9 | Delaney-Moretlwe | South Africa  (RHRU) | South Africa | Women attending clinics | Clinic/  community-based  population | Cohort: microbicide feasibility study | 18-35 | 694 |
| 10 | McGrath | South Africa | South Africa | Women attending clinics | Clinic/  community-based  population | Cohort: microbicide feasibility study | 15-49 | 261 |
| 11 | Kumwenda, Brown | Malawi/ Zimbabwe | Southern  Africa | Women attending clinics | Clinic/  community-based  population | Cohort: microbicide feasibility study | 18-49 | 993 |
| 12 | Skoler-Karpoff | South Africa  (Carraguard) | South Africa | Sexually active women | Clinic/  community-based  population | RCT: microbicide to reduce HIV acquisition | 16-49 | 5,567 |
| 13 | Hayes,  KamaliSeeley | Uganda | Eastern  Africa | Women who engage in transactional sex | High-risk | Cohort: microbicide feasibility study | 15-49 | 418 |
| 14 | Hayes, Francis | Tanzania | Eastern  Africa | High risk women | High-risk | Cohort: microbicide feasibility study | 18-44 | 873 |
| 15a | Heffron, Baeten | Kenya, Rwanda, Uganda  (partners in prev) | Eastern  Africa | Sexually active women whose partners had HIV and HSV-2 | HIV-discordant couples | RCT: HSV suppression to reduce HIV transmission | 18-49 | 913 |
| 15b | Heffron, Baeten | Botswana, Zambia  (partners in prev) | Southern Africa | Sexually active women whose partners had HIV and HSV-2 | HIV-discordant couples | RCT: HSV suppression to reduce HIV transmission | 18-49 | 214 |
| 15c | Heffron, Baeten | South Africa  (partners in prev) | South Africa | Sexually active women whose partners had HIV and HSV-2 | HIV-discordant couples | RCT: HSV suppression to reduce HIV transmission | 18-49 | 138 |
| 16a | McCormack | Tanzania  (MDP301) | Eastern  Africa | Women working in bars and other recreational facilities | High-risk | RCT: microbicide to reduce HIV acquisition | 16-49 | 1018 |
| 16b | McCormack | Uganda  (MDP301) | Eastern  Africa | Women in discordant partnerships | HIV-discordant couples | RCT: microbicide to reduce HIV acquisition | 16-49 | 774 |
| 16c | McCormack | Zambia  (MDP301) | Southern Africa | Sexually active women | Clinic/  community-based  population | RCT: microbicide to reduce HIV acquisition | 18-49 | 1150 |
| 16d | McCormack | South Africa  (MDP301) | South Africa | Sexually active women | Clinic/  community-based  population | RCT: microbicide to reduce HIV acquisition | 17-49 | 5654 |
| 17 | Salim Karim | South Africa (CAPRISA) | South Africa | Sexually active women | Clinic/  community-based  population | RCT: microbicide to reduce HIV acquisition | 18-40 | 444 |
| 18a | Van Damme | Kenya, Tanzania  (FEM-PrEP) | Eastern  Africa | Sexually active women | Clinic/  community-based  population | RCT: microbicide to reduce HIV acquisition | 18-35 | 380 |
| 18b | Van Damme | South Africa  (FEM-PrEP) | South Africa | Sexually active women | Clinic/  community-based  population | RCT: microbicide to reduce HIV acquisition | 18-35 | 639 |

**Table 2. Inclusion and exclusion criteria for HC-HIV meta-analysis dataset**

| Inclusion criteria |
| --- |
| Studies which:   - Measured HIV prospectively at multiple time points with a testing interval of 6 months or less - Measured HIV using a standardized testing algorithm; - Measured hormonal contraceptive use prospectively at multiple time points with a measurement interval of 6 month or less - Measured hormonal contraceptive use using a standardized questionnaire - Included women between the ages of 15-49 years - Included women who used injectable contraception - Included at least 15 incident HIV infections in the dataset - Measured important covariates including (at a minimum) age, condom use, and number of sexual partners. |
| Exclusion criteria |
| Studies which:   - Where either HIV infection or hormonal contraceptive use were not measured prospectively and at multiple time points or with a testing interval of more than 6 months; - Hormonal contraceptive use is not measured prospectively using standardized data collection forms or has a measurement interval of more than 6 months; - Where there is a significant amount (>5%) of missing HIV infection or hormonal contraceptive use data; - Studies with small numbers of women (or person-time) using hormonal contraception; - Studies where the frequency of scheduled follow-up visits is longer than 6 months apart; - For studies that had an intervention arm where anti-retroviral drugs were provided (for prevention of HIV infection), women assigned to the intervention arm were excluded |

|  |  | | Table 3. STI/BV data available in HC-HIV meta-analysis dataset, including diagnostic tests [NB: to be updated] | | | | | | | | | | | | | | | | | | | |
| --- | --- | --- | --- | --- | --- | --- | --- | --- | --- | --- | --- | --- | --- | --- | --- | --- | --- | --- | --- | --- | --- | --- |
|  | | **Chlamydia** | | | | **Gonorrhea** | | | **HSV-2** | | |  | | **Trichomoniasis** | | |  | **Syphilis** | | | **BV** | |
|  | | **Baseline** | | **Follow-up** | **Test type** | **Baseline** | **Follow-up** | **Test type** | **Baseline** | **Follow-up** | **Test type** | | **Baseline** | | **Follow-up** | **Test type** | **Baseline** | | **Follow-up** | **Test type** | **Baseline** | **Test type** |
| #1 (McClelland) | | Yes | | About 70% tested on every FU visit | EIA | Yes | Every FU visit | Culture | Yes | Yes including prevalent and incident HSV2 result | Focus EIA | | Yes | | Yes | Wet mount | Yes | | Some Fup visits | RPR/ TPHA | Yes | Nugent |
| #2 (Meyer) | | Yes | | No | Hybrid capture GC/CT DNA assay | Yes | No | Hybrid capture GC/CT DNA assay | No | No | N/A | | Yes | | Some  FU visits | Wet mount | No | | No | N/A | Yes (subset) | Amsel and Nugent |
| #3a/b (Morrison) | | Yes | | Every FU visit | NAAT | Yes | Every FU visit | NAAT | Yes | Yes including prevalent and incident HSV2 result | Focus  EIA | | Yes | | Yes | Wet mount | Yes | | Every 6 months | RPR/ TPPA/  TPHA | Yes | Amsel and Nugent |
| #4  (Kaul) | | Yes | | >50% tested on FU2 (6M) Fewer tested on other FU visits | PCR | Yes | >50% tested on FU2 (6Mt)  Fewer tested on other FU visits | Culture/  PCR | Yes | Yes, only prevalent HSV2 result | ?? | | Yes | |  | ?? | Yes | |  | RPR/ TPPA/TPHA | Yes | Nugent |
| #5 (Francis) | | Yes | | At 6 and 12 months | PCR | Yes | At 6 and 12 months | PCR | Yes | At baseline and Final visit | IgG Kalon | Yes | | | No | N/A | Yes | | Some  FU visits | RPR/ TPPA | Yes | Nugent |
| #6 (Watson-Jones) | | Yes | | At 6, 12, 24 and 30 months | PCR | Yes | At 6, 12, 24 and 30 months | PCR | Yes | Yes  Had to be HSV-2 positive at baseline to join trial | IgG Kalon | Yes | | | At baseline, 6, 12, 24 and 30 months | In pouch and wet mount | Yes | | At baseline, 6, 12, 18, 24 and 30 months | RPR/ TPPA/ | Yes | Nugentand Ison Hay at baseline; Ison Hay at Fup |
| #7a/b (van der Straten, Padien) | | Yes | | Every FU visit | PCR | Yes | Every FU visit | PCR | Yes | Yes including prevalent & incident HSV2 result | FocusELISA | Yes | | | Yes | PCR | Yes | | Final visit only | RPR/ TPHA | Yes, if symptomatic | Amsel |
| #8 (Kleinschmidt, Rees) | | Yes | | Every FU visit |  | Yes | Every FU visit | ?? | All ulcer+, random- | All ulcer+, random- | N/A | Yes | | |  | ?? | Yes | |  | TPPA/TPHA | Yes | Nugent |
| #9 (Delaney-Moretlwe) | | Yes | | >90% tested on FU2 (6M) & 4 (12M) Few tested on the other visits (max FU4) | ?? | Yes | >90% tested on FU2 (6M) & 4 (12M) Few tested on other visits (max FU4) | ?? | Yes | Yes including prevalent and incident HSV2 result |  | Yes | | |  | ?? | No Lab Form | |  | No Lab Form |  | Yes |
| #10 (McGrath) | | Yes | | No | ?? | Yes | No | ?? | Yes | Yes including prevalent and incident HSV2 result |  | Yes | | |  | ?? | No | |  | TPPA/TPHA | Yes | Nugent (only pos/neg) |
| #11 (Kumwenda, Brown) | | Yes | | Some  FU visits | ELISA | Yes | Some  FU visits | culture | Yes  Only Zim site | Some  FU visits | Focus ELISA | Yes | | | Some  FU visits | Wet mount | Yes | | Some  FU visits | RPR/ TPHA | Yes | Amsel |
| #12  (Skoler-Karpoff) | | Yes | | >90% tested on FU2 (3M), 3 (6M), 5 (12M), 7 (18M) & 9 (24M) (Fewer tested on other visits) | ?? | Yes | >90% tested on FU2 (3M), 3 (6M), 5 (12M), 7 (18M) & 9 (24M) (Fewer tested on other visits_ | ?? | No data available | No data available | N/A | Yes | | |  | ?? | Yes | |  | RPR/ TPPA/TPHA | Yes (only symptomatic) | Amsel |
| #13  (Hayes - UG) | | Yes | | Every FU visit | PCR | Yes | Every FU visit | PCR | Yes | Yes including prevalent & incident HSV2 result | KalonELISA | Yes | | | Yes | In Pouch PCR | Yes | | Yes | RPR/ TPHA | Yes | Nugent |
| #14  (Hayes - TZ) | | Yes | | Only tested on FU2 (6M) and FU4 (12M) (max FU4) | ?? | Yes | Only tested on FU2 (6M) and FU4 (12M) (max FU4) | ?? | Yes | Yes including prevalent & incident HSV2 result | ?? | Yes | | |  | ?? | Yes | |  | Yes | Yes | Nugent |
| #15a/b (Heffron, Baeten) | | Yes (15% didn’t test) | | No | ?? | Yes (15% didn’t test) | No | ?? | Yes | Yes, only prevalent HSV2 result | ?? | Yes (15% didn’t test) | | |  | ?? | Yes | |  | RPR/ TPPA/TPHA | Yes | Nugent |
| #16a/b (McCormack) | | Yes | | Some  FU visits | nucleic acid amplification assays (NAATs) | Yes | Some  FU visits | nucleic acid amplification assays (NAATs**)** | Yes | Yes including prevalent and incident HSV2 result | Focus/Kalon ELISA | Yes | | | Some Fup | In Pouch PCR | Yes | | Some Fup visits (per local practice) | RPR/ TPHA | Yes | Ison-hay |
| #17  (Salim Karim) | | Yes (subset) | | No | ?? | Yes (subset) | No | ?? | Only at baseline (but No data available) | Not done | N/A | Yes (subset) | | |  | ?? | No Lab Form | |  | N/A |  | No |
| #18a/b  (Van Damme) | | Yes | | Only with clinical indication | NAAT | Yes | Only with clinical indication | NAAT | Subset only (PretoriaPts with fup) | Subset only (PretoriaPts with fup) | Kalon/Focus ELISA | Yes | | | Only with clinical indication | Wet mount | Yes | | Only with clinical indication | RPR/ TPHA | Yes | Nugent |

**Table 4. Categorization of syphilis results**

| 0 | | RPR Results | | | | |
| --- | --- | --- | --- | --- | --- | --- |
|  |  | **Positive & titer ≥1:8** | **Positive &**  **titer <1:8** | **Positive &**  **missing titer** | **Negative** | **Missing** |
| Treponemal test Results | **Positive** | Positive, high titer active | Positive, low titer infection | Positive, unknown titer infection | Negative | Missing |
|  | **Negative** | Negative | Negative | Negative | Negative | Negative |
|  | **Missing** | Missing | Missing | Missing | Negative | Missing |

**Table 5. Planned tables and forest plots**

| **Analysis objective** |  | **Meta-analysis output/presentation** | | | |
| --- | --- | --- | --- | --- | --- |
|  | **Analysis population** | **Baseline table** | **Heterogeneity evaluation** | | **One-stage stratified analysis and two-stage random effect analysis** |
|  |  |  | **I^2^** | **Forest plot** |  |
| **Primary** |  |  |  |  |  |
| **To evaluate the prevalence of selected STIs/BV: gonorrhea, chlamydia, syphilis, trichomoniasis, HSV-2, and/or BV** | **All studies** | **√** | **√** | **√** | **√** |
|  | **Region**: South Africa, Eastern Africa, Southern Africa | **√** | **√** |  | **√** |
|  | **Population type**: higher-risk or clinic/community-based populations | **√** | **√** |  | **√** |
|  | **Time period**: baseline data were collected prior to 2002, during 2003 to 2006 2007 or later | **√** | **√** |  | **√** |
|  | **Age**: 15-49 years, 15-24 years, 25-49 years | **√** | **√** | **√** | **√** |
|  | **Pregnancy**: pregnancy status at baseline | **√** | **√** |  | **√** |
|  | **Cross-stratum (Region, Population type, Time period, Age, Pregnancy)**^1^ | **√** | **√** |  | **√** |
| **To evaluate the incidence of selected STIs: chlamydia, gonorrhea, and HSV-2** | **All studies** | **√** | **√** | **√** | **√** |
|  | **Region**: South Africa, Eastern Africa, Southern Africa | **√** | **√** |  | **√** |
|  | **Population type**: higher-risk or clinic/community-based populations | **√** | **√** |  | **√** |
|  | **Time period**: baseline data were collected prior to 2002, during 2003 to 2006 2007 or later | **√** | **√** |  | **√** |
|  | **Age**: 15-49 years, 15-24 years, 25-49 years | **√** | **√** | **√** | **√** |
|  | **Pregnancy**: pregnancy status at baseline | **√** | **√** |  | **√** |
|  | **Cross-stratum (Region, Population type, Time period, Age, Pregnancy)**^1^ | **√** | **√** |  | **√** |
| **Secondary** |  |  |  |  |  |
| **To evaluate the prevalence of co-infections of selected STIs/BV: gonorrhea, chlamydia, syphilis, trichomoniasis, HSV-2, and/or BV** | **All studies** |  | **√** |  | **√** |
| **To evaluate the incidence of co-infections among the STIs: chlamydia, gonorrhea, and HSV-2** | **All studies** |  | **√** |  | **√** |
| **Sensitivity Analysis** |  |  |  |  |  |
| **To evaluate the prevalence of gonorrhea, chlamydia, syphilis, and BV based on the type of diagnostic criteria** | **All studies** |  | **√** |  | **√** |
| **To evaluate the prevalence/incidence of STIs/BV based on the assessment of the risk of bias** | **All studies** |  | **√** |  | **√** |
| **To evaluate the incidence of STIs/BV based on HIV diagnosis** | **All studies** |  | **√** |  | **√** |
| **To evaluate the incidence of STIs/BV based on length of follow-up** | **All studies** |  | **√** |  | **√** |
| **To evaluate the prevalence/incidence of STIs/BV based on the study inclusion** | **All studies** |  | **√** |  | **√** |
| **To evaluate the incidence of STIs/BV based on censoring** | **All studies** |  | **√** |  | **√** |

^1^ If the size of analysis population within each cross-stratum is sufficient to conduct the meta-analysis
